# Turkish Society of Gastroenterology: Pancreas Working Group, Acute Pancreatitis Committee Consensus Report

**DOI:** 10.5152/tjg.2024.24392

**Published:** 2024-11-01

**Authors:** Deniz Öğütmen Koç, Göksel Bengi, Özlem Gül, Yeşim Özen Alahdab, Engin Altıntaş, Sezgin Barutçu, Yılmaz Bilgiç, Birol Bostancı, Mehmet Cindoruk, Kadri Çolakoğlu, Deniz Duman, Nergiz Ekmen, Ahmet Tarık Eminler, Yasemin Gökden, Süleyman Günay, Gözde Derviş Hakim, Kader Irak, Sabite Kacar, İsmail Hakkı Kalkan, Elmas Kasap, Aydın Şeref Köksal, Sedef Kuran, Nevin Oruç, Osman Özdoğan, Burak Özşeker, Erkan Parlak, Murat Saruç, İlker Şen, Gürhan Şişman, Mukaddes Tozlu, Nurettin Tunç, Nalan Gülşen Ünal, Hakan Ümit Ünal, Serkan Yaraş, Abdullah Emre Yıldırım, Müjde Soytürk, Dilek Oğuz, Orhan Sezgin

**Affiliations:** 1 Department of Gastroenterology, Health Sciences University, Gaziosmanpasa Education and Research Hospital, İstanbul, Türkiye; 2 Department of Gastroenterology, Dokuz Eylül University School of Medicine, İzmir, Türkiye; 3 Department of Gastroenterology, Lokman Hekim University School of Medicine, Ankara, Türkiye; 4 Department of Gastroenterology, Marmara University School of Medicine, İstanbul, Türkiye; 5 Department of Gastroenterology, Mersin University School of Medicine, Mersin, Türkiye; 6 Department of Gastroenterology, Gaziantep University School of Medicine, Gaziantep, Türkiye; 7 Department of Gastroenterology, Medipol University School of Medicine, İstanbul, Türkiye; 8 Department of Gastrointestinal Surgery, Health Sciences University, Ankara Bilkent State Hospital, Ankara, Türkiye; 9 Department of Gastroenterology, Gazi University School of Medicine, Ankara, Türkiye; 10 Department of Gastroenterology, Endoscopy Fulya, İstanbul, Türkiye; 11 Department of Gastroenterology, Sakarya University School of Medicine, Sakarya, Türkiye; 12 Department of Gastroenterology, Health Sciences University, Prof. Dr. Cemil Taşçıoğlu State Hospital, İstanbul, Türkiye; 13 Department of Gastroenterology, Health Sciences University, Katip Çelebi Education and Research Hospital, İzmir, Türkiye; 14 Department of Gastroenterology, Health Sciences University, İzmir School of Medicine, İzmir, Türkiye; 15 Department of Gastroenterology, Health Sciences University, Başakşehir Çam and Sakura State Hospital, İstanbul, Türkiye; 16 Department of Gastroenterology, Health Sciences University, Ankara Bilkent State Hospital, Ankara, Türkiye; 17 Department of Gastroenterology, TOBB University of Economics and Technology School of Medicine, Ankara, Türkiye; 18 Department of Gastroenterology, Celal Bayar University School of Medicine, Manisa, Türkiye; 19 Department of Gastroenterology, Güven Hospital, Ankara, Türkiye; 20 Department of Gastroenterology, Ege University School of Medicine, İzmir, Türkiye; 21 Department of Gastroenterology, Sıtkı Koçman University School of Medicine, Muğla, Türkiye; 22 Department of Gastroenterology, Hacettepe University School of Medicine, Ankara, Türkiye; 23 Department of Gastroenterology, Acıbadem University School of Medicine, İstanbul, Türkiye; 24 Department of Gastroenterology, Health Sciences University, Şişli Etfal Education and Research Hospital, İstanbul, Türkiye; 25 Department of Gastroenterology, Memorial Hospital, Diyarbakır, Türkiye; 26 Department of Gastroenterology, Arel University School of Medicine, İstanbul, Türkiye; 27 Department of Gastroenterology, Çanakkale Onsekiz Mart University School of Medicine, Çanakkale, Türkiye

**Keywords:** Acute pancreatitis, diagnosis, severity assessment, local pancreatic complications, treatment

## Abstract

Acute pancreatitis (AP) is a clinical condition that arises acutely in the pancreas through various inflammatory pathways due to multiple causes. Turkish Society of Gastroenterology Pancreas Working Group developed comprehensive guidance statements regarding the management of AP that include its epidemiology, etiology, clinical presentation, diagnostic criteria, disease severity, treatment, prognosis, local and systemic complications. The statements were developed through literature review, deliberation, and consensus opinion. These statements were ultimately used to develop a conceptual framework for the multidisciplinary management of AP.

## Introduction

Acute pancreatitis (AP) is a clinical condition that arises acutely in the pancreas through various inflammatory pathways due to multiple causes. Acute pancreatitis remains one of the most common gastrointestinal diseases requiring hospitalization worldwide. Despite advances in imaging techniques, treatment, and interventional procedures, it still has significant morbidity and mortality. Patients frequently present with pain in the epigastric region or upper abdominal quadrant that radiates to the back, along with nausea and vomiting. Approximately 80% of AP cases are mild and generally self-limiting. Severe forms are less common but have mortality rates approaching 30%. In the management of AP, both symptom control and the diagnosis and treatment of complications that arise during the course of the disease are of great importance. Therefore, the approach to patient management must be individualized. Currently, there are still controversial points regarding the etiopathogenesis, diagnosis, and treatment of the disease.

In this guide, we aim to address questions related to the definition, epidemiology, etiology, clinical presentation, diagnostic criteria, disease severity, treatment, prognosis, local and systemic complications of AP, and the management of these complications in light of current literature.

## The Stakeholders (Participants)

The Turkish Society of Gastroenterology—Pancreas Working Group has formed a sub-working group consisting of 38 experts to prepare the AP consensus report. The group held an initial informational meeting on January 5th, 2022 and began consensus report development meetings on February 27th, 2022. Target users of the consensus report are all clinicians involved in the follow-up and treatment of patients with AP.

## Methodology

As a first step in the preparation process of the AP consensus report, a coordination team specialized in AP was formed from the pancreas working group. This group’s systematic literature review provided evidence to address pre-determined topics (definition, etiology, diagnosis, disease severity, treatment, prognosis, local, and systemic complications). The group’s experience and views were integrated using an evidence-based methodology. The Delphi method was employed to ask the working group members to define research questions relevant to these topics. These questions were then consolidated and discussed face-to-face during a 1-day meeting, where they were finalized. During the same meeting, questions were tailored to fit a systematic literature search. As a result, a total of 49 questions were identified, comprising 10 main questions with their respective sub-questions. For each question, keywords for literature searches were specified. Decisions were made regarding the characteristics of articles to be included in the analysis, the evaluation criteria to be used during the analysis, and the method of analysis. This structured approach ensured the comprehensive and systematic gathering and evaluation of relevant evidence.

The members of the working group responsible for the systematic literature review received a half-day training on the review’s methodology, the selection of articles, the extraction of data from the articles, and the statistical methods to be used for combining and analyzing the obtained data.

Subsequently, each working group member responsible for the literature review conducted a systematic literature review related to their specific questions as described above. A literature search was conducted in PubMed/MEDLINE, the Cochrane Library, and Embase for relevant articles. Searches focused primarily on randomized controlled trials (RCTs) and meta-analyses. In addition, for topics not covered in these studies, retrospective analyses, case series, and prospective studies covering these topics were included. Inclusion criteria were determined as specific studies with a sample size of at least 20 patients, published in English and available in full text. Presented the results to the group during the second meeting. In the 2-day second meeting, the selected articles and the analysis of the data obtained from these articles were evaluated to answer each question. Draft recommendations were created for questions with sufficient data. For questions where the data were deemed insufficient by the working group, the missing analyses and additional analyses deemed necessary by the experts were identified.

The incomplete analyses were completed between the second and third meetings. During the 2-day third meeting, these analyses were presented to the working group by each member. Combining the evidence from the literature and the opinions of the working group, recommendations were formulated for each research question. For these recommendations, both the level of evidence and the recommendation grade were reported according to the Oxford criteria (Supplementary Table 1). Recommendations were prepared to be voted on by a larger group of gastroenterology experts related to the subject.

The final meeting was attended by 122 gastroenterologists from various provinces of Türkiye, including those working in university hospitals, government hospitals, and the private sector who are interested in AP. In this meeting, the results of the systematic literature review conducted for each question were presented along with the recommendations formed based on these results. Each recommendation was discussed by the group, and minor modifications were made if deemed necessary before being voted on. Recommendations that received an approval rate of 70% or higher from the group were accepted. Those that did not reach this approval rate were re-discussed, modified further, and voted on again. Ultimately, all recommendations were approved and accepted by the group with an approval rate of at least 70%. It was defined that “strong agreement” would require at least 80% of votes to be either “definitely yes” or “probably yes.”

Summary of the recommendations, level of evidence, and strength of recommendation are shown in Table 1.

## Questions and Recommendations

### 1. Introduction, Definition and Epidemiology

#### Question 1.1: What is the definition of AP?

**Recommendation 1.1:**

- Acute pancreatitis is an acute inflammatory disease of the pancreas caused by various factors. (Level of Evidence: 1A, Strength of recommendation: Strong consensus (89%)).

#### Question 1.2: What is the epidemiology of AP?

**Recommendation 1.2:**

- The incidence of AP has been steadily increasing over the past 50 years. The annual incidence ranges from 5 to 100 per 100,000. (Level of Evidence: 1A, Strength of recommendation: Strong consensus (97.5%)).

***Comment** **:*** While the incidence of AP is high in Northern Europe, Eastern Europe, and North America, the incidence in parts of Eastern Africa and South America is comparatively lower. While the incidence is rising in North America and Europe, it remains stable in Asia.^1,2^ When examining the distribution of etiology by region, gallstones are the predominant etiology in Southern Europe (Greece, Türkiye, Italy, Croatia), whereas alcohol is more prominent in Eastern Europe (Latvia, Lithuania, Russia, Finland, Hungary).^3^

#### Question 1.3: What are the risk factors for the development of AP?

**Recommendation 1.3:**

- Advanced age, male sex, smoking, obesity, elevated triglycerides (TG), pregnancy, and being of black race. (Level of Evidence: 1A, Strength of recommendation: Strong consensus (87.2%)).

***Comment:*** The incidence of AP increases with age. Particularly in the geriatric population, both the incidence and mortality rates are higher compared to younger age groups.^3,4^ Although AP is observed equally in both sexes, some studies report that its incidence is 1.5-2 times higher in men than in women.^5^ In terms of etiological distribution by gender, gallstones are more frequently seen in women, whereas alcohol and other etiological factors are more common in men.^6^ The prevalence of AP is 2-3 times higher in individuals of Black race and in Aboriginal populations compared to other races.^7^ Smoking also increases the risk of AP. Obesity contributes to an increased risk of gallstone-associated pancreatitis and severe pancreatitis. Elevated TG and an increase in body mass index (BMI) also elevate the risk of recurrent AP.^8-10^ 2. Etiology

#### Question 2: What is the etiology of AP?

**Recommendation 2:**

- The most common causes of AP are gallstones (40-70%) and alcohol (25-35%). The prevalence of these etiological factors can vary based on geographic, demographic, and genetic factors. Other causes include hypertriglyceridemia (HTG), endoscopic retrograde cholangiopancreatography (ERCP), medications, infectious agents, hypercalcemia, genetic variants, toxins, smoking, trauma, tumors, certain surgical procedures, and anatomical and physiological disorders of the pancreas. (Level of Evidence: 1A, Strength of recommendation: Strong consensus (94.1%)).

***Comment** **:*** The etiology of AP encompasses a broad spectrum. According to the results of a recent prospective cohort study that included 2244 patients across 17 centers, biliary AP ranks first in the etiology of AP in Türkiye (67.1%). This is followed by idiopathic (12%), hypertriglyceridemia (6%), and alcohol induced AP (4.2%).^11^ A meta-analysis of 46 studies from 36 different countries reported gallstones and alcohol as the primary etiological factors in AP.^12^ According to this study, biliary AP was reported at 42% (39-44), alcohol-induced AP at 21% (17-25), and idiopathic AP at 18% (15-22). However, the prevalence of etiological factors can vary based on geographical, demographic, and genetic factors.^13^ For example, while both gallstones and alcohol are the main etiological factors in Northern European countries, gallstones are the most common etiological factor in Southern European countries.

Gallstone pancreatitis is more common in women, whereas alcoholic pancreatitis is more frequently observed in middle-aged men.^14^ Anatomical variations and genetic predispositions can also contribute to the development of biliary pancreatitis.^15,16^

Alcohol is one of the most common causes of AP, and it has been found that the risk of AP increases with higher alcohol consumption.^17,18^ However, the incidence of AP among heavy alcohol users is reported to be only around 5%, suggesting that other accompanying factors (such as smoking, genetic, and anatomical factors) also play a role in the development of AP.^19,20^

Hypertriglyceridemia is one of the leading causes of AP. Serum TG levels, particularly those exceeding 1000 mg/dL, should be considered a potential cause of AP.

In addition, ERCP (16-97%), tumors (2-67%), drugs (8-41%), trauma (1-69%), hypercalcemia (2-16%), infectious agents (2-35%), and more rarely, genetic variants, toxins, smoking, anatomical and physiological disorders of the pancreas, and surgical interventions are included in the etiology of AP.^21,22^ A recent systematic review evaluating 128 publications reported that viral hepatitis (A, B, C, D and E) is the most common among the viruses causing AP with 34.4%, followed by coxsackie and echoviruses (14.8%), hemorrhagic fever viruses (12.4%), cytomegalovirus (12%), varicella-zoster virus (10.5%).^23^ Additionally, AP development associated with the new coronavirus disease (COVID-19) has also been reported.^24,25^ Studies have shown that severe acute respiratory syndrome (SARS) CoV-2 infects human endocrine and exocrine pancreatic cells, suggesting a direct role of SARS-CoV-2 in pancreatic disorders.^26^ Besides viruses, bacteria (*Mycobacterium tuberculosis*, mycoplasmas, leptospirosis), parasites (*Ascaris lumbricoides*, *Fasciola hepatica*, and echinococcal cysts), and fungal infections (aspergillosis) are also etiological factors causing AP.^27^ An AP course associated with infectious agents has reported a mortality rate of 20%, which is higher than those reported for other etiologies. This situation is mostly associated with immunosuppression.^23^

Smoking also increases the risk of AP. The risk is higher in active smokers (Hazard Ratio (HR), 1.75; 95% Confidence interval (CI), 1.26-2.44); however, the risk persists in former smokers (HR, 1.63; 95% CI, 1.18-2.27).^28^ Smoking additionally elevates the risk of alcohol-induced, idiopathic, and drug-induced pancreatitis, but no effect on biliary pancreatitis has been observed. Each additional 10 cigarettes smoked per day increases the risk of AP by 40%.^29,30^

Genetic factors play both direct and indirect roles in the etiology of AP. In individuals with early onset of AP and a family history following an autosomal dominant pattern, mutations in the serine protease 1 (PRSS1) gene should be investigated. Serine protease inhibitor Kazal type 1 (SPINK1) binds to prematurely activated intracellular trypsin, playing a protective role against pancreatitis. A meta-analysis showed that the p.N34S variant in this gene is more prevalent in patients who have experienced AP (Odds Ratio (OR) = 3.16, *P* < .001).^31,32^ Mutations in the cystic fibrosis transmembrane conductance regulator (CFTR) gene actually facilitate AP and are also a cause of chronic pancreatitis (CP). Some metabolic storage diseases, such as Gaucher disease, can also be counted among the genetic causes of AP.^33^

It is known that endoscopic or surgical interventions such as double balloon endoscopic examination, ERCP and intragastric balloon application can also lead to the development of AP.^34,35^ The incidence of AP following ERCP is reported to be approximately 3.5%. When ERCP is performed to treat sphincter of Oddi dysfunction, the risk of causing AP is higher. Other risk factors for the development of post-ERCP pancreatitis include younger age, female gender, the number of attempts to cannulate the papilla, and inadequate drainage of the pancreatic duct following the injection of contrast material.

Abdominal, cardiac, spinal surgeries, and vascular embolectomies can also lead to AP. Particularly during major vascular interventions, ischemia of the pancreas, emboli to vascular structures supplying the pancreas, direct injuries caused by retractors or incisions, and crush syndrome can cause AP.^36-38^

The most common drugs causing AP are azathioprine, 6-mercaptopurine, valproic acid, thiazides, tamoxifen, and exogenous estrogens. Pancreas divisum (PD) is the most frequently encountered anatomical variation of the pancreatic duct and is more commonly associated with recurrent AP.^39-41^ An arteriovenous shunt in the pancreas can lead to recurrent AP by causing ischemia or bleeding.^42^ Duodenal duplication cysts and juxtapapillary diverticula are also causes of AP.^43,44^ Metabolic conditions such as hypercalcemia and hyperparathyroidism, as well as parathyroid carcinoma, and benign or malignant mass lesions obstructing the main pancreatic duct, can cause AP.^45,46^ Systemic lupus erythematosus (SLE), hemolysis, arteriovenous malformations, venoms, and toxins are other causes of AP. Systemic lupus erythematosus–related pancreatitis can result from vasculitis, microthrombosis, anti-pancreatic autoantibodies, drug side effects, intimal thickening, and concurrent viral infections.^47^ In pregnancy, AP can also occur due to causes such as gallstones and HTG.^48^

### 3. Diagnostic Criteria (Laboratory, Clinical and Imaging)

#### Question 3.1: How is the diagnosis of AP made?

**Recommendation 3.1:**

- The diagnosis is based on the presence of typical abdominal pain, laboratory findings including an elevation of amylase and/or lipase levels more than 3 times the normal, and supportive findings from imaging modalities such as transabdominal ultrasonography (TAUS), computed tomography (CT), or magnetic resonance imaging (MRI). Patients with 2 out of these 3 criteria are diagnosed with AP. (Level of Evidence: 1A, Strength of recommendation: Strong Consensus (99.2%)).

***Comment** **:*** Abdominal pain is the primary symptom in AP, present in over 95% of patients.^49,50^ The typical abdominal pain associated with AP begins in the epigastric region or upper quadrant of the abdomen, radiating to the back. This pain is generally dull and severe, partially alleviated in the knee-chest (fetal) position, and intensified by eating or drinking. This type of pain occurs in 40-70% of patients.^2^ The second most frequent symptom is nausea and/or vomiting, which occurs in 90% of patients.^51^ Gastroparesis and localized or generalized ileus, resulting from peripancreatic inflammation, are responsible for the nausea and vomiting. Additionally, symptoms such as fever, tachycardia, distension, jaundice, and dyspnea may also be present to varying degrees.

The threshold value for amylase and lipase in the diagnosis of AP is 3 times the normal level, with sensitivities and specificities of 72% and 93% for amylase and 79% and 89% for lipase, respectively.^52,53^ A review comparing amylase and lipase in the diagnosis of AP indicated that the specificities of these tests are similar (around 90%), but lipase has a higher sensitivity (amylase sensitivity ranges from 45% to 85%, while lipase sensitivity ranges from 55% to 100%).^54^ In cases of AP due to hyperlipidemia or in acute attacks of CP, amylase and lipase levels may not be elevated.^55^ Studies have shown that biomarkers such as phospholipase, elastase, and carboxypeptidase have lower sensitivities and specificities compared to amylase and lipase in diagnosing AP.^56^ Additionally, these biomarkers are not widely used in clinical practice due to disadvantages in terms of time, cost, and application. However, studies on the urinary trypsinogen-2 test have shown that its sensitivity and specificity for diagnosing AP exceed 82% and 90%, respectively, with levels rising within a few hours after the onset of AP.^57^

#### Question 3.2: What is the role of imaging in the diagnosis of AP?

**Recommendation 3.2:**

- Imaging methods are 1 of the 3 diagnostic criteria and are crucial in diagnosing AP. They play a significant role when the clinical and laboratory diagnosis of AP remains uncertain or when other potential conditions (such as organ perforation, mesenteric ischemia, ileus, etc.) are being considered. Transabdominal ultrasonography can be used as a primary imaging method due to its ability to provide valuable information not only for diagnosing AP but also for etiological assessment (differentiating between biliary and non-biliary causes), coupled with its widespread use. If the diagnosis of AP remains uncertain after TAUS, evaluation with CT or MRI is recommended. (Level of Evidence: 2B, Strength of recommendation: Strong consensus (89.4%)).

***Comment** **:*** In the early stages of AP, radiological findings may not be pronounced and can even appear normal.^58^ However, imaging can reveal features such as focal or diffuse pancreatic enlargement, irregular contours, parenchymal heterogeneity, increased density of peripancreatic fat planes, and intraperitoneal or retroperitoneal fluid collections.^59^ A meta-analysis comparing CT and MRI in diagnosing AP indicated that MRI is superior to CT in terms of sensitivity and specificity. According to this meta-analysis, the diagnostic sensitivity of MRI for AP is 92% and its specificity is 74%, while CT has a sensitivity of 73% and a specificity of 64%.^60^ Additionally, a study assessing mild forms of AP found that MRI is particularly superior to CT in demonstrating peripancreatic inflammation.^61^ Magnetic resonance imaging offers additional advantages over CT due to its high resolution and lack of radiation exposure. Nevertheless, MRI has limitations, including long acquisition times, higher costs, less widespread availability, motion artifacts, inability to allow for interventional therapeutic procedures, and lower sensitivity in detecting gas bubbles and calcifications. There are also studies indicating that in cases of mild or uncomplicated AP, methods such as CT or MRI do not provide additional benefits.^62^

In the diagnosis of AP, TAUS should be the imaging method of first choice. However, it should be noted that in patients with atypical pain, severe pancreatitis, or suspected complications, TAUS may not fully replace CT or MRI.^63^ Additionally, conventional TAUS is not as sensitive as CT and MRI in detecting pancreatic necrosis and masses.^58^ While TAUS has a sensitivity of 95% for detecting cholelithiasis, its sensitivity for detecting choledocholithiasis ranges between 50-80%.^64^

### 4. Severity of AP

#### Question 4.1: How is the severity of AP categorized?

**Recommendation 4.1:**

- The severity of AP is categorized as mild, moderate, or severe based on the presence of local and systemic complications, as well as the state of necrosis and infected necrosis. The Revised Atlanta Classification is the most commonly used classification for this purpose. (Level of Evidence: 1A, Strength of recommendation: Strong consensus (96.7%)).

***Comment** **:*** The severity of AP is categorized as mild, moderate, or severe based on the presence of local and systemic complications, as well as the state of necrosis and infected necrosis. The Revised Atlanta Classification (Supplementary Table 2) is the most commonly used classification for this purpose, categorizing AP as follows:

- Mild AP (interstitial edematous pancreatitis): There is no organ failure, and no local or systemic complications. It generally resolves within the first week.
- Moderate AP: There is transient organ failure that resolves within 48 hours.
- Severe AP: There is persistent organ failure involving one or more organs.^65^

Evaluating the severity of the disease solely based on clinical signs and symptoms is often unreliable and should be supported by objective measures. It is important to classify the severity of AP early, as patients with AP are at risk of developing persistent organ failure. Additionally, mortality rates differ among subtypes of AP. For instance, the mortality rate for mild edematous AP is 1%, whereas it reaches 15-25% for severe necrotizing AP.^66,67^ To reduce the mortality rate and improve prognosis in severe AP, it is crucial to assess the severity of AP early in the disease course, initiate appropriate treatment based on etiology, recognize pancreatitis complications early, and determine the need for intensive care unit (ICU) admission.

#### Question 4.2: Are there clinical-radiological scoring systems and biochemical markers that can aid in the early identification of severe AP?

**Recommendation 4.2:**

- Rapid and accurate prediction of severe AP is essential for improving patient prognosis.
  - There is insufficient evidence and consensus on a “gold standard” biochemical parameter or prognostic score for predicting severe AP.
  - Elevated blood urea nitrogen (BUN) and serum creatinine levels during the course of AP or at 48 hours are associated with increased morbidity and mortality.
  - C-reactive protein (CRP) level of 150 mg/L (15 mg/dL) at 48 hours can be used as an indicator of poor prognosis in AP.
  - Given its simplicity in calculation and comparability to the Acute Physiology and Chronic Health Evaluation II (APACHE II) score, the Bedside Index for Severity in Acute Pancreatitis (BISAP) score is the recommended scoring system for routine clinical practice.
  - Imaging-based indices such as the computed tomography severity index (CTSI) and modified CTSI (mCTSI) can be useful in predicting severe AP and persistent organ failure due to their high positive predictive values. (Level of Evidence: 3, Strength of recommendation: Strong consensus (91.7%)).

***Comment** **:*** Severe AP has high morbidity and mortality rates, necessitating the early identification of potential cases for aggressive treatment.^52,68^ Rapid and accurate prediction of the progression of severe AP is essential to improve patient prognosis.^69^ Although numerous studies have been conducted on various parameters and scoring systems, there is still no sufficient evidence or consensus on a “gold standard” biochemical parameter or prognostic score for predicting severe AP.

To identify severe AP, multiple scoring systems with varying accuracy and low positive predictive values exist, none of which exhibit very high sensitivity or specificity. These include the Ranson criteria, CTSI, APACHE II score, Glasgow system, Harmless Acute Pancreatitis Score (HAPS), PANC 3, Japanese severity score (JSS), pancreatitis outcome prediction (POP), and BISAP score. Bedside index for severity in acute pancreatitis is one of the most accurate and applicable scoring systems in daily clinical practice because it is simpler than traditional scoring methods, can be used within the first 24 hours, and closely predicts AP severity, organ failure, and mortality, similar to the complex APACHE II system. A BISAP score greater than 2 is sensitive for predicting severe AP (Area under the curve (AUC) 0.76-0.96; 61-97.6%), morbidity (AUC 0.67-0.93; 40-89%), and mortality (AUC 0.79-0.97; 75-100%). Mortality is below 1% with a BISAP score of 0, but it reaches 22% when the score is 5 (Supplementary Table 3).^70-79^

There are publications indicating that the rise in BUN and creatinine levels within the first 48 hours of AP suggests that pancreatitis will likely be severe, with high morbidity and mortality.^71,72,80^

Serum CRP and procalcitonin (PCT) levels can also be useful in predicting the severity of AP. A CRP level higher than 150 mg/L (15 mg/dl) at 48 hours from symptom onset has an 86% sensitivity and a 61% specificity in predicting the severity of AP.^81^ Similarly, a meta-analysis evaluating PCT as a diagnostic marker in severe AP found a sensitivity of 0.84, a specificity of 0.81, a diagnostic odds ratio of 21.26, and an AUC of 0.89.^82^ Another meta-analysis reported sensitivity, specificity, and AUC values of 0.73, 0.87, and 0.88, respectively, when using a PCT threshold value greater than 0.5 ng/mL, indicating that serum PCT is a reliable indicator of severe AP.^83^ Indices associated with imaging methods such as the Extra-pancreatic Inflammation on CT (EPIC) score, CTSI, and mCTSI can also be useful in predicting the severity of AP.^74-79,84^

### 5. Treatment of Acute Pancreatitis

It is important to initiate AP treatment early. In these patients, the basis of treatment includes pain relief, fluid replacement, combatting infections, providing nutritional support, tailoring treatment to the etiology, and addressing complications that arise during the course of the disease.

#### Question 5.1: How should fluid therapy be done in AP?

**Recommendation 5.1:**

- Early fluid therapy is important in the treatment of AP.
  - The fluid used in treatment should be isotonic crystalloid (isotonic NaCl or Ringer’s lactate (RL)). If there is no contraindication specific to the patient (e.g., hypercalcemia), RL can be preferred.
  - There is insufficient evidence regarding the use of hydroxyethyl starch (HES) in AP treatment. Its use is not recommended in AP treatment except for abdominal compartment syndrome.
  - The rate of fluid resuscitation should be tailored according to the patient’s clinical assessment at presentation and follow-up data (targeted).
  - Aggressive fluid therapy in AP, particularly in moderate to severe and severe AP patients, is not recommended as it increases the risk of systemic inflammatory response syndrome (SIRS), organ failure, the need for intensive care and ventilation, and the development of abdominal compartment syndrome. (Level of evidence: 1A, Strength of recommendation: Strong consensus (94.7%)).

***Comment** **:*** The correct management of fluid therapy in patients with AP is crucial. The period encompassing the first 72 hours from the onset of symptoms, referred to as the “golden hours,” is particularly critical. During this period, the treatment of intravascular hypovolemia, which can result from a severe inflammatory response, can be achieved with personalized, appropriate fluid support.^85^ Fluid therapy is especially important in severe AP due to its impact on early mortality and morbidity. Intravenous (IV) fluid therapy should be initiated immediately and at the highest possible targeted dose in patients diagnosed with AP or those being evaluated with a preliminary diagnosis of AP. The targeted initial fluid therapy should be determined by the attending physician based on clinical data at the time of presentation, such as cardiovascular or respiratory failure, hypo/hypervolemic status, renal failure, and hypercalcemia. While the definition of aggressive fluid therapy varies across studies, it can generally be characterized as IV hydration at a rate of 3 mg/kg/hour or more, independent of the initial bolus fluid loading therapy. Although previous studies found aggressive fluid therapy beneficial, its current use is not recommended in severe AP patients due to the potential for causing SIRS, organ failure, and abdominal compartment syndrome, as well as increasing the need for intensive care and ventilation.^86-88^ The rate of maintenance fluid therapy following the initial treatment should be determined by evaluating the patient’s clinical data, such as urinary volume, and signs of respiratory and circulatory failure.

In patients with AP, the fluid administered for replacement therapy should be an isotonic crystalloid solution (RL or normal saline). There is no difference between these 2 fluid therapies concerning mortality, local complications, or inflammatory parameters.^89-92^ However, a reduced need for intensive care has been observed in patients treated with RL. Although this effect is thought to be due to the anti-inflammatory properties of the lactate in RL, no significant difference in inflammatory parameters has been found between patients given isotonic normal saline and those given RL. Additionally, it has been observed that administering large volumes of normal saline in a short period increases metabolic acidosis.^93,94^ Thus, if there are no contraindications such as hypercalcemia, RL should be the first choice of fluid in treatment.

There is insufficient data regarding the use of osmotically active fluids like HES in the treatment of AP. Although some studies have shown that HES can reduce intra-abdominal pressure in patients who develop compartment syndrome, it has not been found to have an effect on mortality or inflammatory parameters.^95,96^ In a study involving 7000 patients admitted to intensive care for any reason, it was observed that those who received HES had an increased need for renal replacement therapy.^97^ Therefore, while HES can be added to treatment if abdominal compartment syndrome is present in severe cases of AP, its routine use is not recommended.

#### Question 5.2: How should the medical treatment of pain in AP be?

**Recommendation 5.2:**

- Pain in AP is usually severe, necessitating pain control in most patients.
- There is no sufficient evidence or consensus on the optimal analgesic and route of administration for pain associated with AP.
- In the first 24 hours of AP treatment, opioid and non-opioid analgesics have similar efficacy and safety profiles.
- Although opioid analgesics (Buprenorphine, pethidine, fentanyl, pentazocine, morphine, tramadol) are effective, special caution should be exercised regarding pethidine and morphine due to their side effects.
- In patients with mild AP, non-steroidal anti-inflammatory drugs (NSAIDs) (Indomethacin, metamizole, dexketoprofen, diclofenac) have similar efficacy to opioids in pain palliation during the first 24 hours and can be used as alternatives to opioids. They should not be used in patients with renal failure.
- Although rarely used for pain palliation in the first 24 hours, epidural analgesic applications have been found effective. They can be employed as alternatives to or in combination with opioids before transitioning to other treatments. (Level of Evidence: 1A, Strength of recommendation: Strong consensus (95.6%)).

***Comment** **:*** Abdominal pain is the main symptom in almost all patients with AP who present to the hospital.^98^ The pain is often severe and requires effective medical management. Early and adequate pain control within the first 24 hours of hospitalization in patients diagnosed with AP improves quality of life and reduces patient anxiety, respiratory stress, hospital stay duration, and the risk of AP-related complications.^99^ Additionally, early analgesic use has been shown not to delay the diagnosis and treatment of AP.^100^ Although there are many pharmacological treatment options for managing pain in AP, opioid analgesics are the most commonly used. Agents such as buprenorphine, pethidine, morphine, and fentanyl can be administered parenterally. However, uncertainties remain regarding the clinical efficacy and safety of opioids. Since abdominal pain in AP is due to parenchymal inflammation, NSAIDs are used in pain management by inhibiting prostaglandin synthesis through targeting the cyclooxygenase (COX) enzyme. Non-steroidal a nti-inflammatory d rugs are not frequently preferred due to potential renal damage and gastrointestinal system (GIS) complications, but they have been shown to reduce pro-inflammatory cytokines and oxidative stress, improve histopathological changes, and decrease potential systemic complications.^101^ Local anesthetics (e.g., procaine, bupivacaine) and paracetamol are also used less frequently to treat pain in AP.^102^ When local anesthetics are used systemically, they provide pain control through anti-inflammatory, neuroprotective, and motility-regulating effects.^103^ Epidural analgesia has been shown to improve pain scores, increase capillary perfusion in the GIS mucosa, prevent sepsis, and reduce the risk of respiratory depression in AP.^104^

The primary concern related to opioids is their potential to complicate the disease by causing sphincter of Oddi spasm. While it is suggested that this increase in pressure is associated with the plasma concentration and dosage of opioids, the clinical significance of this relationship remains unclear. This ambiguity arises because many studies are small-scale observational studies, and there is a lack of definitive evidence from controlled clinical trials supporting this theory.^105^ Additionally, side effects such as respiratory depression, paralytic ileus at high doses, and the widespread issue of opioid addiction necessitate the search for alternative treatments in the management of AP. Despite some evidence from RCTs, there is still no consensus on the most appropriate analgesics, their dosages, administration methods, and frequencies for treating pain associated with AP.

While optimal treatment strategies for managing pain associated with AP continue to be explored, 2 meta-analyses have been published in the last 2 years on this subject. The first is a meta-analysis by Thavanesan et al^106^, which evaluated 12 RCTs involving a total of 542 AP patients and reported significant methodological heterogeneity. The included studies compared opioids, NSAIDs, local anesthetics, epidural analgesia, paracetamol, and placebo for pain management in AP. This meta-analysis revealed that epidural analgesia provided the greatest improvement in VAS scores during the first 24 hours, although its effectiveness plateaued and became comparable to opioids at 48 hours. Continuous epidural analgesia infusion is not recommended for mild to moderate AP cases due to potential side effects like hypotension related to catheter placement and epidural abscesses. Additionally, NSAIDs provided similar pain relief to opioids in the first 24 hours, while local anesthetics were the least effective among all treatment agents in terms of pain palliation. Overall, comparisons of VAS score improvements at baseline and on day 1 indicated that opioids and non-opioids were similarly effective.

In a meta-analysis published by Cai et al^107^ in 2021, 12 RCTs involving a total of 699 patients were evaluated to assess the effectiveness of pain management in AP, with the primary endpoint being the number of patients requiring rescue analgesia. Among the included patients, 83% had mild AP. Both opioid and non-opioid analgesics reduced the need for a second opioid analgesic as rescue medication without significantly altering pain scores in the first 24 hours. Based on the results of studies with high heterogeneity, it was observed that the need for rescue analgesia was lower in the opioid group compared to the non-opioid group, although there was no significant difference in the changes in VAS scores between the 2 groups within the first 24 hours.^108^ Other subgroup analyses demonstrated no significant differences in efficacy and side effect rates between opioids and NSAIDs. In light of these findings, NSAIDs may be preferred over opioids as the first-line treatment for pain palliation in AP patients. However, due to the moderate quality and high heterogeneity of the included RCTs, a high-level recommendation for pain palliation in AP cannot be made. The heterogeneity among the studies is primarily due to differences in the routes of administration and dosages of the analgesics used.

According to a review by Wu et al^101^ in 2020, which evaluated the use of NSAIDs in the treatment of pain in AP across 36 studies (including 5 clinical trials with 580 patients and 31 animal studies), NSAIDs were found to reduce pro-inflammatory cytokines, pain, systemic complications, and mortality rates, with a very low likelihood of serious side effects.

In conclusion, there is insufficient evidence and consensus regarding the most appropriate analgesic and route of administration for the treatment of pain associated with AP. Within the first 24 hours of AP treatment, opioids and non-opioid analgesics exhibit similar efficacy and safety profiles. For the palliation of pain in mild to moderate cases of AP, both NSAIDs and opioids can be considered appropriate options. While opioids are generally used for pain palliation in patients with severe AP, there is a lack of sufficient evidence to determine the optimal pain management strategy.

#### Question 5.3: How should nutrition be managed in AP?

**Recommendation 5.3:**

- Unless there is an obstruction or contraindication to oral feeding (e.g., ileus, abdominal compartment syndrome), oral intake should not be discontinued. (Level of Evidence: 1B, Strength of recommendation: Weak consensus (78.4%)).
- If oral feeding cannot be initiated within the first 72 hours, nutritional support should be provided. For patients who cannot tolerate oral feeding, enteral nutrition (EN) should be prioritized. Feeding should commence using a nasogastric (NG) or nasojejunal (NJ) tube. (Level of Evidence: 1A, Strength of recommendation: Weak consensus (79%)).
- For patients who cannot tolerate EN, where NG/NJ tube placement is not possible, or where target protein and calorie needs cannot be met by EN alone, parenteral nutrition (PN) should be administered. (Level of Evidence: 1A, Strength of recommendation: Strong consensus (89.9%)).
- Glutamine should be added to the nutritional solution for patients requiring nutritional support. (Level of Evidence: 1A, Strength of recommendation: Strong consensus (84.9%)).

***Comment** **:*** Despite the known positive impact of oral feeding on the course of AP, there is no consensus regarding the optimal time to initiate oral feeding. Recently, RCTs and meta-analyses based on these studies have been added to the literature, suggesting that oral intake should not be discontinued unless there is intolerance, contraindication, or another barrier to oral feeding.^109-112^ No significant differences in the incidence of SIRS or the exacerbation of disease symptoms have been reported between patients who started oral feeding at the earliest possible time and those whose oral feeding was delayed.^110^ In cases of mild AP, early oral feeding has been found to be safe and may accelerate recovery. These studies have shown that starting a normal solid diet in patients with mild AP reduces the duration of hospital stay and does not increase abdominal pain.

In patients who cannot tolerate oral feeding, the first choice should be EN. Enteral nutrition maintains the integrity of the intestinal mucosa, stimulates gut motility, prevents bacterial overgrowth, and increases splanchnic blood flow.^113^ Several RCTs and meta-analyses have demonstrated the superiority of EN over PN in the management of AP.^114-119^ Enteral nutrition has been found to reduce septic complications and inflammation more rapidly than PN, while also being cost-effective.^118^ Another meta-analysis comparing EN and PN found no differences in mortality and non-infectious complications, but EN was superior in terms of infections, surgical intervention requirements, and length of hospital stay.^117^ Additionally, 1 RCT noted that EN reduced infectious complications, multiple organ dysfunction syndrome (MODS), and mortality in patients with infected pancreatic necrosis, although other studies have reported no difference between EN and PN.^120,121^ It has been shown that initiating EN early (within 24-48 hours) is feasible, safe, well-tolerated, and provides significant clinical benefits over delayed EN in terms of mortality, organ failure, and infectious complications.^122-129^

For EN, either NG or NJ routes can be used. A meta-analysis found that, in patients with severe AP, NG and NJ feeding were similar in terms of mortality rate, tracheal aspiration, diarrhea, exacerbation of pain, and energy balance.^130^ The placement of NG tubes is significantly easier, more comfortable, and less expensive.^131,132^

In EN, both semi-elemental and polymeric feeding formulas can be used. Although both types of formulas are well tolerated in patients with AP, semi-elemental nutrition is thought to have more favorable clinical effects; however, the level of evidence supporting this is weak.^133^ It is recommended that enteral feeding be initiated with standard polymeric formulas in patients with severe AP.^134^

In patients who cannot tolerate EN, cannot have an NG/NJ tube placed, or cannot meet their target protein and calorie needs with EN alone, PN should be administered. While glutamine supplementation is not necessary for patients receiving EN, those on PN should be supplemented with 0.20 g/kg of L-glutamine daily.^135,136^ Studies have shown that glutamine supplementation in patients with AP has positive effects on serum albumin levels, CRP, infectious complications, length of hospital stay, and mortality.^137-140^ Apart from glutamine, immunonutrition has no established role in severe AP.

The addition of probiotics to the nutrition of patients with AP has not been shown to provide significant benefits in terms of pancreatic infection, systemic infection, the need for surgery, length of hospital stay, or mortality. In fact, one study observed higher mortality in the probiotic group.^141,142^

In patients with severe AP, nutritional support should provide 25-35 kcal/kg/day of energy, 1.2-1.5 g/kg/day of protein (unless there is renal failure or severe liver failure), 3-6 g/kg/day of carbohydrates, and up to 2 g/kg/day of lipids. Daily supplementation with multivitamins and trace elements is also recommended.^142^

### 5.4. Antibiotic Treatment

#### Question 5.4.1: In what situations should systemic antibiotic treatment be initiated in AP?

**Recommendation 5.4.1:**

- The use of prophylactic antibiotics is not recommended in AP, including severe pancreatitis and the presence of necrosis. However, antibiotics are recommended in cases of infected necrosis and extrapancreatic infections. (Level of Evidence: 1A, Strength of recommendation: Strong consensus (89.1%)).

***Comment** **:*** In meta-analyses conducted before the year 2000, which included a small number of patients, it was reported that the use of prophylactic antibiotics in AP reduced mortality.^143-145^ However, results from meta-analyses and systematic reviews published from 2000 onwards have shown that routine prophylactic antibiotic use has no effect on mortality, morbidity, length of hospital stay, or the need for surgery in AP cases.^143,146-153^ In light of these findings, routine prophylactic antibiotic use is not recommended during AP attacks, regardless of the type (interstitial or necrotizing) or severity of pancreatitis. Nevertheless, approximately 20% of AP patients may develop extrapancreatic infections such as pneumonia, urinary tract infections, bacteremia, or acute cholangitis.^154^ Since these extrapancreatic infections are associated with increased mortality and morbidity, appropriate antibiotic treatment is recommended. If culture results are negative or no infectious focus is found, discontinuation of antibiotic use is advised.^155^

Antibiotic therapy is recommended in the presence of infected necrosis.^155^ There is no correlation between the extent of necrosis and the frequency of infection. Although infection typically appears around 10 days after the onset of necrosis, it can also occur in its early stages.^156,157^ Fungal infections are detected in 6-46% of bacterial cultures taken from sites of infected necrosis.^158^ However, the impact of prophylactic antifungal treatment on prognosis and mortality is unclear. Therefore, prophylactic antifungal treatment is also not recommended.^159^

#### Question 5.4.2: Which antibiotics should be preferred in AP?

**Recommendation 5.4.2:**

- In AP, carbapenems, quinolones, metronidazole, and cephalosporins can be used. In the presence of infected necrosis, carbapenem antibiotics should be preferred. (Level of Evidence: 1A, Strength of recommendation: Strong consensus (95.4%)).

***Comment** **:*** Infected necrosis pathogens are typically of intestinal origin (*Escherichia coli, Pseudomonas, Klebsiella, Enterococcus*) and are usually monomicrobial. The presence of gas in the necrotic area on imaging supports infection and necessitates antibiotic treatment. Very few antibiotics can penetrate pancreatic necrosis. Studies on antibiotic use in acute necrotizing pancreatitis have shown the use of imipenem, meropenem, a combination of ciprofloxacin and metronidazole, or ciprofloxacin alone. According to the results of these studies, carbapenem antibiotics should be preferred first due to their higher pancreatic penetration.^160-165^

### 5.5. Treatments Targeting Etiology

#### Question 5.5.1: When should ERCP be performed in patients with Acute Biliary Pancreatitis?

**Recommendation 5.5.1:**

In acute biliary pancreatitis, if there are signs of a stone impacted in the papilla or cholangitis, ERCP is recommended at the earliest possible stage. If these conditions are not present but there are signs of cholestasis, imaging of the common bile duct (endoscopic ultrasonography (EUS), magnetic resonance cholangiopancreatography (MRCP)) is recommended. (Level of Evidence: 1A, Strength of recommendation: Strong consensus (100%)).

***Comment*** **:** In cases of acute biliary pancreatitis where ERCP is indicated, there remains uncertainty in the literature regarding whether the procedure should be performed within 24 hours or within 72 hours. The timing of endoscopic intervention should be determined based on the patient’s clinical condition, comorbidities, and medications they are taking. A recent meta-analysis by Iqbal et al^166^ found that performing ERCP within the first 48 hours in cases of acute cholangitis significantly reduced in-hospital mortality, 30-day mortality, and hospital stay duration.

According to a Cochrane analysis conducted in 2012, early ERCP (<72 hours) in cases of acute cholangitis with biliary pancreatitis is superior to conservative treatment or elective ERCP in terms of mortality, hospital stay, and morbidity. In cases of biliary obstruction without cholangitis, early ERCP is also superior to conservative treatment or elective ERCP in reducing morbidity and preventing the development of local and systemic complications.^167^ A review by Shuntaro Mukai et al^168^ indicated that performing ERCP in patients with ongoing cholangitis and biliary obstruction significantly reduces mortality, morbidity, local complications, and sepsis compared to conservative treatment. According to the Tokyo 2018 guidelines, the diagnosis of acute cholangitis is established through clinical, laboratory, and imaging methods (fever and/or chills, elevated CRP levels, leukocytosis or other elevated inflammatory parameters, jaundice, and a 1.5-fold increase in aspartate transaminase (AST), alanine transaminase (ALT), alkaline phosphatase (ALP), gamma-glutamyl transpeptidase (GGT) levels, with biliary dilation detected on imaging).^169^

According to a meta-analysis involving cases of biliary pancreatitis without cholangitis and impacted bile stones, early ERCP does not significantly differ from conservative treatment in terms of mortality (OR: 0.59, 95% CI: 0.32-1.09; *P* = .09), complication development (OR: 0.56, 95% CI: 0.30-1.01; *P* = .05), new-onset organ failure (OR: 1.06, 95% CI: 0.65-1.75; *P* = .81), development of pancreatic necrosis (OR: 0.80, 95% CI: 0.49-1.32; *P* = .38), development of pancreatic pseudocyst (PP) (OR: 0.44, 95% CI: 0.16-1.24; *P* = .12), ICU admission (OR: 1.64, 95% CI: 0.97-2.77; *P* = .06), and pneumonia development (OR: 0.81, 95% CI: 0.40-1.65; *P* = .56).^170^ Therefore, it is essential to assess the presence of stones in the biliary tract and plan ERCP for necessary cases. Endoscopic ultrasonography and MRCP are commonly used investigations for evaluating stones in the biliary tract. Endoscopic ultrasonography is particularly valuable for stones smaller than 5 mm. The sensitivity and specificity of EUS and MRCP for detecting stones in the biliary tract are 97% vs. 90% and 87% vs. 92%, respectively.^171^

#### Question 5.5.2: How should HTG-induced AP treatment (beyond standard treatment) be administered? What are the treatment options?

**Recommendation 5.5.2:**

- It is recommended to add insulin infusion to the treatment of HTG-induced AP (Level of Evidence: 1A, Strength of recommendation: Strong consensus (91.2)).
- There is insufficient evidence on the additional benefit of adding heparin infusion to insulin infusion (Level of Evidence: 3, Strength of recommendation: Weak consensus (74.3%)).
- Plasmapheresis has not been shown to provide additional benefit when combined with insulin infusion (Level of Evidence: 1A, Strength of recommendation: Weak consensus (74.3%)).

***Comment** **:*** In HTG-induced AP, additional treatments beyond standard pancreatitis therapy include the administration of insulin and/or heparin, and plasmapheresis. Insulin aids in lowering TG levels by increasing peripheral lipoprotein lipase (LPL) activity. Specifically, an IV insulin infusion at 0.1-0.4 units/kg/hour is preferred over subcutaneous (SC) insulin due to its easier monitoring and dose planning.^172,173^ A meta-analysis involving 118 cases indicated that, although the number of cases in the included studies was limited, intensive insulin therapy significantly reduced APACHE II scores at the 72-hour mark of treatment.^174^ In a study comparing insulin therapy and conservative AP treatment in HTG-induced AP (HTG-AP), TG reduction on days 2 and 4 were 69% vs. 85% and 63% vs. 79%, respectively, with no significant difference detected between the groups.^175^

Heparin also causes the release of LPL from endothelial cells, leading to a reduction in TG levels; however, prolonged administration of heparin results in the depletion of LPL stores, decreased chylomicron catabolism, and rebound HTG.^176^ In a retrospective study comparing insulin and heparin treatments, insulin was found to have a greater TG-lowering effect than heparin in cases of edematous pancreatitis, with no differences in complications observed between the 2 groups.^177^

Plasmapheresis treatment has been compared with insulin infusion and/or heparin therapy in numerous studies. In a 2022 meta-analysis by Yan LH et al^178^, although a significant reduction in TG levels at 24 hours was observed with plasmapheresis compared to conventional therapy, no differences were found in hospital stay duration, mortality, or morbidity. Another meta-analysis evaluating 934 patients also found no differences in efficacy and safety between plasmapheresis and conventional treatment.^179^

### 5.5.3. Acute Pancreatitis Due to Other Etiologies

#### Question 5.5.3.1: How should the alcohol cessation support program be for patients with acute alcoholic pancreatitis?

**Recommendation 5.5.3.1:**

- A brief alcohol intervention is recommended to prevent an acute alcoholic pancreatitis attack. (Level of Evidence: 1A, Strength of recommendation: Weak consensus (76.2%)).

***Comment** **:*** Excessive alcohol consumption not only leads to significant mortality and morbidity but also causes social problems. To reduce heavy drinking, brief advice or brief counseling provided by doctors and nurses can be important.^180^ Brief interventions include feedback on risky alcohol use and health-related harms, identification of high-risk situations for heavy drinking, simple advice on reducing intake, strategies to increase motivation for behavior change, and the development of a personal plan. These brief interventions typically consist of 1-5 sessions of orally delivered information, advice, or counseling, designed to last 5-15 minutes with doctors and about 20-30 minutes with nurses.^181^

According to a meta-analysis of 22 RCTs involving 7619 participants, which did not include patients with alcoholic pancreatitis, counseling for alcohol cessation is important in preventing attacks of alcoholic pancreatitis. Participants who received brief interventions consumed less alcohol over a follow-up period of 1 year or longer compared to the control group that only received assessments. Additionally, longer interventions did not result in a significant reduction in alcohol consumption compared to brief interventions.^182^ Given the numerous studies conducted since the 2007 Cochrane review, an update was performed in 2017. This update included 69 studies randomizing a total of 33 642 participants, allowing for new subgroup analyses. The primary meta-analysis, which included 34 studies, provided moderate-quality evidence that participants who received brief interventions consumed less alcohol 1 year later compared to those who received minimal or no intervention.^180^

Another meta-analysis involving 22 RCTs suggested that multi-session brief interventions may be particularly beneficial in reducing alcohol consumption among non-dependent patients. However, due to the lack of quantitative analysis, additional evidence is needed to reach more robust conclusions.^183^

In another RCT, patients presenting to the hospital with alcohol-related AP were randomized to receive either repeated interventions or only an initial intervention against alcohol consumption. The group receiving repeated interventions, which included follow-up visits at outpatient clinics every 6 months over a period of 2 years, showed a reduction in the recurrence of AP compared to the group that only received the initial intervention during hospitalization. This resulted in a decrease in hospitalization rates.^184^

#### Question 5.5.3.2: Can the same drug be used again in patients with drug-related AP?

**Recommendation 5.5.3.2:**

- The suspected drug should not be reused in cases of drug-related AP. However, if the drug is absolutely necessary for the disease, it may be used with close monitoring and dose reduction. (Level of Evidence: 3, Strength of recommendation: Weak consensus (75.2%)).

***Comment** **:*** Although drug-induced AP is rare, identifying a drug as the cause of AP presents a challenge for clinicians.^185^ Most of the available data come from case reports or case-control studies. If the benefits of the drug causing AP outweigh its risks or the potential for another severe AP attack, the drug may be reused.^186^ While the exact cause of drug-induced pancreatic damage is unknown, it can be categorized into those drugs with dose-dependent intrinsic toxicity and those causing damage through idiosyncratic reactions in the host.^187^

A comprehensive analysis of 1060 cases of drug-induced AP observed that most drugs causing severe AP were administered to treat significant pathologies, cancers, and autoimmune diseases. The more severe the disease, the higher doses of the offending drugs were used, leading to severe AP. In this analysis, when the problematic drug was re-administered at a reduced dose, it led to less severe outcomes. If reuse of the drug is necessary, close monitoring of the patients and administering a reduced dose of the drug are recommended.^188^ Another study analyzing 250 cases of drug-induced pancreatitis suggested that if the diagnosis of drug-induced pancreatitis is highly suspicious, the patient significantly benefits from the responsible drug, and there are no alternative medications to treat the serious disease, the drug may be cautiously reintroduced despite the risks.^184^

#### Question 5.6: What are the indications for referral to a tertiary center and ICU admission in AP patients?

**Recommendation 5.6:**

- Patients with a BISAP score of 3 or higher at diagnosis and those experiencing moderate or severe attacks according to the revised Atlanta criteria during follow-up should be promptly referred to a tertiary center. (Level of Evidence: 1A, Strength of recommendation: Strong consensus (87.2%)).
- Patients with confirmed or strongly suspected biliary etiology (those considered for ERCP and/or cholecystectomy) should be referred to specialized centers. (Level of Evidence: 2B, Strength of recommendation: Strong consensus (87.2%)).
- Acute pancreatitis has a rapidly changing prognosis and should be closely monitored, especially within the first 48 hours. (Level of Evidence: 1A, Strength of recommendation: Strong consensus (88.4%)).
- Patients with persistent organ dysfunction should be monitored in an ICU. (Level of Evidence: 1A, Strength of recommendation: Strong consensus (87.7%)).

***Comment** **:*** It is known that the course of AP can change rapidly, especially with treatment during the first 48 hour.^66,190,191^ Therefore, patients diagnosed with AP should be closely monitored in the initial hours, and necessary treatments should be promptly administered. Additionally, a study on the treatment of AP within the first 72 hours has demonstrated the impact of early intervention on the prognosis of the disease.^192^ Risk assessments should be conducted at the time of diagnosis to determine the disease prognosis. Patients with moderate or severe AP should be quickly referred to tertiary hospitals due to the need for intensive care.^66,190^ The BISAP and revised Atlanta criteria are recommended scoring systems in this context.^191^

In a comprehensive cohort study involving 889 468 patients, it was found that the average referral time for patients with severe AP was 4 days. The study noted a significantly higher incidence of biliary etiologies among the referred patients, and it was demonstrated that referring AP patients from hospitals without biliary intervention capabilities had a significant impact on mortality.^193^

Persistant organ dysfunction and persistent organ failure are life-threatening conditions resulting from the systemic response of AP. According to the revised Atlanta criteria, this group of patients, classified as severe, is recommended to be monitored in ICUs. This approach has been shown to reduce mortality.^66,190,194,195^

### 6. Complications and Management

#### Local and Peripancreatic Complications:

#### Question 6.1: What are the local complications of AP?

**Recommendation 6.1:**

- Peripancreatic fluid collections (acute peripancreatic fluid collection (APFC), PP, acute necrotic collection (ANC), walled-off necrosis (WON)), abdominal compartment syndrome, gastric outlet obstruction, biliary obstruction, splenic and portal vein thrombosis, colonic necrosis, solid organ involvement, pancreatico-pleural fistula, and pancreatic ascites. (Level of evidence: 1A, Strength of recommendation: Strong consensus (100%)).

**Supplementary Figure 1.**
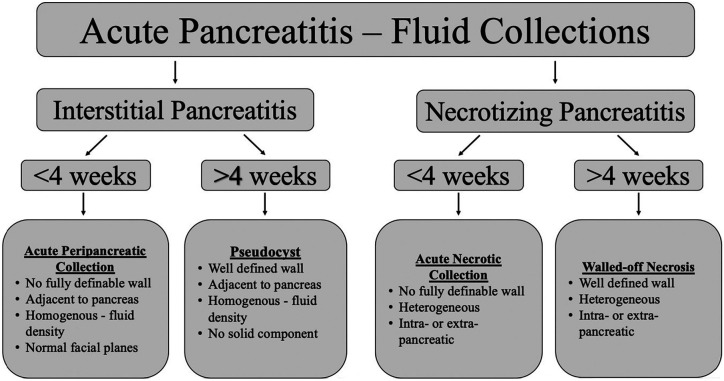
Atlanta Classification of Pancreatic Fluid Collections.

***Comment** **:*** Local complications of AP, including peripancreatic fluid collections, are classified into 4 categories according to the Revised Atlanta classification: APFC, PP, ANC, and WON (Supplementary Figure 1).^65^ Acute peripancreatic fluid collection and PPs occur in cases of interstitial pancreatitis, whereas ANC and WON arise in cases of necrotizing pancreatitis. Each of these can be either sterile or infected.

**APFC:** Acute peripancreatic fluid collection refers to fluid collections that accumulate around the pancreas in interstitial edematous pancreatitis without peripancreatic necrosis. This term describes peripancreatic fluid areas that appear within the first 4 weeks after the onset of interstitial edematous pancreatitis, containing no solid material and lacking a defined wall.^196^ On CT, APFCs are seen as homogeneous collections of fluid density located adjacent to the pancreas, within the retroperitoneum, and along the normal peripancreatic fascia surface, without a well-defined wall. Most acute fluid collections remain sterile and typically resolve spontaneously without intervention.^197^

**PP:** Pancreatic pseudocysts, which typically require 4 weeks to mature, refer to well-defined collections of homogeneous fluid, devoid of solid material, that emerge no earlier than 4 weeks after the onset of acute interstitial edematous pancreatitis. They are usually located outside the pancreas, although they can occasionally be partially or entirely intrapancreatic.^198-200^ On CT scans, they appear as well-circumscribed, usually round or oval collections with homogeneous fluid density. Although CT is the most commonly used imaging modality to identify PPs, MRI or TAUS may be necessary to confirm the absence of solid content in the collection.^63^

Pancreatic pseudocysts typically originate from the rupture of the main pancreatic duct or smaller peripheral side branches, or secondary to local edema from pancreatic/peripancreatic inflammation. Therefore, the fluid aspirated from these cysts shows high amylase levels.^201-203^ Pancreatic pseudocysts can develop in 5-15% of patients with AP.^204,205^

**ANC:** These are collections that appear in the first 4 weeks following the onset of necrotizing pancreatitis, containing variable amounts of fluid and necrotic tissue, and lacking a well-defined wall.^65^ They occur in 5-10% of patients with AP.^67,206,207^ The necrosis can involve the pancreatic parenchyma and/or peripancreatic tissues.^208^ Peripancreatic necrosis is typically characterized by heterogeneous, ill-defined areas located in the retroperitoneum and the omental bursa.^209^ On CT scans, they appear as non-enhancing, focal or diffuse areas without a surrounding wall.^210^

Acute necrotic collection can be associated with the rupture of the main pancreatic duct within areas of parenchymal necrosis. These collections may be either infected or sterile. To confirm the presence of solid content in the collection, imaging modalities such as MRI, TAUS, or EUS can be used.^65,211^ Magnetic resonance imaging and EUS are superior to CT in evaluating necrotic material within the collection.^212^

In the acute phase, it can be challenging to distinguish between APFC and ANC. Although CT is the current standard imaging technique for AP, it cannot exclude the presence of necrotizing pancreatitis at the time of initial presentation or within the first 48-72 hours.^213,214^ To determine the presence or absence of necrosis, contrast-enhanced CT or MRI should be performed at least 72 hours after the onset of symptoms.^196,215^

**WON:** Walled-off necrosis typically develops about 4 weeks after the onset of necrotizing pancreatitis and is characterized by a well-defined inflammatory wall encasing a collection of pancreatic and/or peripancreatic necrotic tissue.^65^ Liquefaction of necrotic tissue begins approximately 2-6 weeks after the onset of acute necrotizing pancreatitis, resulting in a collection that contains both fluid and solid material, clearly delineated by a distinct boundary.^216^ Therefore, the term WON refers to a mature ANC distinguished by a well-defined, thickened wall between necrotic and viable pancreatic tissue. Walled-off necrosis can be either sterile or infected and may occur in single or multiple locations. On CT, WON appears heterogenous, containing fluid and solid areas of varying degrees of loculation, and is surrounded by a well-defined, non-enhancing wall. It can be located within or outside the pancreas.^217^ However, CT may not be able to clearly differentiate the fluid and solid components, necessitating the use of MRI, TAUS, or EUS for more precise evaluation.

According to the revised Atlanta classification, WON typically develops 4 weeks or more after the onset of AP. However, a multicenter study found that 43% of the walled collections developed within the first 3 weeks after the onset of necrotizing pancreatitis.^216^

Among the other local complications of AP are abdominal compartment syndrome, gastric outlet dysfunction, biliary obstruction, vascular complications (pseudoaneurysms, splenic and portal vein thrombosis, etc.), involvement of other organs (colon necrosis, splenic infarction, etc.), pancreaticopleural fistula, and pancreatic ascites.^65,210,218^ Local complications should be suspected if abdominal pain persists or recurs, if there are increases in serum pancreatic enzyme activity, ongoing organ dysfunction, or if symptoms of sepsis such as fever and leukocytosis develop.

The development of fistulas between the pancreas and other organs is a rare complication. Fistulas can be classified as either external or internal. External fistulas are more common and often develop secondary to therapeutic drainage or surgical procedures. Internal fistulas, which are less frequently observed, can occur between the pancreas and various organs such as the colon, pericardium, and pleura. Pleuropancreatic fistulas, a very rare complication, are seen in 0.4-4.5% of pancreatitis cases.^218^ These fistulas are more frequently observed in CP, in males, and in alcohol-related pancreatitis.^219^ A distinct laboratory finding is elevated amylase levels in the pleural fluid.

Inflammatory exudates and peripancreatic collections can extend across fascial planes, affecting adjacent solid organs such as the liver, spleen, and kidneys. Splenic involvement is the most common.^197^ In most patients, Gerota’s fascia serves as a protective barrier against the enzymatic and inflammatory effects of pancreatic fluid. However, in rare cases, pancreatic fluid can breach the fascial planes, incorporating both kidneys into the inflammatory process.^220^

### Question 6.2: How should PP management be conducted?

**Recommendation 6.2:**

- Pancreatic pseudocysts should be managed conservatively unless symptomatic. Indications for drainage include cyst infection, persistent intra-abdominal symptoms (abdominal pain, nausea, vomiting, early satiety), gastric outlet obstruction, and biliary obstruction with accompanying jaundice. (Level of Evidence: 3, Strength of recommendation: Strong Consensus (96.8%)).
- Endoscopic drainage should be the preferred approach for draining PPs adjacent to the stomach or duodenum due to its less invasive nature and high clinical success rates. Surgical drainage may be considered for patients in whom endoscopic intervention fails and/or is anatomically unsuitable.
- Percutaneous drainage can be preferred for cysts inaccessible via endoscopy, or for patients with comorbidities precluding endoscopy or surgery. (Level of Evidence: 1A, Strength of recommendation: Strong Consensus (91.9%)).

***Comment** **:*** The incidence of PP in AP varies between 5% and 16%.^221^ Some PPs can spontaneously regress and do not require any intervention.^222,223^ In a prospective multicenter study, PP developed in 19 (14.7%) of 129 patients with pancreatic fluid collections (mean diameter 9.7 ± 5.3 cm). During follow-up, the cyst resolved in 5 (26.3%) patients, reduced in size in 11 (57.9%) patients, and complications related to infection developed in 2 (10.5%) patients.^224^ In a retrospective study of 75 patients, 48% were followed with conservative treatment, and complete resolution was observed in 60% of the conservatively treated PPs, while the size remained stable or decreased in 40%. In this study, surgery was required in 67% of cysts larger than 6 cm and in 40% of those smaller than 6 cm due to persistent abdominal pain, PP enlargement, or complications.^225^ However, in another study where 36 PP patients were followed with conservative treatment, 61% required intervention due to persistent pain, gastric outlet obstruction, jaundice, and weight loss, while 39% continued follow-up. Cyst sizes were found to be similar in both the conservative treatment and intervention group.^226^ Nguyen et al^227^ reported that PP clinical outcomes were similar regardless of size (greater than or less than 6 cm), whereas Rasch et al^228^ treated 34% (44/129) of PP patients conservatively and reported that even cysts up to 160 mm could spontaneously regress. Therefore, PPs should be managed conservatively unless symptomatic. The previously accepted approach of treating PPs larger than 6 cm and unresolved within 6 weeks is no longer valid.^229-232^ Although the size of the PP alone is not an indication for drainage, it is generally known that cysts larger than 6 cm have a higher likelihood of being symptomatic or causing complications.^223,230^. Intervention is necessary when PPs are symptomatic, with indications for drainage including cyst infection, persistent intra-abdominal symptoms (abdominal pain, nausea, vomiting, early satiety), rapid increase in cyst size, weight loss, gastric outlet obstruction, and biliary obstruction with associated jaundice.^230,233^ If the lesion is stable, the intervention should be delayed as much as possible, and maturation of the wall should be awaited.^229^ Intervention should not be delayed in severe complications, such as infection, hemorrhage into the cyst, cyst rupture, and gastroduodenal and/or splenic artery erosions.^230^

When selecting the treatment approach for PP, considerations should include the size and location of the cyst, dilation of the main pancreatic duct and its relation to the cyst, pancreatic pathology, and the patient’s symptoms.^234^ There are 3 different strategies for draining PPs: percutaneous drainage, endoscopic drainage (both transmural and transpapillary), and surgical drainage (both open surgery and laparoscopy).

Percutaneous drainage has been widely used in the treatment of PP for a long time.^235^ However, advancements in endoscopic techniques in recent years have made endoscopic drainage the preferred method. Symptomatic PP percutaneous drainage has similar success rates to endoscopic drainage but is associated with higher re-intervention rates, the presence of an external drain for an extended period, longer hospital stays, and a significant risk of post-procedural percutaneous fistula development.^236-239^ In a prospective study by Wan et al^240^, clinical success rates for endoscopic and percutaneous drainage were found to be 97.4% and 85%, respectively. The re-intervention rate (2.6% vs. 35%) and the incidence of adverse events (33.3% vs. 65%) were lower in the endoscopic group. A recent meta-analysis found that re-intervention (OR: 0.19; 95% CI: 0.08-0.45) and the need for surgical intervention (OR: 0.08; 95% CI: 0.02-0.39) were significantly lower in the endoscopy group compared to the percutaneous drainage group, with a shorter overall hospital stay in the endoscopy group (standard mean difference −0.60; 95% CI: −0.84 to −0.36). Clinical success, recurrence, adverse events, and mortality were found to be similar between the 2 groups.^241^ In a population-based study comparing percutaneous and surgical drainage, involving 7060 patients, the hospital stay after laparoscopic surgery was shorter than that for percutaneous drainage, with higher risks of acute kidney failure, urinary tract infections, sepsis, and acute respiratory failure identified in the percutaneous group.^242^ Another population-based study involving 14 914 patients found that the hospital stay was shorter for open surgical procedures (15 ± 15 days) compared to percutaneous procedures (21 ± 22 days), with lower inpatient mortality (2.8% vs. 5.9%) and fewer complications. Additionally, the percutaneous approach increased the likelihood of inpatient mortality by 1.37 times compared to surgery (95% CI: 1.12-1.68).^243^ Therefore, percutaneous drainage should be preferred for cysts that cannot be accessed endoscopically or for patients with comorbidities that preclude endoscopic or surgical intervention.

Most studies evaluating surgical and endoscopic interventions have shown similar treatment success, adverse event rates, and the need for re-intervention for both approaches.^229,244,245^ However, endoscopic treatment has been associated with shorter hospital stays, better physical and mental health outcomes for patients, and lower cost.^244-247^ In a systematic review and meta-analysis conducted by Zhao et al^247^, surgical drainage was reported to have higher clinical success rates compared to the endoscopic group (OR: 0.43; 95% CI: 0.20-0.95; *P* = .04). In contrast, a systematic review and meta-analysis by Farias et al^248^ demonstrated no significant difference in treatment success rates and drainage-related adverse events between surgical and endoscopic drainage. Hospital stay duration (risk difference (RD): −4.23; 95% CI: (−5.18, −3.29); *P* < .00001) and treatment costs (RD: −4.68; 95% CI: (−5.43,−3.94); *P* < .00001) were better in the endoscopic group. Another systematic review found no difference in adverse events between endoscopy and surgical drainage, although the hospital stay was longer in the surgical group.^249^

In conclusion, due to its less invasive nature and high clinical success rates, endoscopic drainage should be the preferred approach for draining PPs adjacent to the stomach or duodenum. Endoscopic ultrasonography-guided PP drainage has become a standard and safe procedure in many centers. Surgical or percutaneous drainage may be considered for patients in whom endoscopic intervention fails or is anatomically unsuitable.

### Question 6.3: In patients with PP for whom endoscopic drainage is indicated, which method (conventional vs. EUS) should be preferred?

**Recommendation 6.3:**

- In patients with luminal compression, both conventional and EUS-guided drainage have similar technical success and complication rates. The choice should be based on the clinic’s expertise.
- In cases of PP without luminal compression, in patients with coagulopathy, in the presence of cyst-adjacent vascular structures, and when complications arise during conventional procedures, EUS-guided drainage is specifically recommended (Level of Evidence: 1A, Strength of recommendation: Strong consensus (93.6%)).

***Comment** **:*** In patients with PP for whom a decision for transmural endoscopic drainage has been made, the procedure can be performed using either conventional methods or EUS. Theoretically, performing the drainage procedure using the conventional method is done blindly and may increase the risk of complications such as bleeding and perforation. The use of EUS, in this context, can make the procedure safer by identifying intervening vascular structures and showing the distance between the cyst and the tract. Additionally, the higher resolution of EUS compared to other imaging methods may lead to the diagnosis of alternative conditions in patients diagnosed with PP and subsequently alter the treatment plan.^250,251^

The outcomes of transmural drainage (TMD) using EUS and conventional methods have been compared in 2 prospective studies (n = 53 and n = 99).^250,252^ In these studies, EUS drainage was used for patients without luminal compression, those with gastric varices or coagulopathy, those where the conventional method failed, or those who experienced bleeding during the procedure. In other patients, the conventional method was used. The results showed that, 1-1.5 months post-procedure, the treatment success rates of EUS and the conventional method (90% vs. 95.2% and 93% vs. 94%, respectively) and the incidence of complications (4.3% vs. 3.3% and 19.5% vs. 18.8%, respectively) were similar across both studies. However, in 2 more recent RCTs (n = 30 and n = 60), the technical success of EUS (100% and 94%) was found to be significantly higher than that of the conventional method (33.3% and 72%).^253,254^ No significant differences were found between the groups regarding complications (EUS: 0% vs. conventional: 13.3%; EUS: 7% vs. conventional: 10%). However, when cases with luminal compression were separately evaluated in these studies, the technical success of the conventional method increased to 83.3% (5/6) and 100% (20/20). It was reported that the need for re-intervention was significantly higher in those who underwent conventional drainage (18/44; 40.9%) compared to those who underwent EUS drainage (2/45; 4.4%) (OR: 11.1).^249^ Among a total of 283 patients (173 EUS and 110 conventional) undergoing endoscopic drainage in prospective and randomized studies, there were 2 procedure-related mortalities, both associated with early and late bleeding after conventional drainage.^253,255^

### Question 6.4: Should transpapillary drainage be added to transmural drainage in the treatment of PP?

**Recommendation 6.4:**

- Routine addition of transpapillary drainage to TMD in the treatment of PP is not recommended.
- Transpapillary drainage may be considered when there is a connection between PP and the main pancreatic duct and when TMD is unsuccessful. (Level of Evidence: 3, Strength of recommendation: Strong consensus (93.6%)).

***Comment** **:*** The endoscopic drainage of PP can be performed using TMD (transgastric, transduodenal), TPD, or a combination of both methods.^205,256^ Transpapillary drainage involves placing a stent in the pancreatic duct to ensure ductal continuity and the physiological flow of pancreatic fluid into the duodenum, thereby preventing fluid leakage into the cyst.^257,258^ Generally, TPD can be performed when the size of the PP is less than 6 cm and the cyst is associated with the main pancreatic duct.^259,260^ Transpapillary drainage is also applicable in the presence of a stricture or leak in the pancreatic duct, when TMD is not feasible or contraindicated due to a distance greater than 1 cm from the enteric lumen or conditions such as coagulopathy.^232,259,261^ The proximal end of the stent can be placed directly into the PP or used to bridge the area of ductal leakage.^262^

Although it is thought that adding TPD to the TMD procedure via EUS in patients with PP may facilitate recovery, there is insufficient evidence to suggest that it increases treatment success. Early studies supported this hypothesis by reporting better outcomes with combined transmural and transpapillary drainage compared to TMD alone.^263^ In a retrospective study of 110 patients undergoing PP drainage (62% TMD, 48.5% combined drainage), treatment success was higher in those who received a pancreatic stent at the site of the ductal leak compared to those who did not (97.5% vs. 80%; *P* = .01). It was reported that placing a pancreatic stent to bridge the leak in patients undergoing TMD had a positive impact on treatment outcomes.^263^ However, the benefit seemed to be limited to patients with partial pancreatic duct damage. In a study by Shrode et al^264^, among 47 patients with PP and partial pancreatic duct damage, resolution rates were 75% with TPD and 78% with combined drainage. However, for complete pancreatic duct disruptions, adding TPD to the treatment did not provide an additional benefit compared to transmural or percutaneous drainage alone (52.9% vs. 70.6%; *P* = .61). The authors reported successful outcomes in PP resolution with TPD, particularly when there was partial disruption of the pancreatic duct and the stent bridged the leak.^265^

In contrast to these studies, other studies have not demonstrated that adding TPD to TMD improves treatment success (239,264,266). Hookey et al^239^ conducted endoscopic PP drainage in 116 patients (15 transpapillary, 60 transmural, and 41 combined drainage) and reported no significant difference in clinical success rates between those who underwent TMD alone or combined drainage (90.6% and 82.9%, respectively). However, a higher recurrence rate was observed with the combined approach (26.8%) compared to TMD alone (8.3%) (*P* = .015). The authors suggested that adding TPD to TMD could potentially hinder the patency and maturation of the cystoenterostomy fistula, thereby limiting the resolution of PPs. However, this study included a heterogeneous population comprising PPs, acute necrotic collections, and abscesses.

In a more recent multicenter study comprising a homogeneous patient population (PPs), 174 PPs undergoing EUS-guided TMD, with 95 (55%) receiving TMD alone and 79 (45%) undergoing combined drainage, showed no difference in PP resolution rates (TMD, 69%; combined drainage, 62%; *P* = .61) or complication rates between groups. However, in multivariate analysis, the TPD procedure was found to be negatively associated with long-term PP resolution.^266^ A larger study involving 211 patients demonstrated no additional benefit of combined TPD over EUS-guided TMD alone (adjusted OR: 1.70; 95% CI: 0.56-5.14; *P* = .34). In this study, successful placement of a pancreatic stent bridging the leak was reported in 40% of PPs.^267^ Barthet et al^255^ prospectively evaluated 50 PP patients, with 28 patients undergoing EUS-TMD, 13 conventional endoscopic TMD, and 8 conventional endoscopic TPD. The overall technical success rate was 98% (49/50), clinical success was achieved in 90% of cases, and no significant difference was observed among the 3 groups.

In a meta-analysis evaluating whether combined drainage provides additional benefits compared to TMD alone, 9 studies (2 prospective, 7 retrospective) encompassing 604 drainage procedures (373 TMD and 231 combined drainage) were assessed. Combined drainage did not show additional benefits in terms of technical success (OR: 1.12; 95% CI: 0.37-3.37; *P* = .85), clinical success (OR: 1.11; 95% CI: 0.65-1.89; *P* = .70), recurrence (OR: 1.49; 95% CI: 0.53-4.21; *P* = .45), or complications (OR: 1.15; 95% CI: 0.61-2.18; *P* = .67) compared to TMD alone.^268^

In most of the included studies, the drainage method was determined based on the endoscopist’s preference. If a leak was detected in the pancreatic duct via ERCP, TPD was performed. As there are no randomized prospective studies evaluating the benefit of combined drainage, the data are primarily derived from retrospective observational studies. Additionally, although the collections included in these studies were predominantly PP, the groups were still heterogeneous, and data to classify endoscopic treatment outcomes according to the type of collection were not available. This heterogeneity makes it challenging to compare the results of the studies.

In conclusion, the lack of definitively improved treatment outcomes with the combined drainage approach, the relatively low technical success rate of TPD, and the well-known potential side effects associated with ERCP suggest that routine TPD is not recommended for PPs. In the endoscopic treatment of PPs, TPD may be performed if there is a leak or partial rupture in the pancreatic duct, or if the pancreatic duct is associated with the cyst.

### Question 6.5: How is the diagnosis of infected necrosis made?

**Recommendation 6.5:**

- Diagnosing infected necrosis can be challenging. Clinical findings (such as newly developed fever, SIRS, and organ failure), laboratory tests, and imaging methods are used for diagnosis. (Level of Evidence: 3, Strength of recommendation: Strong consensus (94.9%)).
- Progressive increases in CRP and especially PCT levels assist in making the diagnosis. (Level of Evidence: 1B, Strength of recommendation: Strong consensus (95.2%)).
- On CT, while the sensitivity of detecting extraluminal air within the necrotic area is low, its specificity is high and valuable for diagnosis. (Level of Evidence: 3, Strength of recommendation: Strong consensus (94.3%)).
- In cases where a decision cannot be made based on clinical, laboratory, and imaging findings, fine-needle aspiration (FNA) can be performed. (Level of Evidence: 3, Strength of recommendation: Strong consensus (91.4%)).

***Comment** **:*** Diagnosing infected necrosis can be challenging. In patients with necrotizing pancreatitis, suspicion of infected pancreatic necrosis arises when there is clinical deterioration (new or persistent fever, SIRS, organ failure). Diagnosis involves utilizing clinical findings, laboratory tests, and imaging methods. In a retrospective surgical series of 208 patients, clinical findings alone diagnosed infected necrosis in 80% of the patients, which increased to 94% when CT was included.^269^ Although the sensitivity of detecting extraluminal gas within the necrotic area on CT is low (27.5-60%), its specificity is high (81.5-100%) and valuable for diagnosis.^269-271^ A small study (n = 20) demonstrated that diffusion MRI could detect infection in APFC with 100% sensitivity and 90.9% specificity.^271^ In another study involving cases of APFC, PP, and WON (n = 40), diffusion MRI showed a sensitivity of 67-75% and a specificity of 96% for detecting infection.^272^

Progressive increases in CRP and PCT levels can guide the prediction of infected necrosis in severe AP. A meta-analysis published in 2014 found that CRP had a sensitivity of 64%, specificity of 82%, and a positive likelihood ratio of 3.34 for detecting infected necrosis, whereas PCT had a sensitivity of 85%, specificity of 78%, and a positive likelihood ratio of 4.54.^273^ Procalcitonin was identified as the best test for predicting infected necrosis, with a higher positive likelihood ratio in patients with necrotizing pancreatitis compared to all patients with pancreatitis (9.3 vs. 4.5).^273^ However, it is important to keep in mind that PCT is a non-specific marker for infectious complications. Before diagnosing infected necrosis, other infection foci should be carefully investigated. In another study included in the same meta-analysis, CRP (cut-off: 430 mg/L) was shown to predict MODS or death associated with infected necrosis with 50% sensitivity and 99% specificity, while PCT (cut-off: 3.5 ng/mL) had 90% sensitivity and 89% specificity for the same outcomes.^274^

Fine-needle aspiration is a safe method, and studies (n = 30-115) have shown that it can distinguish between sterile necrosis and infected necrosis with a sensitivity of 76.4-84% and a specificity of 85-100%.^275-277^ Given that delaying intervention until the necrosis wall forms in patients with infected necrosis who are clinically stable reduces mortality and morbidity, it can be said that an early diagnosis of infection does not change the treatment approach. Additionally, while the positive predictive value of FNA ranges from 86-100%, it has been reported to have a false-negative rate of 25-50%.^269,275,278^ For these reasons, it is recommended that FNA should not be routinely performed in patients suspected of having infected necrosis but should be considered in cases where clinical, laboratory, and imaging findings are inconclusive.

### Question 6.6: What is the optimal timing for intervention in infected necrosis?

**Recommendation 6.6:**

- After the diagnosis of infected pancreatic necrosis, patients should be closely monitored under appropriate antibiotic and nutritional support, if necessary, in intensive care settings.
- In patients who do not respond to conventional treatment, waiting at least 4 weeks before invasive interventions is a more suitable approach in terms of potential complications.
- However, if the patient’s clinical condition deteriorates with signs of persistent organ failure, minimal invasive intervention should be considered irrespective of time. (Level of Evidence: 1B, Strength of recommendation: Strong consensus (91.4%)).

***Comment** **:*** Pancreatic necrosis is a life-threatening local complication of AP. When it becomes infected or causes obstructive symptoms, it often necessitates invasive intervention. Guidelines recommend delaying invasive intervention for at least 4 weeks, if possible, to allow for encapsulation of the collections. The primary rationale for delaying drainage is that encapsulated necrosis is more amenable to intervention and the procedure tends to have fewer complications. Moreover, studies have shown that some patients with infected necrotizing pancreatitis can recover within the first 4 weeks with antibiotic therapy alone. There is a demonstrated linear relationship between early open necrosectomy and high mortality, with the intent being to protect these critically ill patients from the “additional harm” of early-stage open surgery. However, this evidence is primarily based on studies involving surgical interventions from earlier periods.^279,280^

With the use of minimally invasive intervention methods and changes in standard treatment, the necessity of delaying intervention until wall formation, especially in patients without persistent organ failure, has come under discussion. The goal here is to control the source of infection without removing the infected necrosis, reduce SIRS, prevent sepsis, and improve the patient’s clinical condition. Therefore, if there is clinical deterioration despite maximum medical support, earlier intervention may be considered even in the absence of encapsulation.^229,281^ This approach has been increasingly accepted by specialist physicians dealing more intensively with pancreatitis, and percutaneous interventions in suitable cases have become part of the conservative treatment concept, in addition to the medical and nutritional support provided to patients.^282^

In the only RCT conducted to date regarding the timing of intervention for infected necrosis, no differences were found between the early intervention group and the delayed intervention group in terms of major complications, mortality, or length of hospital and ICU stays. In the delayed intervention group, 35% of patients were treated with antibiotics alone, and this group required fewer invasive interventions.^279^ Additionally, a recent meta-analysis, which includes 5 retrospective studies and the aforementioned RCT, indicated that early minimally invasive intervention (<4 weeks) does not increase hospital mortality. However, compared to delayed intervention, it is associated with a significantly longer hospital stay and an increased incidence of gastrointestinal fistula or perforation.^283^ Outside of this meta-analysis, other studies, mostly retrospective, found no general differences in complications, intensive care, or length of hospital stay.^284-286^ Nevertheless, some studies have reported higher rates of persistent organ failure and the need for re-intervention in the early intervention group.^281,287,288^

Guidelines published by various groups regarding the timing of intervention for infected necrosis similarly recommend delaying intervention, if possible, for 4 weeks. However, they suggest considering earlier intervention in the presence of an organized collection and strong indications.^214^.

### Question 6.7: How should asymptomatic WON be managed?

**Recommendation 6.7:**

- Sterile asymptomatic WONs do not require invasive intervention regardless of their size or location. Approximately 45-65% remain asymptomatic during follow-up.
- Complications in asymptomatic WON generally develop within the first 6 months. Close monitoring of patients is recommended during this period.
- During follow-up, spontaneous fistulization to the stomach, duodenum, jejunum, and colon may occur. Fistulization to the colon requires surgery. (Level of evidence: 3, Strength of recommendation: Strong consensus (94.3%)).

***Comment** **:*** Necrotic pancreatic tissues can remain solid or liquefy, and they can remain sterile or become infected. The current literature contains only a limited number of articles addressing the natural course of asymptomatic WON.^289-292^ It is known that more than half of sterile asymptomatic WONs remain asymptomatic during follow-up, with at least half of these resolving spontaneously. A large proportion of those that do not resolve spontaneously may continue to remain asymptomatic.^289^ There are no available parameters to predict which WONs may develop complications, and if complications occur, they typically do so within the first 6-7 months. The most common complications are infection or pain in the sterile WON. Hence, close monitoring of lesions during this period is necessary.^289-291^ Another potential complication during follow-up is spontaneous fistulization to the gastrointestinal tract.^293^ The only study in the literature reports an 11.5% rate of spontaneous fistulization of asymptomatic WON to the gastrointestinal tract. Fistulization most commonly occurs to the stomach, but cases involving the duodenum, jejunum, and esophagus have also been reported. Endoscopic intervention through the fistula tract can be performed in these regions. However, fistulization to the colon requires urgent surgical intervention.^293^

In conclusion, sterile asymptomatic WONs do not require invasive intervention regardless of their size or location. A “watch and wait” strategy appears to be appropriate for these lesions.^289-293^

### Question 6.8: What are the optimal treatment methods for symptomatic WON?

**Recommendation 6.8:**

- Endoscopic drainage treatment for WON has lower fistula formation, shorter hospital stays, and lower re-intervention rates compared to various surgical and percutaneous methods.
- For patients with collections unsuitable for endoscopic drainage, the preferred approach may be percutaneous drainage or minimally invasive surgery.
- Percutaneous drainage can provide short-term benefits to stabilize some patients and may prevent further interventions in one-third of the cases.
- Patients with WON that extends into the paracolic gutters or pelvis may require percutaneous drainage in addition to the endoscopic procedure.
- Minimally invasive surgical options, such as video-assisted retroperitoneal debridement (VARD) and laparoscopic transgastric debridement, can be directly used or in conjunction with endoscopic and transgastric interventions in experienced centers for locations where these interventions are not suitable.

Due to the heterogeneous nature of WON, it is important to personalize treatment and perform it in experienced centers.

(Level of Evidence: 1A, Strength of recommendation: Strong consensus (100%)).

***Comment** **:*** For patients with symptomatic WON who do not respond to medical treatment, a drainage procedure (with or without necrosectomy) is indicated. The methods include an endoscopic approach, a percutaneous approach, and surgical necrosectomy. Surgical options comprise open surgery and minimally invasive surgery. Minimally invasive surgery includes VARD and laparoscopic transgastric debridement.

The results of the Minimally invasive ‘step-up approach’ versus maximal necrosectomy in patients with acute necrotising pancreatitis (PANTER trial), published by the Dutch Pancreatitis Study Group in 2010, demonstrated that a minimally invasive step-up approach reduced mortality and major complications from 69% to 40% compared to primary open necrosectomy. This finding led to the abandonment of open necrosectomy.^294^ In the Transluminal Endoscopic Step-Up Approach versus Minimally Invasive Surgical Step-Up Approach in Patients with Infected Pancreatic Necrosis (TENSION) trial conducted by the same group, the clinical comparison results between minimally invasive and endoscopic step-up approaches showed no difference in terms of mortality and major complications. However, the length of hospital stay and pancreatic fistula rates were lower in the endoscopic step-up treatment group.^295^ The Minimally Invasive Surgery Versus Endoscopy Randomized (MISER) Trial demonstrated that endoscopic step-up treatment significantly reduced the systemic inflammatory response compared to minimally invasive surgery. Following this study, endoscopic TMD methods began to be recommended as the forefront option.^296^

Endoscopic drainage procedures (with or without necrosectomy) can be performed transgastrically or transduodenally, depending on the location of the WON.^214^ Numerous meta-analyses have demonstrated that TMD procedures, compared to various surgical and percutaneous methods, are associated with lower fistula formation, shorter hospital stays, and reduced rates of reintervention.^241,297^

Percutaneous drainage can provide rapid and effective source control in patients who are too asthenic for endoscopic or surgical drainage. Prospective studies and systematic reviews have shown that primary percutaneous drainage alone can eliminate the need for further surgical intervention in 35-56% of patients with WON.^67,294,298^ Percutaneous drainage has a definite advantage when lesions are located in the paracolic gutters or the pelvis, areas that might be inaccessible to endoscopic drainag.^229^ Additionally, as part of a step-up approach, it can pave the way for minimally invasive surgical procedures such as VARD.

One of the most significant disadvantages of percutaneous drainage is the high incidence of pancreatic fistulas. The incidence can be as high as 32% with percutaneous drainage, compared to just 2% with the endoscopic approach.^295^ Considering the pancreatic fistulas observed during surgical step-up therapy and percutaneous drainage, as well as the stent-related complications during endoscopic step-up therapy, it is important to recognize that WON is a heterogeneous disease. Therefore, the treatment should be individualized and carried out in experienced centers.

### Question 6.9: How is “Disconnected Pancreatic Duct Syndrome (DPDS)” defined?

**Recommendation 6.9:**

- It is the disruption of the integrity of the main pancreatic duct, resulting in the complete disconnection of its 2 ends. (Level of Evidence: 1A, Strength of recommendation: Strong consensus (97.4%)).

***Comment** **:*** Total disruption of the main pancreatic duct is a significant complication of acute necrotizing pancreatitis and can also occur, albeit more rarely, after CP and trauma. While it can develop anywhere along the pancreatic duct, it most frequently occurs in the head-neck region of the pancreas. A viable, functioning segment of the pancreas remains in the tail, with its secretions contributing to peripancreatic fluid collections. This condition is referred to as disconnected pancreatic duct syndrome (DPDS). Due to both extraductal leakage causing damage to the viable pancreatic tissue and the secretion flow from the distal severed pancreatic tissue into the retroperitoneum, this situation leads to the formation of pancreatic and peripancreatic necrosis or pseudocysts.^299-301^

### Question 6.10: What are the treatment methods for DPDS?

**Recommendation 6.10:**

- A step-up approach may be recommended:

- Conservative

- Minimally invasive approach (endoscopic ± percutaneous)

- Surgical approaches

- In endoscopic treatment, long-term TMD with plastic stents is sufficient for most patients. Depending on the characteristics of the collection, initial application of metal stents and direct endoscopic necrosectomy (DEN) may be performed.
- Transmural stents should be maintained for a long period. Before removal, imaging techniques (preferably secretin-enhanced MRCP) should confirm the absence of a pancreatic duct ‘feeding’ the cyst. (Level of Evidence: 3, Strength of recommendation: Strong consensus (88.5%)).

***Comment** **:*** There is currently no standardized treatment method accepted for DPDS. Understanding the natural course of the disease is essential for determining the appropriate treatment approach. Observations reveal that in these patients, the pancreatic duct feeding the peripancreatic fluid collection spontaneously closes over time. This results in a pancreatic tissue with dilated ducts at the tail that eventually atrophies, while the pancreatic tissue in the head region continues to drain into the duodenum. This closure process takes several months.^302^ The treatment goals during this period are the drainage of the fluid collection and, if necessary, the performance of necrosectomy. Drainage and necrosectomy procedures can be performed using endoscopic, percutaneous, or surgical methods, or a combination of these approaches.

There are 2 main surgical methods for treating DPDS: distal pancreatectomy and Roux-en-Y internal drainage. The drainage procedures can be performed in 3 ways: cystojejunostomy/gastrostomy, fistulojejunostomy/gastrostomy, and pancreaticojejunostomy/gastrostomy.^303^ Although surgical methods have traditionally been used in DPDS, advances in endoscopic tools, accessories, and techniques have made endoscopic treatments less invasive alternatives. Among these methods are TPD, TMD, and DEN.^304^ Initially, the success rates of endoscopic treatments were reported to be lower.^299,305^ However, with the advent of EUS-guided interventions and the concept of long-term stenting, the success rates have increased, as these approaches allow for the intervention of cysts that are adjacent to but not compressing the tract.^306^ Metal stents, specifically lumen apposing metal stents (LAMS), are initially placed, but because long-term use of metal stents is associated with increased complications (e.g., bleeding, embedding), they are replaced with double pigtail plastic stents when long-term stenting is needed.^307,308^ These stents are maintained in place until spontaneous closure occurs. Percutaneous drainage in these patients is generally not considered suitable due to patient comfort concerns.

In a meta-analysis comprising 35 studies and including a total of 1355 patients, the success rates of endoscopic and surgical treatments were examined. The complete success rate of endoscopic treatment (defined as the resolution of pancreatic fluid collection without recurrence) was reported at 82%. However, this rate was lower in studies that involved only TPD (58.5%) and higher in patients who underwent TMD (90.6%). The success rate for combined drainage in this meta-analysis was found to be 64.6%, whereas the complete surgical treatment success rate was reported at 84.7%. The success rates of surgical and endoscopic treatments were found to be comparable. Specifically, the success rate for distal pancreatectomy (86.8%) was similar to that of drainage surgeries (86.3%).^309^ Another meta-analysis that investigated surgical treatment methods found that patients who underwent distal pancreatectomy experienced higher intraoperative blood loss and developed more endocrine and exocrine insufficiencies compared to other approaches.^310^

The only scenario in which TPD is theoretically expected to be effective is in accelerating the healing of a proximal pancreatic leak. By definition, in patients with DPDS, a guidewire will not pass to the tail side, and it has been shown that placing a stent up to the level of the leak does not provide any additional benefit to the treatment’s success. Conversely, the risk of the cyst becoming infected with duodenal contents increases.^266,268^

There is no specific, comparative study, or consensus on the number of plastic stents and their duration in patients with DPDS. Traditionally, transmural plastic stents are removed after 6-8 weeks. However, in patients with DPDS, the recurrence rate is high because the pancreatic leak feeding the cyst often persists during this period. One retrospective study reported this rate to be 42%.^311^ It has been shown that this risk is high within the first year.^312^ Therefore, long-term retention of plastic stents is recommended. According to the limited studies available, durations of up to 2-6 years have been reported.^311-315^ During follow-up, the absence of leakage into the collection area or the dilation of the previously non-dilated duct of the isolated pancreas, as shown by secretin-enhanced MRCP, may indicate the closure of the leak.^316^

Transmural stents can migrate or fragment during long-term follow-up. While these complications can be asymptomatic and detected incidentally, they can also lead to more serious issues such as recurrent pancreatic fluid collection (PFC), bowel perforation, obstruction, and PFC infection. A meta-analysis reported this complication rate as 8.5%.^309^ In such cases, repeat endoscopic or surgical treatments may be necessary.

With the increased success of endoscopic treatment, a step-up approach may be recommended for the management of DPDS: conservative therapies, minimally invasive approaches, and surgical approaches. Given the advancements and high success rates in the endoscopic treatment repertoire today, there is a decreasing need for surgical intervention.

### Question 6.11: How should splanchnic venous thrombosis be managed in patients with AP?

**Recommendation 6.1.11:**

- In patients with moderate to severe APs, dynamic imaging should be utilized to assess for the presence of splanchnic venous thrombosis (SVT). (Level of Evidence: 3, Strength of recommendation: Strong consensus (93.6%)).
- If isolated splenic vein thrombosis is present, the thrombus extends to the mesenteric vein, or there is a portal vein thrombosis without collateral formation at the time of detection and anticoagulant use is not contraindicated, anticoagulant therapy should be administered with careful consideration of bleeding risk, particularly in patients with pseudocysts. (Level of Evidence: 3, Strength of recommendation: Strong consensus (97%)).
- In patients starting anticoagulation therapy without an underlying thrombophilic disorder, the treatment duration should be 3-6 months. (Level of Evidence: 3, Strength of recommendation: Strong consensus (96%)).
- In patients with severe AP where no contraindications exist, short-term (7-14 days) prophylactic low-molecular-weight heparin (LMWH) therapy has beneficial effects on hospital stay, organ failure, and mortality. (Level of Evidence: 1A, Strength of recommendation: Strong consensus (95.1%)).

***Comment** **:*** The incidence of SVT during the course of AP is reported to be between 6% and 23%.^317^ Thrombosis, which develops due to various local and systemic factors, is most frequently observed in the splenic vein. Depending on the venous system affected by thrombosis, it can lead to potentially life-threatening complications such as hypersplenism, left-sided portal hypertension (gastric varices), and bowel ischemia.^318,319^

Splanchnic venous thrombosis related to AP is often asymptomatic and is usually detected incidentally through imaging. Existing studies that guide the management of SVT focus mainly on patients with persistent thrombotic risk. Consequently, there are no clear recommendations for managing thrombosis arising during AP. The necessity for invasive procedures (e.g., drainage and necrosectomy) that pose a bleeding risk in AP patients complicates the formulation of definitive treatment guidelines for thrombosis.^320^

The risk of developing SVT is considered high in the presence of severe disease and local complications during the course of AP.^321^ Therefore, SVT should be kept in mind when imaging is performed to investigate local complications, especially in patients with severe AP.^322^ For patients diagnosed with SVT, a gastroscopy should be conducted to assess for potential esophageal and/or gastric varices.^323^

There is no unanimous consensus on the administration of anticoagulant therapy when SVT is detected during the course of AP. The prognosis of splenic vein thrombosis, which is the most commonly encountered type in AP, is generally good. It is often thought that these cases can be monitored without anticoagulant therapy, especially when there is no underlying thrombophilic condition. Additionally, anticoagulant therapy may increase the risk of hemorrhage within pancreatic necrosis-related collections in this patient group. However, studies on this topic are heterogeneous, mostly retrospective, and generally have low levels of evidence.^324^ Indeed, a meta-analysis found no significant differences in terms of recanalization, variceal development, bleeding, and mortality between the groups that received anticoagulant therapy and those that did not.^320^ Conversely, another meta-analysis of 18 studies indicated that the group receiving anticoagulant therapy had a higher recanalization rate (OR: 0.51, 95% CI: 0.31 to 0.83, *P* = .007). No significant difference was found between the 2 groups concerning bleeding and mortality.^317^ Therefore, anticoagulant therapy should be administered unless there are contraindications.

Evidence regarding the anticoagulant treatment approach is insufficient in patients with portal and/or mesenteric vein thrombosis, outside of the splenic vein. Portal vein thrombosis is considered an indication of serious disease due to the potential risk of liver dysfunction, and anticoagulant treatment is predominantly recommended.^325^ In patients with thrombosis extending to the mesenteric vein, anticoagulant treatment should also be administered, especially in the presence of clinical signs of intestinal ischemia.^323^

In the management of thrombosis in SVT with an AP course, the duration of anticoagulant therapy should not exceed 3-6 months. Patients with severe complications of portal hypertension should be carefully evaluated, and the type of anticoagulation to be used should be selected on an individual basis.^326^

Heparin can improve the prognosis of AP by reducing the release of cytokines and inflammatory mediators, inhibiting the inflammatory cascade, and preventing microthrombosis.^327^ For this reason, there are numerous studies on the prophylactic use of LMWH, particularly in patients with severe AP. A recent meta-analysis evaluating a total of 16 studies, the majority of which included RCTs and investigated the use of LMWH for 7-14 days, found that in patients with severe AP, LMWH use was associated with a shorter hospital stay (mean difference (95% CI) −8.79 (−11.18, −6.40), *P* < .01); lower mortality (pooled risk ratio (RR) (95% CI) 0.33 (0.24-0.44), *P* < .01); lower incidence of organ failure (RR (95% CI) 0.34 (0.23-0.52), *P* < .01); lower incidence of PP development (RR (95% CI) 0.49 (0.27-0.900, *P* < .02); and less need for surgery (RR (95% CI) 0.39 (0.31-0.50), *P* < .01).^328^ These results support the prophylactic use of short-term LMWH in patients with severe AP, provided there are no contraindications.

### 7. Recurrent Acute Pancreatitis

#### Question 7.1: What is recurrent acute pancreatitis?

**Recommendation 7.1:**

- Recurrent acute pancreatitis (RAP) is defined as the occurrence of at least 2 documented episodes of AP with a remission period of more than 3 months between them, during which there is complete recovery without evidence of CP. (Level of Evidence: 1A, Strength of recommendation: Strong consensus (100%)).

***Comment** **:*** There is no consensus on the definition of RAP. Recurrent acute pancreatitis is generally defined as 2 or more well-documented episodes of AP occurring at least 3 months apart, during which there is complete recovery without signs of CP or persistent fluid collections associated with AP. It is crucial to know the interval between episodes to accurately diagnose RAP because the effects of a previous AP episode can last up to 3 months. Typically, within the first 3 months, parenchymal changes related to AP subside, although local complications like fluid collections may persist. If a patient experiences an increase in pancreatic enzymes and abdominal pain within the first 3 months following an initial AP episode, this should be considered a complication of the first AP episode rather than RAP. Therefore, before diagnosing RAP, imaging studies must be used to rule out complications that may arise after the first AP episode, such as PP, WON, hemorrhage into a cyst, portal vein or splenic vein thrombosis, bile duct compression, or gastric outlet obstruction.

In the course of RAP, the pancreas usually shows edematous changes without structural damage, although underlying CP may sometimes be detected in the first or subsequent attacks. AP, RAP, and CP can sequentially evolve from one to another due to multiple risk factors. The first episode of AP is termed the sentinel AP attack, and it is crucial for clinicians to assess the patient’s risk of developing RAP or CP during this period. If the etiology of the sentinel AP attack is not adequately identified and eliminated, there is a high likelihood that the patient will develop RAP in the future. Similarly, early recognition of RAP and its etiology is important to prevent further attacks and the progression to CP.^329-333^

Idiopathic recurrent acute pancreatitis (IRAP) is defined as the inability to determine the etiology of RAP despite a detailed patient history, routine laboratory tests (including liver function tests, serum calcium, and triglyceride levels), genetic mutation testing, and imaging methods (such as CT, MRI, MRCP, or EUS).^329^

#### Question 7.2: In what proportion of AP patients does RAP develop?

**Recommendation 7.2:**

- 10-30% of patients presenting with AP develop RAP. (Level of Evidence: 2A, Strength of recommendation: Strong consensus (90.4%)).

***Comment** **:*** In a retrospective cross-sectional study conducted in the Netherlands involving patients with AP, it was reported that 17% of these patients developed RAP and 7.6% developed CP. Both necrotizing pancreatitis and smoking were found to be independent risk factors for the development of RAP and CP.^334^ In another study with a median follow-up of 40 months after the first AP episode, 22% of patients were diagnosed with recurrent AP. Additionally, 6% of the patients were diagnosed with CP.^335^ In a different study with an average follow-up of 7.8 years, 16.5% of the patients developed RAP.^332^ A meta-analysis reported the frequency of IRAP among patients with AP to be 29.4%.^336^ In a study conducted in China, 10.7% of patients presenting with AP experienced a recurrence. Of these, 7.1% had only one recurrence, 1.9% had 2 recurrences, and 1.7% had more than 2 recurrences. Multivariate analysis identified male gender, alcohol- and hypertriglyceridemia-associated etiology, and the presence of local complications at the initial presentation as factors associated with RAP.^337^

#### Question 7.3: Is there a difference in etiology between single and recurrent attacks?

**Recommendation 7.3:**

- Biliary causes are predominant in single attacks of AP, whereas alcohol and HTG are more prominent in RAP. (Level of Evidence: 2A, Strength of recommendation: Strong consensus (91.4%)).

***Comment** **:*** In cases of a single AP attack, the etiology was found to be 41% biliary, 21% alcohol-related, 26% idiopathic, and 13% other causes. In patients with RAP, alcohol use, male gender, and smoking were identified as risk factors.^338^ Another study investigating the severity and recurrence of AP based on etiology found that alcohol-related AP had a higher recurrence rate compared to biliary AP (OR: 2.98 (CI: 2.22–4.01)).^22^

#### Question 7.4: Which patients are at risk for recurrent attacks after the initial sentinel episode?

**Recommendation 7.4:**

- Smoking, male gender, high TG levels, genetic factors, and local complications during the first attack increase the risk of RAP. Additionally, continuing alcohol consumption after alcohol-induced AP and not performing cholecystectomy after biliary AP are conditions that increase the risk of RAP. (Level of Evidence: 2B, Strength of recommendation: Strong consensus (98.9%)).

***Comment** **:*** Studies monitoring the natural course of patients after AP have identified alcohol use, smoking, and the absence of cholecystectomy after biliary pancreatitis as risk factors for RAP.^335,339-341^ In a prospective study involving 20 centers and 460 RAP patients, alcohol and smoking were determined to be independent risk factors for the development of RAP (OR: 1.91; *P* = .01).^342^ Another prospective study with 15 centers involving 669 AP patients found that 117 (17%) developed RAP. The highest cumulative risk for RAP was observed in smokers at 40%.^334^ A prospective study in the United States that evaluated a multi-ethnic cohort also identified smoking as a risk factor for RAP in both women and men.^343^ In a retrospective study by Munigala et al^344^ involving 6799 AP patients, those who developed RAP were analyzed, and smoking was identified as a risk factor. Various retrospective studies have similarly found that the risk of recurrent attacks is higher among smokers. These studies also identified male gender, local complications, and alcohol use as risk factors for RAP.^338,345,346^ Another study highlighting the increased risk of RAP among smokers also showed that quitting smoking reduces this risk. This underscores the importance of recommending smoking cessation.^347^

To identify the risk factors leading to recurrence, a study involving 56 patients with recurrent pancreatitis found that male gender and local complications at the first attack were the strongest risk factors. Alcohol and HTG were identified as other risk factors.^337^ Similarly, a study by Cho et al^348^ concluded that male gender and local complications increased the risk of recurrence. Another study involving 167 patients with recurrent attacks identified TG level and BMI as 2 independent predictive factors for recurrence, with thresholds of 5.9 mmol/L for TG and 28.2 for BMI.^9^ Vipperla et al^349^ observed a 32% recurrence rate in patients with alcohol use and uncontrolled high triglycerides. A retrospective cohort study demonstrated that in patients with HTG, a decrease in TG levels reduced the risk of pancreatitis.^350^ Another study identified TG levels exceeding 3000 mg/dL or failure to maintain TG levels below 500 mg/dL as strong and independent risk factors for RAP.^351^ These findings underscore the importance of early diagnosis and treatment of HTG to reduce the risk of RAP.

Another factor that increases the risk of RAP is the failure to perform cholecystectomy following the first biliary AP attack.^352,353^ A retrospective study of 17 010 patients with biliary AP showed that 78% of the patients underwent cholecystectomy within the first 4 weeks as per guidelines, and those who had the procedure exhibited a significantly lower rate of RAP compared to those who did not (3% vs. 13%, *P* < .001).^354^ In a prospective study, 226 AP patients and 66 RAP patients were followed for an average of 42 months. The recurrence rate for untreated biliary pancreatitis patients was 31.3%, compared to 18% for those treated with ERCP, 16% for those who underwent cholecystectomy, and 0% for those who received both treatments. Patients who had cholecystectomy showed a significant increase in recurrence-free survival and a notable reduction in recurrence risk (hazard ratio (HR) = 0.45). Among alcoholic AP patients, those who abstained from alcohol had a reduced recurrence rate (5.8% vs. 33%; *P* = .05).^355^ This finding was corroborated by another similar study.^356^

Genetic factors play a significant role in the development of RAP. In a study conducted by Gürakar et al^357^, genetic testing was performed on 59 patients with IRAP, and CFTR, transheterozygous variants, SPINK1, and chymotrypsin C (CTRC) variants were detected in 66% of the patients. The combination of genetic factors with smoking and alcohol consumption was reported as the most important risk factors for RAP. A 2019 study demonstrated that patients with AP who had SPINK1 mutations developed RAP more quickly.^358^ In another study comparing 87 patients with RAP to those who had a sentinel attack and healthy individuals, the frequencies of the PRSS1 p.R122H mutation, SPINK1 p.N34S variant, and PRSS3 p.E32del variant were found to be higher in the RAP group.^359^ Additionally, a study showed that while the SPINK1 N34S polymorphism did not increase the risk of a sentinel attack, it did increase the risk of recurrent attacks (OR: 19.1, 95% CI: 2.4-149.6).^360^

**7.5. What are the Treatment Options in RAP?**

#### Question 7.5.1: What are the medical treatments in RAP (autoimmune pancreatitis (AIP) treatment, hyperlipidemia treatment, antioxidants, ursodeoxycholic acid (UDCA), pancreatic enzymes)

**Recommendation 7.5.1:**

Identification and treatment of the underlying etiological factor to reduce the number of attacks in RAP is recommended. However, there is insufficient evidence that specific treatments can reduce or prevent the number of RAP attacks.

(Level of Evidence: 2A, Strength of recommendation: Strong consensus (95.1%)).

***Comment** **:*** The frequency of RAP after the first AP attack is between 17-30%.^361^ There is no high-quality data suggesting that identifying the etiology of acute attacks can prevent further AP attacks. Medical treatments targeting specific etiologies are partially effective in preventing RAP. Recurrent acute pancreatitis can develop due to biliary causes, alcohol, hypertriglyceridemia, AIP, post-ERCP, hypercalcemia, drugs, or idiopathic reasons.^330^ There are treatment options targeting the underlying etiology. The use of steroids and immunosuppressants in AIP and the control of serum triglyceride levels in HTG-AP are believed to prevent relapses. Although various treatments, including antioxidants, UDCA, and pancreatic enzymes, have been used in patients with RAP, there is currently no convincing high-quality evidence supporting the preventive efficacy of medical treatments for RAP. The 2018 International Consensus on Recurrent Acute Pancreatitis also reported that there is no proven role of preventive medical treatment in the management of RAP.^330^

In most RAP patients where no organic cause is identified through laboratory and standard imaging methods (TAUS, CT, MRCP), the etiology is often occult biliary microlithiasis, sludge, or sphincter of Oddi dysfunction (SOD).^362,363^ For patients with acute biliary pancreatitis who cannot undergo cholecystectomy due to high surgical risk, endoscopic sphincterotomy (EST) is an alternative option.^364^

In a study involving patients with biliary etiology, where UDCA was administered to prevent relapse, a relapse rate of 38% was reported at the end of a 1-year follow-up.^330^ In patients with IRAP, endoscopic biliary sphincterotomy with or without the administration of UDCA (12 mg/kg) has been used; however, it has not been proven to definitively prevent RAP.^361^

Autoimmune pancreatitis is a type of CP known to be part of IgG4-related disease. Type 2 AIP is defined as idiopathic duct-centric pancreatitis and should be considered in the differential diagnosis of RAP.^365^

In medical treatment, studies on octreotide, a somatostatin analogue, have shown that it reduces the severity of AP, prevents post-ERCP pancreatitis and postoperative pancreatic fistulas, and reduces pain in CP. However, there is no data indicating that it prevents RAP attacks.^330^

The efficacy of pancreatic enzyme therapy in preventing attacks has been evaluated in 2 small retrospective studies. In one of these studies, patients were given enzyme therapy for 2.5 years, and it was shown that one-third of the cases did not develop attacks, while in 65% of the cases, attacks were reduced by 50%.^330^ In another retrospective study, patients were grouped into acute and CP, but RAP was not specified in the sub-analysis. These studies are very small in number and are still at the abstract stage, thus the evidence value is low. Therefore, the recommendation for adult and pediatric patients with RAP is that pancreatic enzyme supplements should not be used unless there is pancreatic exocrine insufficiency, due to the lack of sufficient data supporting their effectiveness in preventing relapse.^330,366-368^ Prospective studies are needed.

In patients with CP, plasma levels of selenium, vitamin A, vitamin E, ß-carotene, xanthine, and lycopene were found to be significantly lower compared to the control group and patients with RAP (*P* < .05).^368^ In a meta-analysis by Gooshe et al, which evaluated the use of antioxidants (vitamins C, E, A, NAC, glutamine, ß-carotene, selenium, arginine, S-adenosylmethionine (SAMe), combined preparations, allopurinol, pentoxifylline) in AP, CP, and post-ERCP pancreatitis, there were some supportive data suggesting improvements in hospital stay duration, mortality rate, reduction in complications and organ dysfunction, increased serum antioxidant levels with treatment, and decreased inflammatory biomarkers; however, the results were noted to be controversial, and no data were provided regarding the prevention of recurrence.^369^ Another study in 28 patients with IRAP, RAP, and alcoholic CP who received antioxidant preparations containing selenium, ß-carotene, vitamin C, vitamin E, and methionine for 2-6 months reported a reduction in attack frequency.^370^ In a series of 179 patients, of whom only 5 had RAP, it was reported that those who used antioxidants and received placebo had no attacks compared to the placebo group.^330^

There is no clear supporting evidence that antioxidants prevent AP relapse. However, due to their existing positive immune nutrition effects and their status as safe agents, they are used in clinical practice for patients with RAP, CP, and EPI.^330,366,368,369,371^ Consequently, there are no high-quality studies demonstrating the benefits of medical treatment in RAP.

#### Question 7.5.2: What are the endoscopic treatment methods for patients with RAP and what is their impact on the course of RAP?

**Recommendation 7.5.2:**

- In biliary RAP patients who cannot undergo cholecystectomy due to high surgical risk, or in post-cholecystectomy patients with biliary RAP, biliary endoscopic sphincterotomy (BES) may prevent new attacks (Level of Evidence: 2A, Strength of recommendation: Strong Consensus (98.6%)).
- In RAP patients associated with PD without CP findings, minor papilla endoscopic sphincterotomy (miPES) may prevent the development of new attacks (Level of Evidence: 1A, Strength of recommendation: Strong Consensus (100%)).
- Endoscopic sphincterotomy is recommended in Type I SOD and particularly in Type II SOD with enzyme elevation (Level of Evidence: 2A, Strength of recommendation: Strong Consensus (98.9%)).
- In cases of IRAP, although sufficient evidence is lacking, BES may be considered after investigating microlithiasis or other potential etiologies on a per-patient basis. Pancreatic endoscopic sphincterotomy (PES) is not routinely recommended (Level of Evidence: 2A, Strength of recommendation: Strong Consensus (97.9%)).

***Comment** **:*** In the prevention of recurrence in biliary pancreatitis, the first choice is cholecystectomy, whose efficacy is undisputed.^372^ Cholecystectomy should be performed at the earliest possible period after the resolution of the AP attack.^373^ However, for patients with a history of gallstones who cannot undergo cholecystectomy due to high surgical risk, or for those whose biliary pancreatitis attacks persist post-cholecystectomy, BES has been reported to be effective in reducing recurrences.^374,375^ In a study involving 233 patients with biliary pancreatitis, it was reported that the risk of recurrence increased 31-fold if cholecystectomy was not performed after the first attack. In patients who did not undergo cholecystectomy and also did not receive BES, recurrences were more frequent (37% versus 0%, *P* = .019).^376^ In a retrospective study evaluating 1119 patients out of 5754 who had experienced biliary pancreatitis and did not undergo cholecystectomy, the risk of RAP was 8.2% in those who received BES compared to 17.1% in the group that did not receive it (*P* < .001).^375^ In patients with RAP who are not eligible for cholecystectomy, the recurrence rate was found to be lower in the BES group compared to the control group (1.8% vs. 23%). Studies have reported that BES may reduce the risk of biliary pancreatitis in patients who cannot undergo cholecystectomy or who are on the waiting list.^377,378^ For patients with a history of cholecystectomy who experience recurrent pancreatitis attacks with symptoms suggesting a biliary origin, BES may be preferred even if stones or sludge are not detected. In up to 4-24% of patients up to 5 years post-cholecystectomy, calculi have been found in the common bile duct. Cholecystectomy significantly reduces micro-crystals and sludge but may not completely eliminate them. Sludge may also form in cases of SOD that cause permanent or temporary bile flow obstruction. Biliary endoscopic sphincterotomy is the only effective treatment in these patients.^379^

Pancreas divisum is the most common congenital anomaly (10%) arising from the failure of the ventral and dorsal embryonic pancreatic ducts to fuse during organogenesis.^380^ Pancreas divisum is reported to be more frequent in patients with RAP. Its prevalence in patients with idiopathic AP can be as high as 25.6%.^333,381,382^ It is suggested that the obstruction in patients with PD, due to stenosis or dysfunction of the minor papilla sphincter, is responsible for pain and the development of CP. Based on this pathophysiology, endoscopic or surgical ductal decompression of the minor papilla is considered to potentially treat IRAP and PD-associated chronic pain.^383^ Endoscopic treatment includes miPES, stenting, and/or balloon dilatation.^384^ Post-ERCP pancreatitis occurs in 5-15% of these cases, while the frequency of sphincter restenosis is approximately 20-30%.^383,385^ Minor papillotomy is the preferred treatment option in PD patients because stent replacement requires multiple interventions and can cause long-term changes in the dorsal duct. However, the risk of post-ERCP pancreatitis is higher with minor papillotomy compared to BES. To reduce this risk, short-term prophylactic small-caliber (3-F or 4-F) stents and rectal indomethacin may be preferred.^385^ Restenosis after sphincterotomy is a primary cause of recurrent pancreatitis after endotherapy. Therefore, short-term stenting of the dorsal pancreatic duct after sphincterotomy and stent revision if needed are recommended to prevent cicatricial strictures and post-ERCP pancreatitis.^386^

However, whether miPES can prevent pancreatitis attacks or progression to CP in these patients remains controversial.^387^ Minor papilla endoscopic sphincterotomy is recommended if the dorsal duct is dilated or if there are indications of impaired pancreatic fluid flow in the secretin test. Sphincterotomy is not indicated in patients without dorsal duct dilatation or those with normal function tests because an obstructive cause cannot be documented. In these patients, 3-month dorsal duct stenting can be performed to identify which patients could benefit from sphincterotomy by detecting unrecognized minor papillary disorders.^387^ Even though endotherapy may be effective on symptoms in patients with RAP associated with PD, whether it prevents progression to CP remains unresolved. Despite successful miPES reducing intraductal pressure, its sole effect on preventing progression to CP is still debatable.

Studies on PD have reported that endotherapy is effective on RAP and its symptoms in 60-100% of patients. In a meta-analysis that included 23 studies evaluating the efficacy of endoscopic treatment in these patients, the success rate for RAP was reported as 76%, for CP as 52.4%, and for pancreatic pain as 48%. In this study, the incidence of post-ERCP pancreatitis was 10.1%.^389^ Another meta-analysis reported a response rate to miPES of 43-100%, with a post-ERCP pancreatitis rate of 18%.^383^ In an RCT involving 19 cases, 9 of the 10 patients (90%) who received endoscopic treatment had no attacks for 3 years.^390^ A prospective multicenter pilot study (FRAMES) showed that miPES and temporary placement of a small-caliber stent significantly reduced recurrence and pain over a 6-month follow-up period.^391^ In a prospective study comparing endotherapy and conservative treatment in RAP patients without CP symptoms, with follow-up lasting up to 5 years, endoscopic ductal drainage was successful in 73.7% of cases, regardless of dorsal duct dilation. Long-term pancreatic duct stenting has been noted to pose a significant issue, particularly in cases without ductal dilation, triggering stent-associated ductal changes similar to those seen in CP. In this study, dorsal duct stenting was performed in patients receiving miPES to prevent post-ERCP pancreatitis. Short-term stenting was defined as the placement of a 7-F, 3-4 cm plastic pancreatic stent for less than 1 month, while long-term stenting was defined as the placement of a 7-F, 3-7 cm plastic pancreatic stent for up to 1 year, replaced every 3 months. The development of ductal changes similar to stent-induced CP was reported in patients who received long-term stents. Additionally, during follow-up, patients who underwent miPES with short-term stenting or without stents showed fewer findings suggestive of CP compared to those with long-term stents (33.3% vs. 80%). However, when considering only patients with successful long-term stent placement, the rate of CP development in untreated patients (64.3%) was similar (60%). These results suggest that unknown factors, aside from underlying ductal abnormalities, may also play a role in the progression of chronic disease in these patients.^392^ Given that dilation and stenting in PD patients increase the risk of iatrogenic pancreatitis and the need for repeat procedures, miPES should be considered initially.^393^

Sphincter of Oddi dysfunction can be observed in the biliary or pancreatic segment or both. SOD has been reported in 35-65% of patients with RAP.^331,394-396^ However, it remains unclear whether SOD is the initial cause of pancreatitis in RAP patients or a result of recurrent attacks. Specifically, Types 1 and 2 SOD have been reported to be associated with IRAP.^397,398^ In patients with SOD, BES can be used alone or in combination with PES. PES increases the risk of post-ERCP pancreatitis. Generally, BES is preferred in these patients. Success rates with BES are reported to be 83-100% for Type 1 SOD and 80% for Type 2 SOD. If BES fails, PES can be added.^399,400^

Endoscopic sphincterotomy is the standard treatment for Type 1 and Type 2 SOD. If the diagnosis of SOD is uncertain, EST should not be routinely recommended due to both its uncertain outcomes and the high risk of post-ERCP pancreatitis. Generally, BES is performed first and provides clinical improvement in approximately 80% of cases; PES is applied in cases of failure.^401^ Although BES alone has been reported to reduce pancreatic sphincter pressure in patients with pancreatic SOD, studies have also used dual (pancreatic and biliary) endoscopic sphincterotomy (DES) to reduce sphincter pressure.^402,403^ When BES fails, up to 78% of cases have reported elevated pancreatic sphincter pressure, and symptomatic improvement has been achieved in 60-90% of these cases with PES.^399^ In a prospective RCT comparing the efficacy of BES and DES in the treatment of pancreatic SOD, no difference was found in reducing RAP attacks between BES and DES (48.5% vs. 47.2%).^403^ Similar results were obtained in another study.^404^

After routine laboratory and imaging examinations fail to identify an underlying cause, 10-30% of RAP patients are diagnosed with IRAP. Once potential causes such as microlithiasis and SOD are excluded, the patients classified as true IRAP have limited evidence on the impact of endoscopic treatments on disease progression. Studies involving these patients report various endoscopic interventions, including BES, DES, and pancreatic stenting. Among these, pancreatic EST with or without stenting appears to be the most prominent method. However, the long-term effects and natural course of endoscopic treatments on IRAP patients remain unclear. Most studies are retrospective, small-scale, and lack control groups.^384,400,405,406^

In a cohort study involving a 7-year follow-up of IRAP patients (NAPS-2), the rates of pancreatitis recurrence and progression were found to be similar between patient groups receiving BES and those following conservative management.^405^ A prospective RCT demonstrated that in IRAP patients with normal Oddi sphincter manometry, neither BES nor DES was beneficial in preventing RAP.^403^ In another prospective RCT involving IRAP patients, individuals were subjected to pancreatic stenting, which was replaced every 3 months, and were followed for 5 years. The results showed that the stent group had fewer recurrent attacks in terms of RAP incidence, although no difference was observed in pancreatic-type pain.^407^ In a prospective study by Testoni and colleagues comparing the efficacy of UDCA and BES in IRAP, BES prevented recurrences in 78.6% of patients, while UDCA did so in 75%.^400^

### 8. Long-Term Complications of AP and their Management

#### Question 8.1: Does the risk of exocrine pancreatic insufficiency (EPI) increase in AP?

**Recommendation 8.1:**

- The likelihood of EPI increases in patients who have experienced AP.
- The incidence of EPI is higher in alcoholic AP compared to biliary AP, and in severe AP compared to mild AP.
- The frequency of EPI is highest within the first year following an episode of AP.

(Level of Evidence: 1B, Strength of Recommendation: Strong Consensus (95.4%)).

***Comment** **:*** Two recent meta-analyses have indicated a potential increase in the risk of EPI among AP patients. In the meta-analysis conducted by Hollemans et al, the pooled prevalence of EPI following AP was found to be 27.1%. Subgroup analyses within this meta-analysis revealed that the risk of developing EPI was higher in cases of alcoholic AP compared to biliary AP, severe AP compared to mild AP, and necrotizing AP compared to edematous AP.^408^ In another, more recent meta-analysis, the prevalence of EPI was 24% in edematous AP and 47% in necrotizing AP. Furthermore, the risk of EPI was found to be higher in alcoholic AP compared to biliary AP (OR: 1.62, 95% CI: 1.13-2.32), and in severe and moderately severe AP compared to mild AP according to the revised Atlanta criteria (OR: 1.94, 95% CI: 1.12-3.34). This study also identified that the risk of developing EPI was highest within the first year following AP (severe AP: 58%, mild AP: 37%).^366^

#### Question 8.2: How should EPI resulting from AP be treated?

**Recommendation 8.2: Treatment of EPI resulting from AP is similar to the treatment of EPI due to other causes.**

- Pancreatic enzyme replacement therapy (PERT) should be administered to patients with AP-induced EPI. The initial dose is 40 000-50 000 units at main meals and 25 000 units at snacks. Based on treatment response, doses can be increased to a maximum of 80,000 units at main meals and half of this amount at snacks (Level of Evidence: 1B, Strength of Recommendation: Strong Consensus(92.3%)).
- A dietary plan with frequent, small-volume meals is recommended. At least 1 meal should include a normal amount of fat (Level of Evidence: 3, Strength of Recommendation: Strong Consensus (91.7%)).
- Periodic screenings for nutritional deficiencies (fat-soluble vitamins, magnesium, zinc, vitamin B12) should be conducted, and supplementation should be provided if deficiencies are detected (Level of Evidence: 1B, Strength of Recommendation: Strong Consensus (94.3%)).

***Comment** **:*** In cases of pancreatitis, PERT has been shown to improve symptoms, digestion, and overall health.^409,410^ Guidelines for administering PERT in CP or EPI are well-defined. Generally, an initial dose of 40 000-50 000 units is recommended for main meals, and 25 000 units for snacks. Depending on the treatment response, the dose can be increased to a maximum of 80 000 units for main meals and half of that for snacks. In addition to PERT, frequent meals with small portions are advised, and to prevent calorie restriction, intake of a normal amount of fat in at least 1 meal is recommended.^411-414^ Nutritional deficiencies may not be clinically apparent; therefore, upon diagnosis of EPI, levels of fat-soluble vitamins, magnesium, zinc, and vitamin B12 should be assessed, deficiencies corrected, and monitored periodically.^415^ Follow-up for EPI can be evaluated through clinical assessment, laboratory tests, and improvements in fecal elastase levels.^411,416^

#### Question 8.3: What is the definition of pancreatic ascites?

**Recommendation 8.3:**

- Pancreatic ascites is defined as intraperitoneal fluid collection that occurs during the course of AP, with a Serum-Ascites Albumin Gradient (SAAG) < 1.1 and amylase > 1000 IU/L (Level of Evidence: 3, Strength of Recommendation: Strong Consensus (92.6%)).

***Comment** **:*** Pancreatic ascites is a rare complication. It develops as a result of the fistulization of peripancreatic fluid collection into the lesser sac or directly into the peritoneum due to pancreatic duct disruption in acute necrotizing pancreatitis. It is often accompanied by pleural effusion. The peritoneal cavity accumulates a fluid rich in pancreatic proteases, unsaturated fats, hemoglobin, and pro-inflammatory cytokines. These molecules are highly toxic and lethal, also contributing to the development of intra-abdominal compartment syndrome. Therefore, pancreatic ascites is an important predictor of the severity and poor prognosis of AP.^416-418^

During the course of AP, it is necessary to differentiate pancreatic ascites from reactive ascites, chylous ascites, and ascites associated with portal hypertension. Reactive ascites typically develops early, spontaneously resorbs, and does not last longer than 1 week.^318^ Chylous ascites is characterized by its white fluid appearance and high TG levels.^419^ Portal hypertensive ascites may be caused by splanchnic thrombosis or portal hypertension associated with AP. Intraperitoneal fluids associated with AP, other than pancreatic ascites, have a transudative character and low amylase levels.^417,418^ There are no RCT scharacterizing the fluid of pancreatic ascites. In a retrospective study by Rana et al^421^, fluid amylase levels in 12 patients with pancreatic ascites following acute necrotizing pancreatitis ranged from 3600 to 96 000 IU/L; no cut-off value for fluid amylase was specified in this study. Case reports have suggested peritoneal fluid with a serum-ascites albumin gradient (SAAG) <1.1 and pancreatic amylase levels >1,000 IU/L as indicative of pancreatic ascites.^420^

#### Question 8.3.1: How should pancreatic ascites be managed?

**Recommendation 8.3.1:**

- Endoscopic treatment methods should be preferred in suitable cases. In cases of partial pancreatic duct disruption, TPD is an appropriate method. (Level of Evidence 3, Strength of Recommendation: Strong Consensus (95.4%)).
- Long-term treatment with plastic stents placed using the TMD method can be achieved in DPDS. (Level of Evidence 3, Strength of Recommendation: Strong Consensus (88.5%)).
- Percutaneous drainage can be applied in the event of increased pain, clinical deterioration, new-onset organ failure, or abdominal compartment syndrome (Level of Evidence 3, Strength of Recommendation: Strong Consensus (88%)).
- Surgery should be considered in cases where endoscopic treatments are inappropriate or unsuccessful. (Level of Evidence 3, Strength of Recommendation: Strong Consensus (90.5%)).

***Comment** **:*** Pancreatic ascites can be treated using conservative, endoscopic, and surgical methods. Before making a treatment decision, the presence of pancreatic duct disruption and/or DPDS should be evaluated. There are a limited number of studies related to the treatment of pancreatic ascites. In a retrospective study by Rana et al^421^, 12 patients who developed pancreatic ascites following acute necrotizing pancreatitis were evaluated. In 9 of these patients, pancreatic ascites was associated with PFC (3 with PP, 6 with WON), while it was isolated in 3 cases. Endoscopic ultrasonography-guided TMD was performed on the 9 patients with PFC, and both the PFC and pancreatic ascites were observed to regress within 2-3 weeks. Transmural stents were left indefinitely in the 8 patients due to the presence of DPDS. In 1 patient with partial duct disruption, a transpapillary plastic stent was placed, which was removed after the duct disruption had resolved. Among the 3 patients with isolated pancreatic ascites, 2 had partial duct disruption, and 1 had DPDS. The patients with partial duct disruption were followed with a plastic stent placed via ERCP, resulting in ascites resolution within an average of 6 weeks.^420^ Additional studies support the effectiveness of TMD in the presence of DPDS and TPD in partial duct disruption for pancreatic ascites treatment.^417,422^ Recently published case-based reports also suggest that pancreatic ascites, resulting from acute exacerbation of CP, can be effectively treated with transpapillary and transluminal endoscopic interventions.^423-428^ In conclusion, for patients with disrupted pancreatic ducts, TPD and EUS-guided interventional methods are recommended at experienced centers for those with DPDS.

There are no sufficient clinical prospective studies demonstrating that early percutaneous drainage of ascitic fluid reduces mortality or prevents the development of organ failure. In a retrospective clinical cohort study evaluating 102 patients with AP, it was reported that draining ascitic fluid via percutaneous catheter drainage or abdominal paracentesis reduced inflammatory markers and delayed or prevented advanced interventions and multiple organ failure.^428^ In a single prospective study involving 255 cases of AP, early drainage was shown not to increase mortality and complications associated with infection.^429^

Patients with pancreatic ascites tend to have a poor response to conservative treatment methods. Medically, NJ feeding is theoretically beneficial as it can reduce pancreatic secretions and contribute to the resolution of pancreatic duct disruptions.^423^ The effectiveness of octreotide has been more commonly evaluated in cases of pancreatitis secondary to trauma and CP, and there is insufficient data regarding its efficacy in acute pancreatic ascites.^430^

Surgical treatment may be considered when endoscopic therapies are inadequate. Most of the published experiences regarding the surgical treatment of pancreatic ascites involve studies conducted on patients with underlying CP, and there is limited data on the surgical treatment of pancreatic ascites in the context of AP. Surgery is a risky procedure in AP due to widespread inflammation and vascular complications.^431^

#### Question 8.4: How is a pseudoaneurysm that develops after pancreatitis defined?

**Recommendation 8.4:**

- A pseudoaneurysm may develop in the visceral arteries following AP (Level of Evidence: 1A, Strength of recommendation: Strong consensus (100%)).

***Comment** **:*** Pseudoaneurysm and other vascular complications related to PFC and PP are the result of arterial erosion caused by proteolytic enzymes secreted by the pancreas. The prevalence of developing visceral artery pseudoaneurysm (VA-PSA) after AP is 0.05%, with an incidence rate of 4-10%. VA-PSA is more frequently observed in cases of severe, necrotizing, and alcohol-induced AP. The most commonly affected arteries are the splenic artery, gastroduodenal artery, and superior mesenteric artery, which are located close to the pancreas.^432-434^

#### Question 8.4.1: What is the clinical presentation of pseudoaneurysm developing after AP?

**Recommendation 8.4.1:**

- Pseudoaneurysm should be suspected in cases of abdominal pain, a drop in hemoglobin (gastrointestinal and intra-abdominal bleeding), and sudden growth of the cystic lesion. (Level of Evidence: 1A, Strength of recommendation: Strong Consensus (98.7%)).

***Comment** **:*** Pseudoaneurysm developing after AP can be asymptomatic or present with sudden clinical deterioration, a drop in hemoglobin (GIS and intra-abdominal bleeding), and sudden enlargement of a cystic mass on imaging. It can lead to hemosuccus pancreaticus. Diagnosis is made using CT angiography (arterial phase).^435,436^

#### Question 8.4.2: What is the treatment for a pseudoaneurysm developing after AP?

**Recommendation 8.4.2:**

- Endovascular embolization (coil) is the first treatment option. If this fails, surgical treatment may be applied (Level of Evidence: 1A, Strength of recommendation: Strong Consensus (98.7%)).

***Comment** **:*** In a 2019 meta-analysis evaluating 29 studies involving patients with AP and CP, the incidence of pseudoaneurysm development in AP was found to be 0.05%, with the most frequent occurrence in the splenic artery (37.7%). The most common treatment method was coil embolization, with a technical success rate of 95% and a clinical success rate of 88% over 54 months of follow-up. There was no difference in technical and clinical success rates between patients with AP and CP; however, mortality was lower in patients with CP. In conclusion, endovascular embolization was noted to have high technical and clinical success rates.^436^

Depending on the patient’s hemodynamic status, angiography should be performed as soon as possible, and a combination of coil and embolic agents is recommended for embolization. When embolization is successful, a surveillance angiography should be performed 24-48 hours later. In cases of embolization failure or hemodynamic instability, emergency surgery should be considered. Semi-emergency surgery is defined as a procedure performed within 48 hours after angiography in patients showing intermittent bleeding in the ICU, and once their hemodynamic functions have stabilized.^437,438^

#### Question 8.5: Does Diabetes Mellitus (DM) develop during the course of AP?

**Recommendation 8.5:**

- Diabetes may develop in both the early and late periods following AP (Level of Evidence: 1B, Strength of recommendation: Strong Consensus (98.9%)).

***Comment** **:*** After AP, transient hyperglycemia may particularly develop. This transient hyperglycemia is both prognostically significant and a risk factor for the development of DM in later stages. Studies have shown that DM can develop in both the early and late periods following AP. A meta-analysis reported that among patients followed for 12-179 months after AP, the prevalence of DM was 15-24% before 60 months, but increased to 40% after 60 months.^439^ It is possible that AP triggers Type 1 DM autoantibodies in genetically predisposed individuals at risk of developing DM. Catecholamine secretion during AP can cause transient hyperglycemia. Post-AP, the development of DM may primarily involve beta cell loss, insulin resistance, and autoantibody development. The diagnostic criteria are the same as for type 2 DM. The rate of DM development is associated with the necrosis and etiology of AP. Risk factors for DM development after AP include male gender and age >40 years.^440,441^ The frequency of EPI in patients who developed DM after AP was found to be 40%. Studies have shown a higher risk of pancreatic cancer in patients who develop DM after AP.^442^

#### Question 8.5.1: How should DM be treated after AP therapy?

**Recommendation 8.5.1:**

- Metformin is effective in the treatment of DM after AP. Insulin therapy may be needed earlier compared to type 2 DM (Level of Evidence: 1B, Strength of Recommendation: Strong consensus (89.9%)).

***Comment** **:*** There is no consensus on when or who should be screened for impaired glucose metabolism after AP. It is recommended to conduct screening using fasting glucose, or HbA1c, and an oral glucose tolerance test (OGTT) at 6-month intervals. Metformin is effective in the treatment of DM after AP.^442^ Insulin therapy may be required earlier than in type 2 DM.^443^

#### Question 8.6: Does CP develop after the first AP episode?

**Recommendation 8.6:**

- A small portion of patients may develop CP after the first AP episode. Progression to CP is associated with alcohol, smoking, and pancreatic necrosis. (Level of Evidence: 1B, Strength of Recommendation: Strong consensus (93.6%)).

***Comment** **:*** After the first episode of AP, approximately 8-13% of patients progress to CP.^334,443^ In a study conducted by Nøjgaard et al, a 30-year follow-up of patients was performed, and the progression from AP to CP was found to be associated with alcohol consumption, smoking, and pancreatic necrosis. While smoking, alcohol consumption, and their combined use showed the highest cumulative risk for CP, smoking was identified as the strongest risk factor associated with progression.^334,444^

### 9. Surgery in AP

#### Question 9.1: What should the timing of cholecystectomy be in acute biliary pancreatitis (Mild) (Early vs. Late)?

**Recommendation 9.1:**

- Cholecystectomy should ideally be recommended to the patient after pancreatitis has subsided, preferably during the hospital stay and within 4 weeks if possible. (Level of Evidence: 1A, Strength of Recommendation: Strong consensus (96.1%)).

***Comment** **:*** Acute biliary pancreatitis is one of the most common gastrointestinal emergencies. Between 35% and 55% of AP cases are associated with gallstones.^445^ In a multicenter study by Köksal et al^11^, biliary etiology was identified as the most frequent cause, accounting for 67.1% of cases. While cholecystectomy is the primary treatment option for these patients, the optimal timing of the procedure, whether early or delayed, remains a topic of debate in the literature. Studies conducted in the 1980s suggested high morbidity and mortality rates for cholecystectomies performed within the first 48 hours, leading clinicians to wait until the patient’s symptoms and laboratory findings had subsided. However, with advancements in diagnostic and therapeutic methods and the growing experience with minimally invasive surgery, the validity of this approach is being questioned by numerous recent studies.^446^

Considering these studies, it is evident that the definition of early and late groups varies among studies. However, in most studies, the “time of admission” is defined as the early group.^446-450^ Additionally, some studies classify early periods as the first 24, 48, or 72 hours, as well as 1, 3, and 4 weeks.^450-458^ Conversely, the late group in these studies is taken as 2, 3, 4, 6, or 8 weeks, while in some, the late group is determined as the time when symptoms have resolved. This heterogeneity is noticeable at first glance in retrospective studies. Despite this variation, the common finding across these studies is that cholecystectomy performed in the early period—regardless of the specific time frame and even if the symptoms and laboratory findings have not yet resolved—significantly reduces the hospital stay without altering the complication rates. Similarly, most studies show no significant differences in conversion rates to open surgery and operation durations between the early and late groups, with the rates of readmission being similar or lower in the early group.^450-458^

Numerous RCTs have also been conducted on this subject. In a study conducted in 2010 (25 early vs. 25 delayed), the length of hospital stay was found to be shorter in the early group, with similar conversion and complication rates between the 2 groups.^459^ Pancreatitis of biliary origin, optimal timing of cholecystectomy (PONCHO) trial conducted in 2016 (128 early vs. 136 delayed) revealed that the readmission rates were lower in the early group, which also translated to a more favorable cost analysis for the early group.^460^ Other RCTs conducted subsequently have shown similar results.^461,462^ In an RCT carried out in 2019 focusing on patients with mild biliary pancreatitis, early cholecystectomy performed within 24 hours of admission was associated with significantly reduced ERCP rates (15% vs. 29%), time to surgery (16 hours vs. 43 hours), and hospital stay duration (50 hours vs. 77 hours).^463^ Furthermore, a cost analysis of this study later calculated that early cholecystectomy has an 81% probability of reducing the total 90-day costs.^464^

In light of all this information, considering similar complication and conversion rates, and based on the durations reported in the literature, it is deemed appropriate to perform surgery at the time of the patient’s admission or, if possible, within 4 weeks. This approach is justified by the significant reduction in hospital stay, readmission rates, recurrent biliary events, and associated costs.

#### Question 9.2: What should be the timing of cholecystectomy in acute biliary pancreatitis (Severe-Necrotizing)?

**Recommendation 9.2:**

- Delaying cholecystectomy following acute (moderate and severe) biliary pancreatitis reduces morbidity. In patients who have survived an episode of moderate to severe acute biliary pancreatitis and present with PFC, cholecystectomy should be postponed for 6-8 weeks. (Level of Evidence: 3, Strength of recommendation: Strong consensus (95.2%)).

***Comment** **:*** Although cholecystectomy at the time of admission is recommended to prevent recurrent biliary events after a mild acute biliary pancreatitis attack, post-severe pancreatitis cholecystectomy is potentially associated with an increased risk of complications. There is no high-level evidence indicating the optimal timing of cholecystectomy in patients with necrotizing biliary pancreatitis.^465^ The available information in the literature is based on the results of retrospective studies. Regarding the appropriate timing of cholecystectomy, a risk assessment between recurrent biliary events and a potentially higher risk of surgical complications should be conducted. A comparative study reported a 44% complication rate in patients with moderate/severe acute biliary pancreatitis who underwent early cholecystectomy, compared to 5.5% in the late group.^466^ Another study reported poor mortality and morbidity outcomes with cholecystectomy performed within the first 48 hours in severe pancreatitis.^467^ Another retrospective evaluation suggested that delaying the timing of cholecystectomy in severe pancreatitis is effective in reducing morbidity.^468^ It is deemed appropriate to postpone surgery until peripancreatic collections or necrosis have completely resolved or, in the case of persistent collections, at least 6-8 weeks from the onset of the disease.^469,470^ Some guidelines, without specifying a time frame, recommend that clinicians delay cholecystectomy until signs of local and/or systemic inflammation subside.^471^ The most extensive analysis on this topic comes from a retrospective study of data from 191 patients. According to this study, the optimal timing of cholecystectomy after necrotizing biliary pancreatitis, in the absence of peripancreatic collections, is 8 weeks post-discharge.^471^

#### Question 9.3: Should cholecystectomy be performed in idiopathic AP?

**Recommendations 9.3:**

- Following an episode of AP with no identifiable cause, cholecystectomy should be considered in patients suitable for surgery to reduce the risk of recurrent pancreatitis attacks. (Level of Evidence: 1A, Strength of recommendation: Strong consensus (80.1%)).
- Cholecystectomy may reduce the severity of subsequent idiopathic AP attacks. (Level of Evidence: 3, Strength of recommendation: Weak Consensus (70.8%)).

***Comment** **:*** Gallstones and alcohol are the most common causes of AP. However, in 10-30% of patients, the exact cause of pancreatitis cannot be determined, and this condition is referred to as idiopathic AP.^473^ In a multicenter study conducted by Köksal et al^11^, the incidence of idiopathic AP was found to be 12%. Microlithiasis is often blamed as a cause of idiopathic AP, and earlier studies suggested that the treatment of presumed microlithiasis and biliary sludge with cholecystectomy could prevent subsequent pancreatic attacks.^474,475^ However, recent studies have approached this relationship with skepticism.^476^ Therefore, studies examining the value of prophylactic cholecystectomy in patients with an AP attack where no stones are detected in TAUS and there is no alcohol use have emerged.

In a retrospective analysis of 527 patients, cholecystectomy was shown to potentially reduce the severity of subsequent idiopathic AP and the frequency of biliary pancreatitis.^477^ In another retrospective study, among idiopathic AP patients, the recurrence rate was found to be 19.7% (13/66) in those who underwent cholecystectomy, whereas at least one AP recurrence was observed in 42.8% (68/159) of those treated without surgery.^478^ The only RCT on this topic compared 39 operated patients with 46 non-operated patients and demonstrated that cholecystectomy effectively prevented recurrent idiopathic AP when all other possible pancreatitis etiologies were carefully excluded.^479^ A meta-analysis that analyzed this issue included the results of a total of 524 patients from 10 studies. According to the results of this meta-analysis, there was a significant reduction in recurrence rates in the group that underwent cholecystectomy (11.1% vs. 35.2%).^336^

Based on this information, it is considered appropriate to perform a cholecystectomy in patients with AP of unknown etiology defined as idiopathic, to reduce the frequency of subsequent attacks. Although cholecystectomy has also been shown to potentially reduce the severity of these attacks, the level of evidence is low.

#### Question 9.4: What are the indications for surgical treatment in AP?

**Recommendation 9.4: Indications for surgical intervention:**

- Fistulization of the peripancreatic collection to the colon, intestinal ischemia, abdominal compartment syndrome where conservative and noninvasive treatments have failed, perforation, gastric outlet obstruction, intestinal obstruction, acute necrotizing cholecystitis, and bleeding where endovascular approach has failed (Level of evidence: 3, Strength of recommendation: Strong consensus (96.3%)).

***Comment** **:*** The surgical indications for AP are mostly limited to situations requiring emergency surgery and cases where other treatments have failed to address AP complications. At this juncture, the surgeon’s role in patient evaluation becomes critically important. Continuous involvement of the surgeon from the initial stages of clinical management, with the authority to decide on surgical intervention when necessary, is vital for the patient’s prognosis. In some instances, immediate surgical intervention may also be required.^480-482^

#### Question 9.5: What should be the timing of surgical intervention in the treatment of acute severe pancreatitis?

**Recommendation 9.5:**

- In patients with infected necrosis, surgery should be delayed for at least 4 weeks to allow the development of a fibrous wall around the necrosis, except in cases requiring emergency surgical intervention. (Level of Evidence: 1A, Strength of recommendation: Strong consensus (100%)).

***Comment** **:*** Although the literature on the optimal timing of surgical intervention in the treatment of acute severe pancreatitis varies, RCTs and meta-analyses appear to have reached a consensus. Retrospective evaluations indicate that patients operated on after more than 3 weeks achieve better outcomes.^483^ Another retrospective analysis showed that delaying necrosectomy beyond 30 days after initial hospitalization is associated with reduced mortality, increased long-term antibiotic use, and higher incidence of Candida species and antibiotic-resistant organisms.^275^ One retrospective study reported a 50% increase in mortality when surgery is performed before 4 weeks, while another study extended this duration to 6 weeks.^484,485^ In a retrospective series of 131 patients, no differences were found between surgical interventions before and after 4 weeks in terms of organ failure, mortality, bleeding, fistula, and length of stay.^280^ A prospective study of 223 patients noted similar clinical outcomes for surgeries performed before and after 4 weeks, while another study reported acceptable mortality and complication rates for surgeries performed before 3 weeks.^287,486,487^ A meta-analysis of 7 clinical studies involving 742 patients with infected pancreatic necrosis revealed that patients undergoing early surgery had longer hospital stays and higher risks of gastrointestinal fistula and perforation, though no differences in mortality were observed.^283^

#### Question 9.6: What is the most appropriate surgical approach strategy in AP?

**Recommendation 9.6:**

- In acute necrotizing pancreatitis, open surgery should only be considered as a treatment method when other treatment options have failed or in cases requiring emergency surgery. When surgical treatment is necessary, minimally invasive surgical options should be prioritized based on the surgeon’s and center’s experience. A step-up approach should be preferred in the surgical strategy (Level of Evidence: 1A, Strength of recommendation: Strong consensus (98.7%)).
- In centers with a multidisciplinary expert group, surgical treatment should be individualized based on the center’s experience (Level of Evidence: 3, Strength of recommendation: Strong consensus (93.9%)).

***Comment** **:*** Different surgical strategies can be considered in the treatment of AP. Traditional open surgery is one of these options and can be performed transperitoneally, transgastrically, or retroperitoneally. Although studies comparing each method with each other exist, there is no strong evidence demonstrating superiority among them. In recent years, minimally invasive procedures have replaced open surgery. Examples of these include minimal access retroperitoneal pancreatic necrosectomy (MARPN), VARD, and laparoscopic abdominal approaches. There is insufficient evidence to determine which of these minimally invasive approaches might be superior. However, in general, open and minimally invasive methods have been compared. According to these comparison results, mortality rates in open surgery were found to be higher than those in minimally invasive procedures.^488^ In a study comparing minimally invasive retroperitoneal necrosectomy and open necrosectomy, the outcomes of 394 patients were evaluated, and it was concluded that the minimally invasive approach provided better treatment success rates and clinical outcomes compared to open surgery.^489^ Another comparative study showed that there was a higher mortality rate in high-risk patients undergoing open surgery.^490^

The success of minimally invasive approaches has led to the emergence of the step-up approach concept. This approach, defined as the progression of interventional procedures from the least invasive to the most invasive in patients with AP, has demonstrated efficacy in numerous studies. One of the most significant among these is the PANTER RCT.^491^ The results of this study recommended that interventions for necrotizing pancreatitis should be performed using the step-up approach.

Based on this information, it would be appropriate to prefer minimally invasive approaches over open surgery according to the center’s experience. However, in cases where other treatments have failed or in emergency surgeries, the value of open surgery is always unquestionable. In a multidisciplinary center, it is necessary for interventional radiologists, gastroenterologists, and surgeons to evaluate the patient together using a step-up approach and decide on the most suitable treatment.

## Conclusion

Acute pancreatitis is an acute inflammatory disease of the pancreas that can develop due to various causes. The most common etiologies of AP are gallstones and alcohol. The prevalence of these etiological factors can vary based on geographic, demographic, and genetic factors. Diagnosis is based on the clinical presentation of typical abdominal pain, laboratory findings of amylase and/or lipase levels more than 3 times the normal value, and supportive findings from imaging modalities such as TAUS, CT, or MRI. Patients are considered to have AP if they meet 2 of these 3 criteria. Transabdominal ultrasonography should be the primary imaging method due to its widespread use and its ability to provide valuable information for both diagnosing AP and evaluating etiological factors (distinguishing biliary from non-biliary causes). Rapid and accurate prediction of severe AP is essential to improve patient prognosis. There is no sufficient evidence or consensus on a “gold standard” biochemical parameter or prognostic score for predicting severe AP. Early fluid therapy is crucial in the treatment of AP. There is no sufficient evidence or consensus on the most appropriate analgesic and route of administration for pain management in AP. Prophylactic antibiotics are not recommended in AP, including severe pancreatitis and the presence of necrosis. However, antibiotics are recommended in the presence of infected necrosis and extrapancreatic infections. Patients with a BISAP score of 3 or higher at diagnosis, as well as those who experience moderate or severe attacks according to the revised Atlanta criteria during follow-up, should be promptly referred to a tertiary care center.

Pancreatic pseudocysts and necrosis should be managed conservatively unless symptomatic. Endoscopic drainage, due to its less invasive nature and high clinical success rates, should be the preferred approach for symptomatic PPs adjacent to the stomach or duodenum and for necrotic drainage. For patients with collections not suitable for endoscopic drainage, percutaneous drainage or minimally invasive surgery may be preferred. Anticoagulant therapy should be administered for isolated splenic vein thrombosis, mesenteric vein thrombosis, or portal vein thrombosis during the course of AP, provided there are no contraindications and no collateral vessels are present. A step-up approach may be recommended for the treatment of DPDS. There is insufficient evidence to suggest that specific treatments can reduce the frequency of attacks in RAP. Endoscopy plays a complementary role in the evaluation and treatment of RAP patients. While debates continue regarding the benefits of ERCP in unexplained RAP treatment, endoscopic interventions have been found safe and effective in treating various complications arising from CP. Managing these patients is challenging and requires a personalized, multidisciplinary approach. Pancreatic enzyme replacement therapy has been shown to improve symptoms, digestion, and overall health in pancreatitis cases. Pancreatic ascites can be treated with conservative, endoscopic, or surgical methods. Before making a treatment decision, the presence of pancreatic duct disruption and/or DPDS should be assessed. Rare complications such as pseudoaneurysm and DM can develop during the course of AP. Surgery in AP is most commonly indicated for treating complications, notably infected walled-off necrosis. In this scenario, debridement can be performed using endoscopic, percutaneous drainage, or minimally invasive surgical methods with a multidisciplinary approach. Open surgery is indicated only when these methods fail. Other surgical indications in AP include acute compartment syndrome, non-occlusive bowel ischemia and necrosis, enterocutaneous fistula, vascular complications, and PP treatment. Cholecystectomy also plays a role in preventing recurrent biliary AP.

## Supplementary Materials

Supplementary Material

## Figures and Tables

**Table 1.** Summary of Recommendations on the Management of Acute Pancreatitis

**Diagnosis**
Transabdominal ultrasonography (TAUS) can be used as a primary imaging method due to its ability to provide valuable information not only for diagnosing AP but also for etiological assessment, coupled with its widespread use. If the diagnosis of AP remains uncertain after TAUS, evaluation with computed tomography or magnetic resonance imaging is recommended (*Level of Evidence: 2B, Strength of recommendation: Strong consensus (89.4%)*).
**Initial Asessment and Risk Stratification**
The severity of AP is categorized as mild, moderate, or severe based on the presence of local and systemic complications, as well as the state of necrosis and infected necrosis. The revised Atlanta classification is the most commonly used classification for this purpose (*Level of Evidence: 1A, Strength of recommendation: Strong consensus (96.7%)*).
Elevated blood urea nitrogen (BUN) and serum creatinine levels during the course of AP or at 48 hours are associated with increased morbidity and mortality. C-reactive protein (CRP) level of 150 mg/L (15 mg/dL) at 48 hours can be used as an indicator of poor prognosis in AP (*Level of Evidence: 3, Strength of recommendation: Strong consensus (91.7%)*).
Given its simplicity in calculation and comparability to the acute physiology and chronic health evaluation II (APACHE II) score, the Bedside Index for Severity in Acute Pancreatitis (BISAP) score is the recommended scoring system for routine clinical practice (*Level of Evidence: 3, Strength of recommendation: Strong consensus (91.7%)*).
**Initial Management**
***Fluid Resuscitation***
The fluid used in treatment should be isotonic crystalloid (isotonic NaCl or Ringer’s lactate (RL)). If there is no contraindication specific to the patient (e.g., hypercalcemia), RL can be preferred (*Level of Evidence: 1A, Strength of recommendation: Strong consensus (94.7%)).*
There is insufficient evidence regarding the use of hydroxyethyl starch (HES) in AP treatment. Its use is not recommended in AP treatment except for abdominal compartment syndrome (*Level of Evidence: 1A, Strength of recommendation: Strong consensus (94.7%)).*
The rate of fluid resuscitation should be tailored according to the patient’s clinical assessment at presentation and follow-up data (targeted) (*Level of Evidence: 1A, Strength of recommendation: Strong consensus (94.7%)).*
Aggressive fluid therapy in AP, particularly in moderate to severe and severe AP patients, is not recommended as it increases the risk of systemic inflammatory response syndrome (SIRS), organ failure, the need for intensive care and ventilation, and the development of abdominal compartment syndrome (*Level of Evidence: 1A, Strength of recommendation: Strong consensus (94.7%)).*
***Pain Control***
In patients with mild AP, non-steroidal anti-inflammatory drugs (NSAIDs) (Indomethacin, metamizole, dexketoprofen, diclofenac) have similar efficacy to opioids in pain palliation during the first 24 hours and can be used as alternatives to opioids. They should not be used in patients with renal failure (*Level of Evidence: 1A, Strength of recommendation: Strong consensus (95.6%)).*
**Nutrition in AP**
Unless there is an obstruction or contraindication to oral feeding (e.g., ileus, abdominal compartment syndrome), oral intake should not be discontinued (*Level of Evidence: 1B, Strength of recommendation: Weak consensus (78.4%)).*
If oral feeding cannot be initiated within the first 72 hours, nutritional support should be provided. For patients who cannot tolerate oral feeding, enteral nutrition (EN) should be prioritized. Feeding should commence using a nasogastric (NG) or nasojejunal (NJ) tube (*Level of Evidence: 1A, Strength of recommendation: Weak consensus (79%)).*
For patients who cannot tolerate EN, where NG/NJ tube placement is not possible, or where target protein and calorie needs cannot be met by EN alone, parenteral nutrition (PN) should be administered (*Level of Evidence: 1A, Strength of recommendation: Strong consensus (89.9%)).*
Glutamine should be added to the nutritional solution for patients requiring nutritional support (*Level of Evidence: 1A, Strength of recommendation: Strong consensus (84.9%)).*
**The Role of Antibiotics in AP**
The use of prophylactic antibiotics is not recommended in AP, including severe pancreatitis and the presence of necrosis. However, antibiotics are recommended in cases of infected necrosis and extrapancreatic infections (*Level of Evidence: 1A, Strength of recommendation: Strong consensus (89.1%)).*
In AP, carbapenems, quinolones, metronidazole, and cephalosporins can be used. In the presence of infected necrosis, carbapenem antibiotics should be preferred (*Level of Evidence: 1A, Strength of recommendation: Strong consensus (95.4%)).*
**ERCP in AP**
In acute biliary pancreatitis, if there are signs of a stone impacted in the papilla or cholangitis, ERCP is recommended at the earliest possible stage. If these conditions are not present but there are signs of cholestasis, imaging of the common bile duct (endoscopic ultrasonography (EUS), magnetic resonance cholangiopancreatography (MRCP)) is recommended (*Level of Evidence: 1A, Strength of recommendation: Strong consensus (100%)).*
**Indications for Referral to a Tertiary Center and Admission to the Intensive Care Unit**
Patients with a BISAP score of 3 or higher at diagnosis, and those experiencing moderate or severe attacks according to the revised Atlanta criteria during follow-up should be promptly referred to a tertiary center. (*Level of Evidence: 1A, Strength of recommendation: Strong consensus (87.2%)).*
Patients with confirmed or strongly suspected biliary etiology (those considered for ERCP and/or cholecystectomy) should be referred to specialized centers. (*Level of Evidence: 2B, Strength of recommendation: Strong consensus (87.2%)).*
Patients with persistent organ dysfunction should be monitored in an intensive care unit. (*Level of Evidence: 1A, Strength of recommendation: Strong consensus (87.7%)).*
**Management of AP Complications**
***Peripancreatic Fluid Collections***
Pancreatic pseudocysts (PP) should be managed conservatively unless symptomatic. Indications for drainage include cyst infection, persistent intra-abdominal symptoms (abdominal pain, nausea, vomiting, early satiety), gastric outlet obstruction, and biliary obstruction with accompanying jaundice. (*Level of Evidence: 3, Strength of recommendation: Strong Consensus (96.8%)).*
Endoscopic drainage should be the preferred approach for draining PPs adjacent to the stomach or duodenum due to its less invasive nature and high clinical success rates. Surgical drainage may be considered for patients in whom endoscopic intervention fails and/or is anatomically unsuitable (*Level of Evidence:1A, Strength of recommendation: Strong Consensus (91.9%)).*
Percutaneous drainage can be preferred for cysts inaccessible via endoscopy or for patients with comorbidities precluding endoscopy or surgery (*Level of Evidence: 1A, Strength of recommendation: Strong Consensus (91.9%)).*
In patients with luminal compression, both conventional and EUS-guided drainage have similar technical success and complication rates. In cases of PP without luminal compression, in patients with coagulopathy, in the presence of cyst-adjacent vascular structures, and when complications arise during conventional procedures, EUS-guided drainage is specifically recommended (*Level of Evidence: 1A, Strength of recommendation: Strong consensus (93.6%)).*
Asymptomatic pancreatic and/or extrapancreatic necrosis do not require invasive intervention regardless of their size or location (*Level of Evidence: 3, Strength of recommendation: Strong consensus (94.3%)).*
After the diagnosis of infected pancreatic necrosis, patients should be closely monitored under appropriate antibiotic and nutritional support, if necessary, in intensive care settings. Waiting at least 4 weeks before invasive interventions is a more suitable approach in terms of potential complications. However, if the patient’s clinical condition deteriorates minimal invasive intervention should be considered irrespective of time. (*Level of Evidence: 1B, Strength of recommendation: Strong consensus (91.4%)).*
Endoscopic drainage is the preferred treatment for walled-off necrosis (WONs). In patients with collections that are not suitable for endoscopic drainage, minimally invasive surgery or percutaneous drainage may be the preferred approach (*Level of Evidence: 1A, Strength of recommendation: Strong consensus (100%)).*
Patients with WON that extends into the paracolic gutters or pelvis may require percutaneous drainage in addition to the endoscopic procedure (*Level of Evidence: 1A, Strength of recommendation: Strong consensus (100%)).*
***Disconnected Pancreatic Duct Syndrome (DPDS)***
A step-up approach may be recommended for DPDS. In endoscopic treatment, long-term transmural drainage (TMD) with plastic stents is sufficient for most patients (*Level of Evidence: 3, Strength of recommendation: Strong consensus (88.5%)).*
Transmural stents should be maintained for a long period. Before removal, imaging techniques (preferably secretin-enhanced MRCP) should confirm the absence of a pancreatic duct “feeding” the cyst (*Level of Evidence: 3, Strength of recommendation: Strong consensus (88.5%)).*
***Venous Thrombosis***
If isolated splenic vein thrombosis is present, the thrombus extends to the mesenteric vein, or there is a portal vein thrombosis without collateral formation at the time of detection and anticoagulant use is not contraindicated, anticoagulant therapy should be administered with careful consideration of bleeding risk, particularly in patients with pseudocysts (*Level of Evidence: 3, Strength of recommendation: Strong consensus (97%)).*
In patients starting anticoagulation therapy without an underlying thrombophilic disorder, the treatment duration should be 3-6 months (*Level of Evidence: 3, Strength of recommendation: Strong consensus (96%)).*
In patients with severe AP where no contraindications exist, short-term (7-14 days) prophylactic low-molecular-weight heparin (LMWH) therapy has beneficial effects on hospital stay, organ failure, and mortality (*Level of Evidence: 1A, Strength of recommendation: Strong consensus (95.1%)).*
**Management of Recurrent Acute Pancreatitis (RAP)**
Identification and treatment of the underlying etiological factor to reduce the number of attacks in RAP is recommended. However, there is insufficient evidence that specific treatments can reduce or prevent the number of RAP attacks (*Level of Evidence: 2A, Strength of recommendation: Strong consensus (95.1%)).*
In biliary RAP patients who cannot undergo cholecystectomy due to high surgical risk, or in post-cholecystectomy patients with biliary RAP, biliary endoscopic sphincterotomy (BES) may prevent new attacks (*Level of Evidence: 2A, Strength of recommendation: Strong Consensus (98.6%)).*
In RAP patients associated with pancreas divisum without chronic pancreatitis findings, minor papilla endoscopic sphincterotomy may prevent the development of new attacks (*Level of Evidence: 1A, Strength of recommendation: Strong Consensus (100%)).*
Endoscopic sphincterotomy is recommended in type I sphincter of Oddi dysfunction (SOD) and particularly in type II SOD with enzyme elevation (*Level of Evidence: 2A, Strength of recommendation: Strong Consensus (98.9%)).*
In cases of idiopathic RAP, although sufficient evidence is lacking, BES may be considered after investigating microlithiasis or other potential etiologies on a per-patient basis. Pancreatic endoscopic sphincterotomy is not routinely recommended (*Level of Evidence: 2A, Strength of recommendation: Strong Consensus (97.9%)).*
**Management of Long-term Complications of AP**
***Exocrine Pancreatic Insufficiency (EPI)***
Pancreatic enzyme replacement therapy (PERT) should be administered to patients with AP-induced EPI. The initial dose is 40 000-50 000 units at main meals and 25 000 units at snacks. Based on treatment response, doses can be increased to a maximum of 80 000 units at main meals and half of this amount at snacks (*Level of Evidence: 1B, Strength of Recommendation: Strong Consensus (92.3%)).*
A dietary plan with frequent, small-volume meals is recommended. At least one meal should include a normal amount of fat (*Level of Evidence: 3, Strength of Recommendation: Strong Consensus (91.7%)).*
Periodic screenings for nutritional deficiencies (fat-soluble vitamins, magnesium, zinc, vitamin B12) should be conducted, and supplementation should be provided if deficiencies are detected (*Level of Evidence: 1B, Strength of Recommendation: Strong Consensus (94.3%)).*
***Pancreatic Ascites***
Endoscopic treatment methods should be preferred in suitable cases. In cases of partial pancreatic duct disruption, transpapillary endoscopic drainage is an appropriate method (*Level of Evidence: 3, Strength of Recommendation: Strong Consensus (95.4%)).*
Percutaneous drainage can be applied in the event of increased pain, clinical deterioration, new-onset organ failure, or abdominal compartment syndrome. Surgery should be considered in cases where endoscopic treatments are inappropriate or unsuccessful. (*Level of Evidence: 3, Strength of Recommendation: Strong Consensus (88%)).*
***Pseudoaneurysm***
Pseudoaneurysm should be suspected in cases of abdominal pain, a drop in hemoglobin (gastrointestinal and intra-abdominal bleeding), and sudden growth of the cystic lesion. Endovascular embolization (coil) is the first treatment option. If this fails, surgical treatment may be applied (*Level of Evidence: 1A, Strength of recommendation: Strong Consensus (98.7%)).*
***Diabetes Mellitus (DM)***
Metformin is effective in the treatment of DM after AP. Insulin therapy may be needed earlier compared to type 2 DM (*Level of Evidence: 1B, Strength of Recommendation: Strong consensus (89.9%)).*
**The Role of Surgery in AP**
***Cholecystectomy in AP***
In mild biliary AP, the patient should ideally be recommended cholecystectomy after the pancreatitis has subsided, preferably during the hospital stay and within 4 weeks if possible (*Level of Evidence: 1A, Strength of Recommendation: Strong consensus (96.1%)).*
Delaying cholecystectomy following moderate and severe biliary AP reduces morbidity. In patients who have survived an episode of moderate to severe acute biliary pancreatitis and present with pancreatic fluid collections, cholecystectomy should be postponed for 6-8 weeks (*Level of Evidence: 3, Strength of recommendation: Strong consensus (95.2%)).*
Following an episode of AP with no identifiable cause, cholecystectomy should be considered in patients suitable for surgery to reduce the risk of recurrent pancreatitis attacks (*Level of Evidence: 1A, Strength of recommendation: Strong consensus (80.1%)).*
***Indications for Surgical Intervention***
Fistulization of the peripancreatic collection to the colon, intestinal ischemia, abdominal compartment syndrome where conservative and noninvasive treatments have failed, perforation, gastric outlet obstruction, intestinal obstruction, acute necrotizing cholecystitis, and bleeding where the endovascular approach has failed (*Level of evidence: 3, Strength of recommendation: Strong consensus (96.3%)).*
In patients with infected necrosis, surgery should be delayed for at least 4 weeks to allow the development of a fibrous wall around the necrosis, except in cases requiring emergency surgical intervention (*Level of Evidence: 1A, Strength of recommendation: Strong consensus (100%)).*
In acute necrotizing pancreatitis, open surgery should only be considered as a treatment method when other treatment options have failed or in cases requiring emergency surgery. When surgical treatment is necessary, minimally invasive surgical options should be prioritized. A step-up approach should be preferred (*Level of Evidence: 1A, Strength of recommendation: Strong consensus (98.7%)).*

AP, acute pancreatitis; APACHE II, acute physiology and chronic health evaluation II; BES, biliary endoscopic sphincterotomy; BISAP, bedside index for severity in acute pancreatitis; BUN, blood urea nitrogen; CRP, C-reactive protein; DM, diabetes mellitus; DPDS, disconnected pancreatic duct syndrome; EN, enteral nutrition; ERCP, endoscopic retrograde cholangiopancreatography; EPI, exocrine pancreatic insufficiency; EUS, endoscopic ultrasonography; HES, hydroxyethyl starch; LMWH, low-molecular-weight heparin; MRCP, magnetic resonance cholangiopancreatography; NG, nasogastric; NJ, nasojejunal; NSAIDs, non-steroidal anti-inflammatory drugs; PERT, Pancreatic enzyme replacement therapy; PN, parenteral nutrition; PP, pancreatic pseudocysts; RAP, management of recurrent acute pancreatitis; RL, Ringer’s lactate; SIRS, systemic inflammatory response syndrome; SOD, sphincter of Oddi dysfunction; TAUS, transabdominal ultrasonography; TMD, transmural drainage; WONs, walled-off necrosis.

**Supplementary Table 1.** Level of Evidence Classification

**Level of Evidence**	**Criteria**
1a	Systematic reviews (meta-analyses) containing at least some trials of level 1b evidence, in which the results of separate, independently controlled trials are consistent
1b	Randomized controlled trial of good quality and of adequate sample size (power calculations)
2a	Randomized trials of reasonable quality and/or inadequate sample size
2b	Nonrandomized trials, comparative research (parallel cohort)
2c	Nonrandomized trials, comparative research (historical cohort, literature controls)
3	Nonrandomized, non-comparative trials, descriptive research
4	Expert opinions, including the opinion of work group members

**Supplementary Table 2.** Revised Atlanta Criteria for Severity of Acute Pancreatitis

**Severity Grades**	**Criteria**
Mild	No organ failure No local or systemic complications
Moderate	Transient organ failure (resolves within 48 hours) and/or Local or systemic complications without persistent organ failure
Severe	Persistent organ failure (duration of >48 hours) Single organ failure Multiple organ failure

Local complications: Pancreatic/peripancreatic necrosis (sterile or infected), peripancreatic fluid collections, pseudocyst, walled-off necrosis (sterile or infected).

**Supplementary Table 3.** BISAP Scoring System

Blood urea nitrogen (BUN)	BUN >25 mg/dL (8.9 mmol/L) (1 point)
Impaired mental status	Abnormal mental status with a Glasgow coma score <15 (1 point)
SIRS	Evidence of SIRS (1 point)
Age	Age >60 years old (1 point)
Pleural effusion	Imaging study reveals pleural effusion (1 point)

0-2 points: Lower mortality (<2%)

3-5 points: Higher mortality (>15%)

SIRS (Systemic Inflammatory Response Syndrome) is diagnosed by the presence of any 2 of the following criteria:

1) Temperature (<36°C or >38°C),

2) Pulse >90/min,

3) Respiratory rate >20 or PaCO_2_ <32mmHg, and 4) WBC > 12 000/mm^3^ or <4000/mm^3^ or >10% bands.

BUN, blood urea nitrogen; SIRS, systemic inflammatory response syndrome.

## Data Availability

The data that support the findings of this study are available on request from the corresponding author.

## References

[1] Iannuzzi JP King JA Leong JH , et al. Global incidence of acute pancreatitis is increasing over time: a systematic review and meta-analysis. Gastroenterology. 2022;162(1):122 134. (10.1053/j.gastro.2021.09.043) 34571026

[2] Li CL Jiang M Pan CQ Li J Xu LG . The global, regional, and national burden of acute pancreatitis in 204 countries and territories, 1990-2019. BMC Gastroenterol. 2021;21(1):332. (10.1186/s12876-021-01906-2) 34433418 PMC8390209

[3] Roberts SE Morrison-Rees S John A Williams JG Brown TH Samuel DG . The incidence and aetiology of acute pancreatitis across Europe. Pancreatology. 2017;17(2):155 165. (10.1016/j.pan.2017.01.005) 28159463

[4] Asfuroğlu Kalkan E Kalkan Ç Kaçar S , et al. Similarities and differences between gerontal and young patients with acute pancreatitis: evaluation of clinical characteristics and outcomes. Turk J Gastroenterol. 2022;33(10):874 884. (10.5152/tjg.2022.22227) 36205509 PMC9623137

[5] Drake M Dodwad SJM Davis J Kao LS Cao Y Ko TC . Sex-related differences of acute and chronic pancreatitis in adults. J Clin Med. 2021;10(2):300. (10.3390/jcm10020300) 33467580 PMC7830423

[6] Hamada S Masamune A Shimosegawa T . Management of acute pancreatitis in Japan: analysis of nationwide epidemiological survey. World J Gastroenterol. 2016;22(28):6335 6344. (10.3748/wjg.v22.i28.6335) 27605870 PMC4968116

[7] Ho UC Mu CF Hsu CY . Ethnic differences in risk factors of acute pancreatitis. Ethn Health. 2018;23(3):321 328. (10.1080/13557858.2016.1263287) 27905207

[8] Petrov MS Yadav D . Global epidemiology and holistic prevention of pancreatitis. Nat Rev Gastroenterol Hepatol. 2019;16(3):175 184. (10.1038/s41575-018-0087-5) 30482911 PMC6597260

[9] Song K Guo C Li C Ding N . Risk factors of recurrence of acute pancreatitis: a retrospective research. Turk J Gastroenterol. 2021;32(11):971 978. (10.5152/tjg.2020.20775) 34872898 PMC8975497

[10] Huang Q Wu Z Zhang Y Wu Y Shi C Liu Y . Obesity exacerbates acute gastrointestinal injury and intestinal barrier dysfunction in early-stage acute pancreatitis. Turk J Gastroenterol. 2023;34(4):421 426. (10.5152/tjg.2023.22145) 36806182 PMC10210755

[11] Köksal AŞ Tozlu M Sezgin O , et al. Acute pancreatitis in Turkey: results of a nationwide multicenter study. Pancreatology. 2024;24(3):327 334. (10.1016/j.pan.2023.10.005) 37880021

[12] Zilio MB Eyff TF Azeredo-Da-Silva ALF Bersch VP Osvaldt AB . A systematic review and meta-analysis of the aetiology of acute pancreatitis. HPB (Oxford). 2019;21(3):259 267. (10.1016/j.hpb.2018.08.003) 30249509

[13] Gullo L Migliori M Oláh A , et al. Acute pancreatitis in five European countries: etiology and mortality. Pancreas. 2002;24(3):223 227. (10.1097/00006676-200204000-00003) 11893928

[14] Yadav D Lowenfels AB . Trends in the epidemiology of the first attack of acute pancreatitis: a systematic review. Pancreas. 2006;33(4):323 330. (10.1097/01.mpa.0000236733.31617.52) 17079934

[15] Khan MA Khan Z Tombazzi CR Gadiparthi C Lee W Wilcox CM . Role of cholecystectomy after endoscopic sphincterotomy in the management of choledocholithiasis in high-risk patients: a systematic review and meta-analysis. J Clin Gastroenterol. 2018;52(7):579 589. (10.1097/MCG.0000000000001076) 29912758

[16] van Geenen EJM van der Peet DL Bhagirath P Mulder CJJ Bruno MJ . Etiology and diagnosis of acute biliary pancreatitis. Nat Rev Gastroenterol Hepatol. 2010;7(9):495 502. (10.1038/nrgastro.2010.114) 20703238

[17] Lankisch PG Apte M Banks PA . Acute pancreatitis. Lancet. 2015;386(9988):85 96. (10.1016/S0140-6736(14)60649-8) 25616312

[18] Irving HM Samokhvalov AV Rehm J . Alcohol as a risk factor for pancreatitis. A systematic review and meta-analysis. J Pancreas. 2009;10(4):387 392. PMC3299482 19581740

[19] Waldthaler A Schütte K Malfertheiner P . Causes and mechanisms in acute pancreatitis. Dig Dis. 2010;28(2):364 372. (10.1159/000319416) 20814214

[20] Chen EX Tu Ya SQ She ZF , et al. The clinical characteristic of alcohol-hyperlipidemia etiologically complex type of acute pancreatitis. Eur Rev Med Pharmacol Sci. 2022;26(19):7212 7218. (10.26355/eurrev_202210_29913) 36263531

[21] Suzuki M Minowa K Nakano S Isayama H Shimizu T . Genetic abnormalities in pancreatitis: an update on diagnosis, clinical features, and treatment. Diagnostics (Basel). 2020;11(1):31. (10.3390/diagnostics11010031) 33375361 PMC7824215

[22] Bálint ER Fűr G Kiss L , et al. Assessment of the course of acute pancreatitis in the light of aetiology: a systematic review and meta-analysis. Sci Rep. 2020;10(1):17936. (10.1038/s41598-020-74943-8) 33087766 PMC7578029

[23] Simons-Linares CR Imam Z Chahal P . Viral-attributed acute pancreatitis: a systematic review. Dig Dis Sci. 2021;66(7):2162 2172. (10.1007/s10620-020-06531-9) 32789532

[24] Eldaly AS Fath AR Mashaly SM Elhadi M . Acute pancreatitis associated with severe acute respiratory syndrome coronavirus-2 infection: a case report and review of the literature. J Med Case Rep. 2021;15(1):461. (10.1186/s13256-021-03026-7) 34503570 PMC8426586

[25] Balthazar JA Chehter EZ . Acute pancreatitis and COVID-19: a new target for infection? Einstein (Sao Paulo). 2022;20:eRW6667. (10.31744/einstein_journal/2022RW6667) 35195163 PMC8815337

[26] Onoyama T Koda H Hamamoto W , et al. Review on acute pancreatitis attributed to COVID-19 infection. World J Gastroenterol. 2022;28(19):2034 2056. (10.3748/wjg.v28.i19.2034) 35664035 PMC9134139

[27] Rawla P Bandaru SS Vellipuram AR . Review of infectious etiology of acute pancreatitis. Gastroenterology Res. 2017;10(3):153 158. (10.14740/gr858w) 28725301 PMC5505279

[28] Majumder S Gierisch JM Bastian LA . The association of smoking and acute pancreatitis: a systematic review and meta-analysis. Pancreas. 2015;44(4):540 546. (10.1097/MPA.0000000000000301) 25872130

[29] Sun X Huang X Zhao R Chen B Xie Q . Meta-analysis: tobacco smoking may enhance the risk of acute pancreatitis. Pancreatology. 2015;15(3):286 294. (10.1016/j.pan.2015.03.001) 25804129

[30] Yuhara H Ogawa M Kawaguchi Y Igarashi M Mine T . Smoking and risk for acute pancreatitis: a systematic review and meta-analysis. Pancreas. 2014;43(8):1201 1207. (10.1097/MPA.0000000000000176) 25333404

[31] Jøergensen MT Brusgaard K Novovic S , et al. Is the SPINK1 variant p.N34S overrepresented in patients with acute pancreatitis? Eur J Gastroenterol Hepatol. 2012;24(3):309 315. (10.1097/MEG.0b013e32834ee5e2) 22228370

[32] Rebours V Lévy P Ruszniewski P . An overview of hereditary pancreatitis. Dig Liver Dis. 2012;44(1):8 15. (10.1016/j.dld.2011.08.003) 21907651

[33] Panchoo AV VanNess GH Rivera-Rivera E Laborda TJ . Hereditary pancreatitis: an updated review in pediatrics. World J Clin Pediatr. 2022;11(1):27 37. (10.5409/wjcp.v11.i1.27) 35096544 PMC8771313

[34] Kopacova M Tacheci I Rejchrt S Bartova J Bures J . Double balloon enteroscopy and acute pancreatitis. World J Gastroenterol. 2010;16(19):2331 2340. (10.3748/wjg.v16.i19.2331) 20480517 PMC2874136

[35] Halpern B Paulin Sorbello M Telles Libanori H Correa Mancini M . Extrinsic compression of pancreactic duct by intragastric balloon treatment and its potential to cause acute pancreatitis: two case reports and clinical discussion. Obes Res Clin Pract. 2020;14(2):191 193. (10.1016/j.orcp.2020.02.002) 32127292

[36] Liu F Zhang L Fu P , et al. Severe crush syndrome complicated with acute pancreatitis: a case report and review of the literatures. Chin Med J (Engl). 2009;122(24):3103 3105. 20137512

[37] Tauchi R Imagama S Ito Z , et al. Acute pancreatitis after spine surgery: a case report and review of literature. Eur J Orthop Surg Traumatol. 2014;24(suppl 1):S305 S309. (10.1007/s00590-013-1390-z) 24318308

[38] Hershberger RC Bornak A Aulivola B Mannava K . Acute pancreatitis after percutaneous mechanical thrombectomy: case report and review of the literature. Cardiovasc Intervent Radiol. 2011;34(suppl 2):S25 S30. (10.1007/s00270-010-0027-4) 21076922

[39] DiMagno MJ Wamsteker EJ . Pancreas divisum. Curr Gastroenterol Rep. 2011;13(2):150 156. (10.1007/s11894-010-0170-8) 21222060 PMC3079411

[40] Alempijevic T Stimec B Kovacevic N . Anatomical features of the minor duodenal papilla in pancreas divisum. Surg Radiol Anat. 2006;28(6):620 624. (10.1007/s00276-006-0155-3) 17024308

[41] Kin T Shapiro AMJ Lakey JRT . Pancreas divisum: a study of the cadaveric donor pancreas for islet isolation. Pancreas. 2005;30(4):325 327. (10.1097/01.mpa.0000160280.56828.3f) 15841041

[42] Seike T Komura T Shimizu Y , et al. A case of chronic pancreatitis exacerbation associated with pancreatic arteriovenous malformation: a case report and literature review. Clin J Gastroenterol. 2019;12(2):135 141. (10.1007/s12328-018-0901-1) 30182162

[43] Salemis NS Liatsos C Kolios M Gourgiotis S . Recurrent acute pancreatitis secondary to a duodenal duplication cyst in an adult: a case report and literature review. Can J Gastroenterol. 2009;23(11):749 752. (10.1155/2009/979431) 19893770 PMC2777087

[44] Alzerwi NAN . Recurrent ascending cholangitis with acute pancreatitis and pancreatic atrophy caused by a juxtapapillary duodenal diverticulum: a case report and literature review. Med (Baltim). 2020;99(27):e21111. (10.1097/MD.0000000000021111) PMC7337422 32629744

[45] Imam Z Hanna A Jomaa D Khasawneh M Abonofal A Murad MH . Hypercalcemia of malignancy and acute pancreatitis. Pancreas. 2021;50(2):206 213. (10.1097/MPA.0000000000001741) 33565797

[46] Jiajue R Song A Wang O Li W . Persistent hypercalcemia crisis and recurrent acute pancreatitis due to multiple ectopic parathyroid carcinomas: case report and literature review of mediastinal parathyroid carcinoma. Front Endocrinol (Lausanne). 2020;11:647. (10.3389/fendo.2020.00647) 33013712 PMC7511513

[47] Wang F Wang NS Zhao BH Tang LQ . Acute pancreatitis as an initial symptom of systemic lupus erythematosus: a case report and review of the literature. World J Gastroenterol. 2005;11(30):4766 4768. (10.3748/wjg.v11.i30.4766) 16094728 PMC4615429

[48] Papadakis EP Sarigianni M Mikhailidis DP Mamopoulos A Karagiannis V . Acute pancreatitis in pregnancy: an overview. Eur J Obstet Gynecol Reprod Biol. 2011;159(2):261 266. (10.1016/j.ejogrb.2011.07.037) 21840110

[49] Földi M Gede N Kiss S , et al. The characteristics and prognostic role of acute abdominal on-admission pain in acute pancreatitis: a prospective cohort analysis of 1432 cases. Eur J Pain. 2022;26(3):610 623. (10.1002/ejp.1885) 34758174 PMC9299627

[50] Chaffin H Trivedi S Singh VP . Impact of abdominal imaging on the diagnosis of acute pancreatitis in patients with painless lipase elevation. Pancreatology. 2022;22(5):547 552. (10.1016/j.pan.2022.04.013) 35523703 PMC9809038

[51] Cappell MS . Acute pancreatitis: etiology, clinical presentation, diagnosis, and therapy. Med Clin North Am. 2008;92(4):889 923. (10.1016/j.mcna.2008.04.013) 18570947

[52] Párniczky A Kui B Szentesi A , et al. Prospective, multicentre, nationwide clinical data from 600 cases of acute pancreatitis. PLoS One. 2016;11(10):e0165309. (10.1371/journal.pone.0165309) 27798670 PMC5087847

[53] Rompianesi G Hann A Komolafe O Pereira SP Davidson BR Gurusamy KS . Serum amylase and lipase and urinary trypsinogen and amylase for diagnosis of acute pancreatitis. Cochrane Database Syst Rev. 2017;4(4):CD012010. (10.1002/14651858.CD012010.pub2) 28431198 PMC6478262

[54] Ismail OZ Bhayana V . Lipase or amylase for the diagnosis of acute pancreatitis? Clin Biochem. 2017;50(18):1275 1280. (10.1016/j.clinbiochem.2017.07.003) 28720341

[55] Smith RC Southwell-Keely J Chesher D . Should serum pancreatic lipase replace serum amylase as a biomarker of acute pancreatitis? ANZ J Surg. 2005;75(6):399 404. (10.1111/j.1445-2197.2005.03391.x) 15943725

[56] Lippi G Valentino M Cervellin G . Laboratory diagnosis of acute pancreatitis: in search of the Holy Grail. Crit Rev Clin Lab Sci. 2012;49(1):18 31. (10.3109/10408363.2012.658354) 22339380

[57] Chang K Lu W Zhang K , et al. Rapid urinary trypsinogen-2 test in the early diagnosis of acute pancreatitis: a meta-analysis. Clin Biochem. 2012;45(13-14):1051 1056. (10.1016/j.clinbiochem.2012.04.028) 22575591

[58] Dimastromatteo J Brentnall T Kelly KA . Imaging in pancreatic disease. Nat Rev Gastroenterol Hepatol. 2017;14(2):97 109. (10.1038/nrgastro.2016.144) 27826137

[59] O’Connor OJ McWilliams S Maher MM . Imaging of acute pancreatitis. AJR Am J Roentgenol. 2011;197(2):W221 W225. (10.2214/AJR.10.4338) 21785045

[60] Sun H Jian S Peng B Hou J . Comparison of magnetic resonance imaging and computed tomography in the diagnosis of acute pancreatitis: a systematic review and meta-analysis of diagnostic test accuracy studies. Ann Transl Med. 2022;10(7):410. (10.21037/atm-22-812) 35530935 PMC9073804

[61] Kim YK Ko SW Kim CS Hwang SB . Effectiveness of MR imaging for diagnosing the mild forms of acute pancreatitis: comparison with MDCT. J Magn Reson Imaging. 2006;24(6):1342 1349. (10.1002/jmri.20801) 17083111

[62] Kothari S Kalinowski M Kobeszko M Almouradi T . Computed tomography scan imaging in diagnosing acute uncomplicated pancreatitis: usefulness vs cost. World J Gastroenterol. 2019;25(9):1080 1087. (10.3748/wjg.v25.i9.1080) 30862996 PMC6406186

[63] Porter KK Zaheer A Kamel IR , et al. ACR appropriateness Criteria® acute pancreatitis. J Am Coll Radiol. 2019;16(11):S316 S330. (10.1016/j.jacr.2019.05.017) 31685100

[64] Şurlin V Săftoiu A Dumitrescu D . Imaging tests for accurate diagnosis of acute biliary pancreatitis. World J Gastroenterol. 2014;20(44):16544 16549. (10.3748/wjg.v20.i44.16544) 25469022 PMC4248197

[65] Banks PA Bollen TL Dervenis C , et al. Classification of acute pancreatitis—2012: revision of the Atlanta classification and definitions by international consensus. Gut. 2013;62(1):102 111. (10.1136/gutjnl-2012-302779) 23100216

[66] Leppäniemi A Tolonen M Tarasconi A , et al. 2019 WSES guidelines for the management of severe acute pancreatitis. World J Emerg Surg. 2019;14(1):27. (10.1186/s13017-019-0247-0) 31210778 PMC6567462

[67] Van Santvoort HC Bakker OJ Bollen TL , et al. A conservative and minimally invasive approach to necrotizing pancreatitis improves outcome. Gastroenterology. 2011;141(4):1254 1263. (10.1053/j.gastro.2011.06.073) 21741922

[68] Brindise E Elkhatib I Kuruvilla A Silva R . Temporal trends in incidence and outcomes of acute pancreatitis in hospitalized patients in the United States from 2002 to 2013. Pancreas. 2019;48(2):169 175. (10.1097/MPA.0000000000001228) 30629021

[69] Hirota M Takada T Kawarada Y , et al. JPN Guidelines for the management of acute pancreatitis: severity assessment of acute pancreatitis. J Hepatobil Pancreat Surg. 2006;13(1):33 41. (10.1007/s00534-005-1049-1) PMC2779364 16463209

[70] Wu BU Johannes RS Sun X Tabak Y Conwell DL Banks PA . The early prediction of mortality in acute pancreatitis: a large population-based study. Gut. 2008;57(12):1698 1703. (10.1136/gut.2008.152702) 18519429

[71] Dancu GM Popescu A Sirli R , et al. The BISAP score, NLR, CRP, or BUN: which marker best predicts the outcome of acute pancreatitis? Med (Baltim). 2021;100(51):e28121. (10.1097/MD.0000000000028121) PMC8702250 34941057

[72] Valverde-López F Matas-Cobos AM Alegría-Motte C Jiménez-Rosales R Úbeda-Muñoz M Redondo-Cerezo E . BISAP, RANSON, lactate and others biomarkers in prediction of severe acute pancreatitis in a European cohort. J Gastroenterol Hepatol. 2017;32(9):1649 1656. (10.1111/jgh.13763) 28207167

[73] Chen L Lu G Zhou Q Zhan Q . Evaluation of the BISAP score in predicting severity and prognoses of acute pancreatitis in Chinese patients. Int Surg. 2013;98(1):6 12. (10.9738/0020-8868-98.1.6) 23438270 PMC3723156

[74] Yadav J Yadav SK Kumar S , et al. Predicting morbidity and mortality in acute pancreatitis in an Indian population: a comparative study of the BISAP score, Ranson’s score and CT severity index. Gastroenterol Rep (Oxf). 2016;4(3):216 220. (10.1093/gastro/gov009) 25733696 PMC4976677

[75] Cho JH Kim TN Chung HH Kim KH . Comparison of scoring systems in predicting the severity of acute pancreatitis. World J Gastroenterol. 2015;21(8):2387 2394. (10.3748/wjg.v21.i8.2387) 25741146 PMC4342915

[76] Gezer NS Bengi G Baran A , et al. Comparison of radiological scoring systems, clinical scores, neutrophil-lymphocyte ratio and serum C-reactive protein level for severity and mortality in acute pancreatitis. Rev Assoc Med Bras (1992). 2020;66(6):762 770. (10.1590/1806-9282.66.6.762) 32696885

[77] Bardakçı O Akdur G Das M Sıddıkoğlu D Akdur O Beyazit Y . Comparison of different risk stratification systems for prediction of acute pancreatitis severity in patients referred to the emergency department of a tertiary care hospital. Ulus Travma Acil Cerrahi Derg. 2022;28(7):967 973. (10.14744/tjtes.2021.51892) 35775674 PMC10493842

[78] Chatterjee R Parab N Sajjan B Nagar VS . Comparison of acute physiology and chronic health evaluation II, modified computed tomography severity index, and bedside index for severity in acute pancreatitis score in predicting the severity of acute pancreatitis. Indian J Crit Care Med. 2020;24(2):99 103. (10.5005/jp-journals-10071-23343) 32205940 PMC7075053

[79] Kim BG Noh MH Ryu CH , et al. A comparison of the BISAP score and serum procalcitonin for predicting the severity of acute pancreatitis. Korean J Intern Med. 2013;28(3):322 329. (10.3904/kjim.2013.28.3.322) 23682226 PMC3654130

[80] Sternby H Hartman H Johansen D Thorlacius H Regnér S . IL-6 and CRP are superior in early differentiation between mild and non-mild acute pancreatitis. Pancreatology. 2017;17(4):550 554. (10.1016/j.pan.2017.05.392) 28610827

[81] Sandberg AA Borgström A . Early prediction of severity in acute pancreatitis. Is this possible? JOP. 2002;3(5):116 125. 12221326

[82] Chen L Jiang J . The diagnostic value of procalcitonin in patients with severe acute pancreatitis: a meta-analysis. Turk J Gastroenterol. 2022;33(9):722 730. (10.5152/tjg.2022.22098) 36134549 PMC9524488

[83] Mofidi R Suttie SA Patil PV Ogston S Parks RW . The value of procalcitonin at predicting the severity of acute pancreatitis and development of infected pancreatic necrosis: systematic review. Surgery. 2009;146(1):72 81. (10.1016/j.surg.2009.02.013) 19541012

[84] Tsuji Y Takahashi N Isoda H , et al. Early diagnosis of pancreatic necrosis based on perfusion CT to predict the severity of acute pancreatitis. J Gastroenterol. 2017;52(10):1130 1139. (10.1007/s00535-017-1330-5) 28374057

[85] Mao EQ Tang YQ Fei J , et al. Fluid therapy for severe acute pancreatitis in acute response stage. Chin Med J (Engl). 2009;122(2):169 173. (10.3760/cma.j.issn.0366-6999.2009.02.011) 19187641

[87] Buxbaum JL Quezada M Da B , et al. Early aggressive hydration hastens clinical improvement in mild acute pancreatitis. Am J Gastroenterol. 2017;112(5):797 803. (10.1038/ajg.2017.40) 28266591

[88] Gad MM Simons-Linares CR . Is aggressive intravenous fluid resuscitation beneficial in acute pancreatitis? A meta-analysis of randomized control trials and cohort studies. World J Gastroenterol. 2020;26(10):1098 1106. (10.3748/wjg.v26.i10.1098) 32206000 PMC7081000

[89] Lee A Ko C Buitrago C , et al. Lactated Ringers vs normal saline resuscitation for mild acute pancreatitis: a randomized trial. Gastroenterology. 2021;160(3):955 957.e4. (10.1053/j.gastro.2020.10.044) 33159924

[90] Karki B Thapa S Khadka D , et al. Intravenous Ringers lactate versus normal saline for predominantly mild acute pancreatitis in a Nepalese Tertiary Hospital. PLoS One. 2022;17(1):e0263221. (10.1371/journal.pone.0263221) 35089964 PMC9126573

[91] Guzmán-Calderón E Diaz-Arocutipa C Monge E . Lactate Ringer’s versus normal saline in the management of acute pancreatitis: a systematic review and meta-analysis of randomized controlled trials. Dig Dis Sci. 2022;67(8):4131 4139. (10.1007/s10620-021-07269-8) 34635979

[92] Kayhan S Selcan Akyol B Ergul M Baysan C . The effect of type of fluid on disease severity in acute pancreatitis treatment. Eur Rev Med Pharmacol Sci. 2021;25(23):7460 7467. (10.26355/eurrev_202112_27443) 34919248

[93] Aziz M Ahmed Z Weissman S , et al. Lactated Ringer’s vs normal saline for acute pancreatitis: an updated systematic review and meta-analysis. Pancreatology. 2021;21(7):1217 1223. (10.1016/j.pan.2021.06.002) 34172360

[94] Chen H Lu X Xu B Meng C Xie D . Lactated Ringer solution is superior to normal saline solution in managing acute pancreatitis: an updated meta-analysis of randomized controlled trials. J Clin Gastroenterol. 2022;56(2):e114 e120. (10.1097/MCG.0000000000001656) 35104255

[95] Du XJ Hu WM Xia Q , et al. Hydroxyethyl starch resuscitation reduces the risk of intra-abdominal hypertension in severe acute pancreatitis. Pancreas. 2011;40(8):1220 1225. (10.1097/MPA.0b013e3182217f17) 21775917

[96] Zhao G Zhang JG Wu HS , et al. Effects of different resuscitation fluid on severe acute pancreatitis. World J Gastroenterol. 2013;19(13):2044 2052. (10.3748/wjg.v19.i13.2044) 23599623 PMC3623981

[97] Myburgh JA Finfer S Bellomo R , et al. Hydroxyethyl starch or saline for fluid resuscitation in intensive care. N Engl J Med. 2012;367(20):1901 1911. (10.1056/NEJMoa1209759) 23075127

[98] Mahapatra SJ Jain S Bopanna S , et al. Pentazocine, a kappa-opioid agonist, is better than diclofenac for analgesia in acute pancreatitis: a randomized controlled trial. Am J Gastroenterol. 2019;114(5):813 821. (10.14309/ajg.0000000000000224) 31008736

[99] Stigliano S Sternby H de Madaria E Capurso G Petrov MS . Early management of acute pancreatitis: a review of the best evidence. Dig Liver Dis. 2017;49(6):585 594. (10.1016/j.dld.2017.01.168) 28262458

[100] Meng W Yuan J Zhang C , et al. Paranteral analgesics for pain relief in acute pancreatitis: a systematic review. Pancreatology. 2013;13(3):201 206. (10.1016/j.pan.2013.02.003) 23719588

[101] Wu D Bai X Lee P , et al. A systematic review of NSAIDs treatment for acute pancreatitis in animal studies and clinical trials. Clin Res Hepatol Gastroenterol. 2020;44:100002. (10.1016/j.clirex.2019.100002) 33602483

[102] Schorn S Ceyhan GO Tieftrunk E , et al. Pain management in acute pancreatitis. Pancreapedia. 2015. (10.3998/panc.2015.15)

[103] Runzi M Layer P Buchler MW , et al. The therapy of acute pancreatitis. General guidelines. Working group of the German Society of Gastroenterol. Z Gastroenterol. 2020;38:571.e81. (10.1055/s-2000-7447) 10965554

[104] Salicath JH Yeoh ECY Bennett MH . Epidural analgesia versus- patient-controlled intravenous analgesia for pain following intra-abdominal surgeryin adults. Cochrane Database Syst Rev. 2018;8(8):CD010434. (10.1002/14651858.CD010434.pub2) 30161292 PMC6513588

[105] Cebriana JG Bello Camara MP Rodriguez Yanaza JC Fernando Ruiz YA . Analgesia and sedation in acute pancreatitis. Medicana Intensiva. 2003;27(2):116 128.

[106] Thavanesan N White S Lee S , et al. Analgesia in the initial management of acute pancreatitis: a systematic review and meta-analysis of randomised controlled trials. World J Surg. 2022;46(4):878 890. (10.1007/s00268-021-06420-w) 34994837

[107] Cai W Liu F Wen Y , et al. Pain management in acute pancreatitis: a systematic review and meta-analysis of randomised controlled trials. Front Med (Lausanne). 2021;8:782151. (10.3389/fmed.2021.782151) 34977084 PMC8718672

[108] Layer P Bronisch HJ Henniges UM , et al. Effects of systematic administration of a local anesthetic on pain in acute pancreatitis: a randomized clinical trial. Pancreas. 2011;40(5):673 679. (10.1097/MPA.0b013e318215ad38) 21562445

[109] Chowdhury AR Chang P Zhou S , et al. Optimal initial diet in mild acute pancreatitis: a comprehensive meta-analysis of randomized control trials. Pancreatology. 2022;22(7):858 863. (10.1016/j.pan.2022.07.016) 35989218

[110] Eckerwall GE Tingstedt BBA Bergenzaun PE Andersson RG . Immediate oral feeding in patients with mild acute pancreatitis is safe and may accelerate recovery--a randomized clinical study. Clin Nutr. 2007;26(6):758 763. (10.1016/j.clnu.2007.04.007) 17719703

[111] Lariño-Noia J Lindkvist B Iglesias-García J Seijo-Ríos S Iglesias-Canle J Domínguez-Muñoz JE . Early and/or immediately full caloric diet versus standard refeeding in mild acute pancreatitis: a randomized open-label trial. Pancreatology. 2014;14(3):167 173. (10.1016/j.pan.2014.02.008) 24854611

[112] Ramírez-Maldonado E López Gordo SL Pueyo EM , et al. Immediate oral refeeding in patients with mild and moderate acute pancreatitis: a multicenter, randomized controlled trial (PADI trial). Ann Surg. 2021;274(2):255 263. (10.1097/SLA.0000000000004596) 33196485

[113] Arvanitakis M Ockenga J Bezmarevic M , et al. ESPEN guideline on clinical nutrition in acute and chronic pancreatitis. Clin Nutr. 2020;39(3):612 631. (10.1016/j.clnu.2020.01.004) 32008871

[114] Eckerwall GE Axelsson JB Andersson RG . Early nasogastric feeding in predicted severe acute pancreatitis: a clinical, randomized study. Ann Surg. 2006;244(6):959 965; discussion 965 967. (10.1097/01.sla.0000246866.01930.58) 17122621 PMC1856625

[115] Abou-Assi S Craig K O’Keefe SJD . Hypocaloric jejunal feeding is better than total parenteral nutrition in acute pancreatitis: results of a randomized comparative study. Am J Gastroenterol. 2002;97(9):2255 2262. (10.1111/j.1572-0241.2002.05979.x) 12358242

[116] Kalfarentzos F Kehagias J Mead N Kokkinis K Gogos CA . Enteral nutrition is superior to parenteral nutrition in severe acute pancreatitis: results of a randomized prospective trial. Br J Surg. 1997;84(12):1665 1669. 9448611

[117] Marik PE Zaloga GP . Meta-analysis of parenteral nutrition versus enteral nutrition in patients with acute pancreatitis. BMJ. 2004;328(7453):1407. (10.1136/bmj.38118.593900.55) 15175229 PMC421778

[118] Louie BE Noseworthy T Hailey D Gramlich LM Jacobs P Warnock GL . 2004 MacLean-Mueller prize enteral or parenteral nutrition for severe pancreatitis: a randomized controlled trial and health technology assessment. Can J Surg. 2005;48(4):298 306. 16149365 PMC3211537

[119] Petrov MS Kukosh MV Emelyanov NV . A randomized controlled trial of enteral versus parenteral feeding in patients with predicted severe acute pancreatitis shows a significant reduction in mortality and in infected pancreatic complications with total enteral nutrition. Dig Surg. 2006;23(5-6):336 344; discussion 344 345. (10.1159/000097949) 17164546

[120] Mora J Casas M Cardona D Farré A . Effect of enteral versus parenteral nutrition on inflammatory markers in severe acute pancreatitis. Pancreas. 2007;35(3):292. (10.1097/MPA.0b013e31805ba3c6) 17895857

[121] Doley RP Yadav TD Wig JD , et al. Enteral nutrition in severe acute pancreatitis. JOP. 2009;10(2):157 162. 19287109

[122] Kumar A Singh N Prakash S Saraya A Joshi YK . Early enteral nutrition in severe acute pancreatitis: a prospective randomized controlled trial comparing nasojejunal and nasogastric routes. J Clin Gastroenterol. 2006;40(5):431 434. (10.1097/00004836-200605000-00013) 16721226

[123] Qi D Yu B Huang J Peng M . Meta-analysis of early enteral nutrition provided within 24 hours of admission on clinical outcomes in acute pancreatitis. JPEN J Parenter Enter Nutr. 2018;42(7):1139 1147. (10.1002/jpen.1139) 29377204

[124] Bakker OJ van Brunschot S Farre A , et al. Timing of enteral nutrition in acute pancreatitis: meta-analysis of individuals using a single-arm of randomised trials. Pancreatology. 2014;14(5):340 346. (10.1016/j.pan.2014.07.008) 25128270

[125] Li X Ma F Jia K . Early enteral nutrition within 24 hours or between 24 and 72 hours for acute pancreatitis: evidence based on 12 RCTs. Med Sci Monit. 2014;20:2327 2335. (10.12659/MSM.892770) 25399541 PMC4247233

[126] Song J Zhong Y Lu X , et al. Enteral nutrition provided within 48 hours after admission in severe acute pancreatitis: a systematic review and meta-analysis. Med (Baltim). 2018;97(34):e11871. (10.1097/MD.0000000000011871) PMC6112989 30142782

[127] Petrov MS Pylypchuk RD Uchugina AF . A systematic review on the timing of artificial nutrition in acute pancreatitis. Br J Nutr. 2009;101(6):787 793. (10.1017/S0007114508123443) 19017421

[128] Feng P He C Liao G Chen Y . Early enteral nutrition versus delayed enteral nutrition in acute pancreatitis: a PRISMA-compliant systematic review and meta-analysis. Med (Baltim). 2017;96(46):e8648. (10.1097/MD.0000000000008648) PMC5704836 29145291

[129] Li P Jian JN Chen RL . Effect of early enteral nutrition on serum inflammatory factors and intestinal mucosal permeability in patients with severe acute pancreatitis. Turk J Gastroenterol. 2021;32(10):907 912. (10.5152/tjg.2021.201033) 34787096 PMC8975450

[130] Chang YS Fu HQ Xiao YM Liu JC . Nasogastric or nasojejunal feeding in predicted severe acute pancreatitis: a meta-analysis. Crit Care. 2013;17(3):R118. (10.1186/cc12790) 23786708 PMC4057382

[131] Singh N Sharma B Sharma M , et al. Evaluation of early enteral feeding through nasogastric and nasojejunal tube in severe acute pancreatitis: a noninferiority randomized controlled trial. Pancreas. 2012;41(1):153 159. (10.1097/MPA.0b013e318221c4a8) 21775915

[132] Eatock FC Chong P Menezes N , et al. A randomized study of early nasogastric versus nasojejunal feeding in severe acute pancreatitis. Am J Gastroenterol. 2005;100(2):432 439. (10.1111/j.1572-0241.2005.40587.x) 15667504

[133] Tiengou LE Gloro R Pouzoulet J , et al. Semi-elemental formula or polymeric formula: is there a better choice for enteral nutrition in acute pancreatitis? Randomized comparative study. JPEN J Parenter Enter Nutr. 2006;30(1):1 5. (10.1177/014860710603000101) 16387891

[134] Lakananurak N Gramlich L . Nutrition management in acute pancreatitis: clinical practice consideration. World J Clin Cases. 2020;8(9):1561 1573. (10.12998/wjcc.v8.i9.1561) 32432134 PMC7211526

[135] Jeurnink SM Nijs MM Prins HAB Greving JP Siersema PD . Antioxidants as a treatment for acute pancreatitis: a meta-analysis. Pancreatology. 2015;15(3):203 208. (10.1016/j.pan.2015.03.009) 25891791

[136] Asrani V Chang WK Dong Z Hardy G Windsor JA Petrov MS . Glutamine supplementation in acute pancreatitis: a meta-analysis of randomized controlled trials. Pancreatology. 2013;13(5):468 474. (10.1016/j.pan.2013.07.282) 24075510

[137] Yong L Lu QP Liu SH Fan H . Efficacy of glutamine-enriched nutrition support for patients with severe acute pancreatitis: a meta-analysis. JPEN J Parenter Enter Nutr. 2016;40(1):83 94. (10.1177/0148607115570391) 25655622

[138] Liu X Sun XF Ge QX . The role of glutamine supplemented total parenteral nutrition (TPN) in severe acute pancreatitis. Eur Rev Med Pharmacol Sci. 2016;20(19):4176 4180. 27775778

[139] Fuentes-Orozco C Cervantes-Guevara G Muciño-Hernández I , et al. L-alanyl-L-glutamine-supplemented parenteral nutrition decreases infectious morbidity rate in patients with severe acute pancreatitis. JPEN J Parenter Enter Nutr. 2008;32(4):403 411. (10.1177/0148607108319797) 18596311

[140] Sahin H Mercanlıgil SM İnanç N Ok E . Effects of glutamine-enriched total parenteral nutrition on acute pancreatitis. Eur J Clin Nutr. 2007;61(12):1429 1434. (10.1038/sj.ejcn.1602664) 17311061

[141] Gou S Yang Z Liu T Wu H Wang C . Use of probiotics in the treatment of severe acute pancreatitis: a systematic review and meta-analysis of randomized controlled trials. Crit Care. 2014;18(2):R57. (10.1186/cc13809) 24684832 PMC4056604

[142] Besselink MGH van Santvoort HC van der Heijden GJMG Buskens E Gooszen HG , Dutch Acute Pancreatitis Study Group. New randomized trial of probiotics in pancreatitis needed? Caution advised. Langenbecks Arch Surg. 2009;394(1):191 2; author reply 193. (10.1007/s00423-008-0419-y) 18841384

[143] Ukai T Shikata S Inoue M , et al. Early prophylactic antibiotics administration for acute necrotizing pancreatitis: a meta-analysis of randomized controlled trials. J Hepatobil Pancreat Sci. 2015;22(4):316 321. (10.1002/jhbp.221) 25678060

[144] Mazaki T Ishii Y Takayama T . Meta-analysis of prophylactic antibiotic use in acute necrotizing pancreatitis. Br J Surg. 2006;93(6):674 684. (10.1002/bjs.5389) 16703633

[145] Mourad MM Evans R Kalidindi V Navaratnam R Dvorkin L Bramhall SR . Prophylactic antibiotics in acute pancreatitis: endless debate. Ann R Coll Surg Engl. 2017;99(2):107 112. (10.1308/rcsann.2016.0355) 27917667 PMC5392851

[146] Ding N Sun YH Wen LM , et al. Assessment of prophylactic antibiotics administration for acute pancreatitis: a meta-analysis of randomized controlled trials. Chin Med J (Engl). 2020;133(2):212 220. (10.1097/CM9.0000000000000603) 31929369 PMC7028185

[147] Jiang K Huang W Yang XN Xia Q . Present and future of prophylactic antibiotics for severe acute pancreatitis. World J Gastroenterol. 2012;18(3):279 284. (10.3748/wjg.v18.i3.279) 22294832 PMC3261546

[148] Lim CLL Lee W Liew YX Tang SSL Chlebicki MP Kwa ALH . Role of antibiotic prophylaxis in necrotizing pancreatitis: a meta-analysis. J Gastrointest Surg. 2015;19(3):480 491. (10.1007/s11605-014-2662-6) 25608671

[149] Guo D Dai W Shen J , et al. Assessment of prophylactic carbapenem antibiotics administration for severe acute pancreatitis: an updated systematic review and meta-analysis. Digestion. 2022;103(3):183 191. (10.1159/000520892) 35026770

[150] Jafri NS Mahid SS Idstein SR Hornung CA Galandiuk S . Antibiotic prophylaxis is not protective in severe acute pancreatitis: a systematic review and meta-analysis. Am J Surg. 2009;197(6):806 813. (10.1016/j.amjsurg.2008.08.016) 19217608

[b150-tjg-35-S1-s1] Wittau M Mayer B Scheele J Henne-Bruns D Dellinger EP Isenmann R . Systematic review and meta-analysis of antibiotic prophylaxis in severe acute pancreatitis. Scand J Gastroenterol. 2011;46(3):261 270. (10.3109/00365521.2010.531486) 21067283

[b151-tjg-35-S1-s1] Villatoro E Mulla M Larvin M . Antibiotic therapy for prophylaxis against infection of pancreatic necrosis in acute pancreatitis. Cochrane Database Syst Rev. 2010;2010(5):CD002941. (10.1002/14651858.CD002941.pub3) 20464721 PMC7138080

[153] Bai Y Gao J Zou DW Li ZS . Prophylactic antibiotics cannot reduce infected pancreatic necrosis and mortality in acute necrotizing pancreatitis: evidence from a meta-analysis of randomized controlled trials. Am J Gastroenterol. 2008;103(1):104 110. (10.1111/j.1572-0241.2007.01575.x) 17925000

[154] Manes G Uomo I Menchise A Rabitti PG Ferrara EC Uomo G . Timing of antibiotic prophylaxis in acute pancreatitis: a controlled randomized study with meropenem. Am J Gastroenterol. 2006;101(6):1348 1353. (10.1111/j.1572-0241.2006.00567.x) 16771960

[155] Banks PA Freeman ML , Practice Parameters Committee of the American College of Gastroenterology. Practice guidelines in acute pancreatitis. Am J Gastroenterol. 2006;101(10):2379 2400. (10.1111/j.1572-0241.2006.00856.x) 17032204

[156] Beger HG Bittner R Block S Büchler M . Bacterial contamination of pancreatic necrosis: a prospective clinical study. Gastroenterology. 1986;91(2):433 438. (10.1016/0016-5085(86)90579-2) 3522342

[157] Bradley EL 3rd Allen K . A prospective longitudinal study of observation versus surgical intervention in the management of necrotizing pancreatitis. Am J Surg. 1991;161(1):19 24; discussion 24. (10.1016/0002-9610(91)90355-h) 1987854

[158] Besselink MG van Santvoort HC Boermeester MA , et al. Timing and impact of infections in acute pancreatitis. Br J Surg. 2009;96(3):267 273. (10.1002/bjs.6447) 19125434

[159] Trikudanathan G Navaneethan U Vege SS . Intra-abdominal fungal infections complicating acute pancreatitis: a review. Am J Gastroenterol. 2011;106(7):1188 1192. (10.1038/ajg.2010.497) 21731015

[160] Isenmann R Rünzi M Kron M , et al. Prophylactic antibiotic treatment in patients with predicted severe acute pancreatitis: a placebo-controlled, double-blind trial. Gastroenterology. 2004;126(4):997 1004. (10.1053/j.gastro.2003.12.050) 15057739

[b160-tjg-35-S1-s1] García-Barrasa A Borobia FG Pallares R , et al. A double-blind, placebo-controlled trial of ciprofloxacin prophylaxis in patients with acute necrotizing pancreatitis. J Gastrointest Surg. 2009;13(4):768 774. (10.1007/s11605-008-0773-7) 19082671

[b161-tjg-35-S1-s1] Dellinger EP Tellado JM Soto NE , et al. Early antibiotic treatment for severe acute necrotizing pancreatitis: a randomized, double-blind, placebo-controlled study. Ann Surg. 2007;245(5):674 683. (10.1097/01.sla.0000250414.09255.84) 17457158 PMC1877078

[b162-tjg-35-S1-s1] Nordback I Sand J Saaristo R Paajanen H . Early treatment with antibiotics reduces the need for surgery in acute necrotizing pancreatitis--a single-center randomized study. J Gastrointest Surg. 2001;5(2):113 118; discussion 118 120. (10.1016/s1091-255x(01)80021-4) 11331472

[b163-tjg-35-S1-s1] Xue P Deng LH Zhang ZD , et al. Effect of antibiotic prophylaxis on acute necrotizing pancreatitis: results of a randomized controlled trial. J Gastroenterol Hepatol. 2009;24(5):736 742. (10.1111/j.1440-1746.2008.05758.x) 19220676

[165] Poropat G Radovan A Peric M , et al. Prevention of infectious complications in acute pancreatitis: results of a single-center, randomized, controlled trial. Pancreas. 2019;48(8):1056 1060. (10.1097/MPA.0000000000001368) 31404018

[166] Iqbal U Khara HS Hu Y , et al. Emergent versus urgent ERCP in acute cholangitis: a systematic review and meta-analysis. Gastrointest Endosc. 2020;91(4):753 760.e4. (10.1016/j.gie.2019.09.040) 31628955

[167] Tse F Yuan Y . Early routine endoscopic retrograde cholangiopancreatography strategy versus early conservative management strategy in acute gallstone pancreatitis. Cochrane Database Syst Rev. 2012;2012(5):CD009779. (10.1002/14651858.CD009779.pub2) 22592743 PMC11491195

[168] Mukai S Itoi T Tsuchiya T , et al. Urgent and emergency endoscopic retrograde cholangiopancreatography for gallstone-induced acute cholangitis and pancreatitis. Dig Endosc. 2023;35(1):47 57. (10.1111/den.14379) 35702927

[169] Kiriyama S Kozaka K Takada T , et al. Tokyo Guidelines 2018: diagnostic criteria and severity grading of acute cholangitis (with videos). J Hepatobil Pancreat Sci. 2018;25(1):17 30. (10.1002/jhbp.512) 29032610

[170] Shrestha DB Budhathoki P Sedhai YR , et al. Urgent endoscopic retrograde cholangiopancreatography (ERCP) vs. conventional Approach in Acute Biliary Pancreatitis without cholangitis: an updated systematic review and meta-analysis. Cureus. 2022;14(1):e21342. (10.7759/cureus.21342) 35198265 PMC8852244

[171] Meeralam Y Al-Shammari K Yaghoobi M . Diagnostic accuracy of EUS compared with MRCP in detecting choledocholithiasis: a meta-analysis of diagnostic test accuracy in head-to-head studies. Gastrointest Endosc. 2017;86(6):986 993. (10.1016/j.gie.2017.06.009) 28645544

[172] Lebenson J Oliver T . Chapter 16 Rodrigo L. , ed. Hypertriglyceride Induced Acute Pancreatitis. Acute Pancreatitis; January 27th, 2011 Published: January 18th, 2012:259 278. (10.5772/25581)

[173] Pulipati VP Amblee A Yap SET Shaka H Tahsin B Fogelfeld L . Hypertriglyceridemia-associated acute pancreatitis: response to continuous insulin infusion. PLoS One. 2021;30(11):e0260495. (10.1371/journal.pone.0260495) PMC8631651 34847179

[174] Li J Chen TR Gong HL Wan MH Chen GY Tang WF . Intensive insulin therapy in severe acute pancreatitis: a meta-analysis and systematic review. West Indian Med J. 2012;61(6):574 579. 23441350

[175] Dhindsa S Sharma A Al-Khazaali A , et al. Intravenous insulin versus conservative management in hypertriglyceridemia-associated acute pancreatitis. J Endocr Soc. 2020;4(1):bvz019. (10.1210/jendso/bvz019) 31993551 PMC6977947

[176] Garg R Rustagi T . Management of hypertriglyceridemia induced acute pancreatitis. BioMed Res Int. 2018;2018:4721357. (10.1155/2018/4721357) 30148167 PMC6083537

[177] Altinkaya E Aktas A . Insulin and heparin therapies in acute pancreatitis due to hypertriglyceridemia. J Coll Physicians Surg Pak. 2021;31(11):1337 1340. (10.29271/jcpsp.2021.11.1337) 34689493

[178] Yan LH Hu XH Chen RX , et al. Plasmapheresis compared with conventional treatment for hypertriglyceridemia-induced acute pancreatitis: a systematic review and meta-analysis. J Clin Apher. 2023;38(1):4 15. (10.1002/jca.22018) 36151902

[179] Zhang Y Lin J Wu L Lin J Liang Y . Blood purification for hypertriglyceridemia-induced acute pancreatitis: a meta-analysis. Pancreas. 2022;51(5):531 539. (10.1097/MPA.0000000000002071) 35858225

[180] Kaner EFS Beyer FR Muirhead C , et al. Effectiveness of brief alcohol interventions in primary care populations. Cochrane Database Syst Rev. 2018;2(2):CD004148. (10.1002/14651858.CD004148.pub4) PMC6491186 29476653

[181] Babor TF . Avoiding the horrid and beastly sin of drunkenness: does dissuasion make a difference? J Consult Clin Psychol. 1994;62(6):1127 1140. (10.1037//0022-006x.62.6.1127) 7860811

[182] Kaner EF Beyer F Dickinson HO , et al. Effectiveness of brief alcohol interventions in primary care populations. Cochrane Database Syst Rev. 2007;(2)(2):CD004148. (10.1002/14651858.CD004148.pub3) 17443541

[183] Mdege ND Fayter D Watson JM Stirk L Sowden A Godfrey C . Interventions for reducing alcohol consumption among general hospital inpatient heavy alcohol users: a systematic review. Drug Alcohol Depend. 2013;131(1-2):1 22. (10.1016/j.drugalcdep.2013.01.023) 23474201

[184] Nordback I Pelli H Lappalainen-Lehto R Järvinen S Räty S Sand J . The recurrence of acute alcohol-associated pancreatitis can be reduced: a randomized controlled trial. Gastroenterology. 2009;136(3):848 855. (10.1053/j.gastro.2008.11.044) 19162029

[185] Badalov N Baradarian R Iswara K Li J Steinberg W Tenner S . Drug-induced acute pancreatitis: an evidence-based review. Clin Gastroenterol Hepatol. 2007;5(6):648 661; quiz 644. (10.1016/j.cgh.2006.11.023) 17395548

[186] Nitsche C Maertin S Scheiber J Ritter CA Lerch MM Mayerle J . Drug-induced pancreatitis. Curr Gastroenterol Rep. 2012;14(2):131 138. (10.1007/s11894-012-0245-9) 22314811

[187] Zimmerman HJ . Hepatoxicity. The Adverse Effects of Drugs and Other Chemicals on the Liver; 91-95. New York: Appleton-Century-Crofts; 1978.

[188] Meczker Á Hanák L Párniczky A , et al. Analysis of 1060 cases of drug-induced acute pancreatitis. Gastroenterology. 2020;159(5):1958 1961.e8. (10.1053/j.gastro.2020.07.016) 32687926

[189] Fathallah N Slim R Larif S , et al. Drug-induced acute pancreatitis confirmed by positive re-challenge. Pancreat Disord. 2005. (10.4172/2165-7092.S6-005)

[190] Yokoe M Takada T Mayumi T , et al. Japanese guidelines for the management of acute pancreatitis: Japanese Guidelines 2015. J Hepato-Bil Pancreat Sci. 2015;22(6):405 432. (10.1002/jhbp.259) 25973947

[191] Harshit Kumar A Singh Griwan M . A comparison of Apache II, BISAP, Ranson’s score and modified CTSI in predicting the severity of acute pancreatitis based on the 2012 revised Atlanta Classification. Gastroenterol Rep (Oxf). 2018;6(2):127 131. (10.1093/gastro/gox029) 29780601 PMC5952961

[192] James TW Crockett SD . Management of acute pancreatitis in the first 72 hours. Curr Opin Gastroenterol. 2018;34(5):330 335. (10.1097/MOG.0000000000000456) 29957661 PMC6245573

[193] Badal BD Kruger AJ Hart PA , et al. Predictors of hospital transfer and associated risks of mortality in acute pancreatitis. Pancreatology. 2021;21(1):25 30. (10.1016/j.pan.2020.12.001) 33341342

[194] Zerem E . Treatment of severe acute pancreatitis and its complications. World J Gastroenterol. 2014;20(38):13879 13892. (10.3748/wjg.v20.i38.13879) 25320523 PMC4194569

[195] Mihoc T Tarta C Duta C , et al. Monitoring approach of fatality risk factors for patients with severe acute pancreatitis admitted to the Intensive Care Unit. A retrospective, monocentric study. Diagnostics (Basel). 2021;11(11):2013. (10.3390/diagnostics11112013) 34829360 PMC8625923

[196] Thoeni RF . The revised Atlanta classification of acute pancreatitis: its importance for the radiologist and its effect on treatment. Radiology. 2012;262(3):751 764. (10.1148/radiol.11110947) 22357880

[197] Lenhart DK Balthazar EJ . MDCT of acute mild (nonnecrotizing) pancreatitis: abdominal complications and fate of fluid collections. AJR Am J Roentgenol. 2008;190(3):643 649. (10.2214/AJR.07.2761) 18287434

[198] Sheu Y Furlan A Almusa O Papachristou G Bae KT . The revised Atlanta classification for acute pancreatitis: a CT imaging guide for radiologists. Emerg Radiol. 2012;19(3):237 243. (10.1007/s10140-011-1001-4) 22160496

[b198-tjg-35-S1-s1] Bollen TL . Imaging of acute pancreatitis: update of the revised Atlanta classification. Radiol Clin North Am. 2012;50(3):429 445. (10.1016/j.rcl.2012.03.015) 22560690

[200] Kapoor K Banks PA . Early prognostic evaluation of acute pancreatitis: a non-going challenge. J Pancreas. 2013;14(2):109 111. (10.6092/1590-8577/1405) 23474548

[201] Pan G Wan MH Xie KL , et al. Classification and management of pancreatic pseudocysts. Med (Baltim). 2015;94(24):e960. (10.1097/MD.0000000000000960) PMC4616556 26091462

[b201-tjg-35-S1-s1] Pereira F Caldeira A Leite S , et al. GRUPUGE perspective: endoscopic ultrasound-guided drainage of peripancreatic collections. GE Port J Gastroenterol. 2020;28(1):39 51. (10.1159/000509193) 33564703 PMC7841806

[203] Brun A Agarwal N Pitchumoni CS . Fluid collections in and around the pancreas in acute pancreatitis. J Clin Gastroenterol. 2011;45(7):614 625. (10.1097/MCG.0b013e318213ef3e) 21750432

[204] Poornachandra KS Bhasin DK Nagi B , et al. Clinical, biochemical, and radiologic parameters at admission predicting formation of a pseudocyst in acute pancreatitis. J Clin Gastroenterol. 2011;45(2):159 163. (10.1097/MCG.0b013e3181dd9d14) 20628310

[205] Tyberg A Karia K Gabr M , et al. Management of pancreatic fluid collections: a comprehensive review of the literature. World J Gastroenterol. 2016;22(7):2256 2270. (10.3748/wjg.v22.i7.2256) 26900288 PMC4735000

[206] Trikudanathan G Wolbrink DRJ van Santvoort HC Mallery S Freeman M Besselink MG . Current concepts in severe acute and necrotizing pancreatitis: an evidence-based approach. Gastroenterology. 2019;156(7):1994 2007.e3. (10.1053/j.gastro.2019.01.269) 30776347

[207] Crockett SD Wani S Gardner TB Falck-Ytter Y Barkun AN , American Gastroenterological Association Institute Clinical Guidelines Committee. American Gastroenterological Association institute guideline on initial management of acute pancreatitis. Gastroenterology. 2018;154(4):1096 1101. (10.1053/j.gastro.2018.01.032) 29409760

[208] Wang M Wei A Guo Q , et al. Clinical outcomes of combined necrotizing pancreatitis versus extrapancreatic necrosis alone. Pancreatology. 2016;16(1):57 65. (10.1016/j.pan.2015.10.010) 26626203

[209] Thoeni RF . Imaging of acute pancreatitis. Radiol Clin North Am. 2015;53(6):1189 1208. (10.1016/j.rcl.2015.06.006) 26526433

[210] Arvanitakis M Dumonceau JM Albert J , et al. Endoscopic management of acute necrotizing pancreatitis: European Society of Gastrointestinal Endoscopy (ESGE) evidence-based multidisciplinary guidelines. Endoscopy. 2018;50(5):524 546. (10.1055/a-0588-5365) 29631305

[211] Dellinger EP Forsmark CE Layer P , et al. Determinant-based classification of acute pancreatitis severity: an international multidisciplinary consultation. Ann Surg. 2012;256(6):875 880. (10.1097/SLA.0b013e318256f778) 22735715

[212] Rana SS Chaudhary V Sharma R Sharma V Chhabra P Bhasin DK . Comparison of abdominal ultrasound, endoscopic ultrasound and magnetic resonance imaging in detection of necrotic debris in walled-off pancreatic necrosis. Gastroenterol Rep (Oxf). 2016;4(1):50 53. (10.1093/gastro/gou088) 25573950 PMC4760061

[213] Murphy KP O’Connor OJ Maher MM . Updated imaging nomenclature for acute pancreatitis. AJR Am J Roentgenol. 2014;203(5):W464 W469. (10.2214/AJR.13.12222) 25341160

[214] Baron TH DiMaio CJ Wang AY Morgan KA . American Gastroenterological Association clinical practice update: management of pancreatic necrosis. Gastroenterology. 2020;158(1):67 75.e1. (10.1053/j.gastro.2019.07.064) 31479658

[215] Vege SS Fletcher JG Talukdar R Sarr MG . Peripancreatic collections in acute pancreatitis: correlation between computerized tomography and operative findings. World J Gastroenterol. 2010;16(34):4291 4296. (10.3748/wjg.v16.i34.4291) 20818812 PMC2937109

[216] van Grinsven J van Brunschot S van Baal MC , et al. Natural history of gas configurations and encapsulation in necrotic collections during necrotizing pancreatitis. J Gastrointest Surg. 2018;22(9):1557 1564. (10.1007/s11605-018-3792-z) 29752684

[217] Oh CH Lee JK Song TJ , et al. Clinical practice guidelines for the endoscopic management of peripancreatic fluid collections. Clin Endosc. 2021;54(4):505 521. (10.5946/ce.2021.185) 34311518 PMC8357592

[218] Maldonado I Shetty A Estay MC , et al. Acute pancreatitis imaging in MDCT: state of the art of usual and unusual local complications. 2012 Atlanta classification revisited. Curr Probl Diagn Radiol. 2021;50(2):186 199. (10.1067/j.cpradiol.2020.04.002) 32553440

[219] Ali T Srinivasan N Le V Chimpiri AR Tierney WM . Pancreaticopleural fistula. Pancreas. 2009;38(1):e26 e31. (10.1097/MPA.0b013e3181870ad5) 19106743

[220] Larsen M Kozarek R . Management of pancreatic ductal leaks and fistulae. J Gastroenterol Hepatol. 2014;29(7):1360 1370. (10.1111/jgh.12574) 24650171

[221] Tan JH Zhou L Cao RC Zhang GW . Identification of risk factors for pancreatic pseudocysts formation, intervention and recurrence: a 15-year retrospective analysis in a tertiary hospital in China. BMC Gastroenterol. 2018;18(1):143. (10.1186/s12876-018-0874-z) 30285639 PMC6167814

[222] Umapathy C Gajendran M Mann R , et al. Pancreatic fluid collections: clinical manifestations, diagnostic evaluation and management. Dis Mon. 2020;66(11):100986. (10.1016/j.disamonth.2020.100986) 32312558

[223] American Society for Gastrointestinal Endoscopy Standards of Practice Committee, Muthusamy VR Chandrasekhara V , et al. The role of endoscopy in the diagnosis and treatment of inflammatory pancreatic fluid collections. Gastrointest Endosc. 2016;83(3):481 488. (10.1016/j.gie.2015.11.027) 26796695

[224] Cui ML Kim KH Kim HG , et al. Incidence, risk factors and clinical course of pancreatic fluid collections in acute pancreatitis. Dig Dis Sci. 2014;59(5):1055 1062. (10.1007/s10620-013-2967-4) 24326631

[225] Yeo CJ Bastidas JA Lynch-Nyhan A Fishman EK Zinner MJ Cameron JL . The natural history of pancreatic pseudocysts documented by computed tomography. Surg Gynecol Obstet. 1990;170(5):411 417. 2326721

[226] Cheruvu CVN Clarke MG Prentice M Eyre-Brook IA . Conservative treatment as an option in the management of pancreatic pseudocyst. Ann R Coll Surg Engl. 2003;85(5):313 316. (10.1308/003588403769162413) 14594534 PMC1964324

[227] Nguyen BL Thompson JS Edney JA Bragg LE Rikkers LF . Influence of the etiology of pancreatitis on the natural history of pancreatic pseudocysts. Am J Surg. 1991;162(6):527 30; discussion 531. (10.1016/0002-9610(91)90103-k) 1670219

[228] Rasch S Nötzel B Phillip V Lahmer T Schmid RM Algül H . Management of pancreatic pseudocysts-A retrospective analysis. PLoS One. 2017;12(9):e0184374. (10.1371/journal.pone.0184374) 28877270 PMC5587297

[229] Xiao NJ Cui TT Liu F Li W . Current status of treatments of pancreatic and peripancreatic collections of acute pancreatitis. World J Gastrointest Surg. 2021;13(7):633 644. (10.4240/wjgs.v13.i7.633) 34354797 PMC8316846

[b229-tjg-35-S1-s1] Bezmarević M van Dijk SM Voermans RP van Santvoort HC Besselink MG . Management of (peri)pancreatic collections in acute pancreatitis. Visc Med. 2019;35(2):91 96. (10.1159/000499631) 31192242 PMC6515122

[b230-tjg-35-S1-s1] Alali A Mosko J May G Teshima C . Endoscopic ultrasound-guided management of pancreatic fluid collections: update and review of the literature. Clin Endosc. 2017;50(2):117 125. (10.5946/ce.2017.045) 28391671 PMC5398360

[232] Goyal J Ramesh J . Endoscopic management of peripancreatic fluid collections. Frontline Gastroenterol. 2015;6(3):199 207. (10.1136/flgastro-2014-100444) 28839811 PMC5369570

[233] Aghdassi A Simon P Pickartz T Budde C Skube ME Lerch MM . Endoscopic management of complications of acute pancreatitis: an update on the field. Expert Rev Gastroenterol Hepatol. 2018;12(12):1207 1218. (10.1080/17474124.2018.1537781) 30791791 PMC11851565

[234] Tan JH Chin W Shaikh AL Zheng S . Pancreatic pseudocyst: Dilemma of its recent management (Review) [review]. Exp Ther Med. 2021;21(2):159. (10.3892/etm.2020.9590) 33456526 PMC7792492

[235] Chawla A Afridi F Prasath V Chokshi R Ahlawat S . Analysis of pancreatic pseudocyst drainage procedural outcomes: a population based study. Surg Endosc. 2023;37(1):156 164. (10.1007/s00464-022-09427-z) 35879571

[236] Akshintala VS Saxena P Zaheer A , et al. A comparative evaluation of outcomes of endoscopic versus percutaneous drainage for symptomatic pancreatic pseudocysts. Gastrointest Endosc. 2014;79(6):921 928. (10.1016/j.gie.2013.10.032) 24315454

[b236-tjg-35-S1-s1] Adams DB Harvey TS Anderson MC . Percutaneous catheter drainage of infected pancreatic and peripancreatic fluid collections. Arch Surg. 1990;125(12):1554 1557. (10.1001/archsurg.1990.01410240032006) 2244807

[b237-tjg-35-S1-s1] Adams DB Anderson MC . Percutaneous catheter drainage compared with internal drainage in the management of pancreatic pseudocyst. Ann Surg. 1992;215(6):571 6; discussion 576. (10.1097/00000658-199206000-00003) 1632678 PMC1242505

[239] Hookey LC Debroux S Delhaye M Arvanitakis M Le Moine O Devière J . Endoscopic drainage of pancreatic-fluid collections in 116 patients: a comparison of etiologies, drainage techniques, and outcomes. Gastrointest Endosc. 2006;63(4):635 643. (10.1016/j.gie.2005.06.028) 16564865

[240] Wan J Wu D He W , et al. Comparison of percutaneous vs endoscopic drainage in the management of pancreatic fluid collections: a prospective cohort study. J Gastroenterol Hepatol. 2020;35(12):2170 2175. (10.1111/jgh.15121) 32473080

[241] Cai QC Zhang YX Liao Y Gong JP Xiong B . Is endoscopic drainage better than percutaneous drainage for patients with pancreatic fluid collections? A comparative meta-analysis. Rev Esp Enferm Dig. 2021;113(6):454 462. (10.17235/reed.2020.7170/2020) 33267596

[242] Wang Y Omar YA Agrawal R Gong Z . Comparison of treatment modalities in pancreatic pseudocyst: a population based study. World J Gastrointest Surg. 2019;11(9):365 372. (10.4240/wjgs.v11.i9.365) 31572562 PMC6766474

[243] Morton JM Brown A Galanko JA Norton JA Grimm IS Behrns KE . A national comparison of surgical versus percutaneous drainage of pancreatic pseudocysts: 1997-2001. J Gastrointest Surg. 2005;9(1):15 20; discussion 20. (10.1016/j.gassur.2004.10.005) 15623440

[244] Saul A Ramirez Luna MA Chan C , et al. EUS-guided drainage of pancreatic pseudocysts offers similar success and complications compared to surgical treatment but with a lower cost. Surg Endosc. 2016;30(4):1459 1465. (10.1007/s00464-015-4351-2) 26139498

[b244-tjg-35-S1-s1] Teoh AYB Dhir V Jin ZD Kida M Seo DW Ho KY . Systematic review comparing endoscopic, percutaneous and surgical pancreatic pseudocyst drainage. World J Gastrointest Endosc. 2016;8(6):310 318. (10.4253/wjge.v8.i6.310) 27014427 PMC4804189

[b245-tjg-35-S1-s1] Varadarajulu S Bang JY Sutton BS Trevino JM Christein JD Wilcox CM . Equal efficacy of endoscopic and surgical cystogastrostomy for pancreatic pseudocyst drainage in a randomized trial. Gastroenterology. 2013;145(3):583 90.e1. (10.1053/j.gastro.2013.05.046) 23732774

[247] Zhao X Feng T Ji W . Endoscopic versus surgical treatment for pancreatic pseudocyst. Dig Endosc. 2016;28(1):83 91. (10.1111/den.12542) 26331472

[248] Farias GFA Bernardo WM De Moura DTH , et al. Endoscopic versus surgical treatment for pancreatic pseudocysts: systematic review and meta-analysis. Med (Baltim). 2019;98(8):e14255. (10.1097/MD.0000000000014255) PMC6407966 30813129

[249] Gurusamy KS Pallari E Hawkins N Pereira SP Davidson BR . Management strategies for pancreatic pseudocysts. Cochrane Database Syst Rev. 2016;4(4):CD011392. (10.1002/14651858.CD011392.pub2) 27075711 PMC6457582

[250] Varadarajulu S Wilcox CM Tamhane A Eloubeidi MA Blakely J Canon CL . Role of EUS in drainage of peripancreatic fluid collection not amenable for endoscopic transmural drainage. Gastrointest Endosc. 2007;66(6):1107 1119. (10.1016/j.gie.2007.03.1027) 17892874

[251] Fockens P Johnson TG van Dullemen HM Huibregtse K Tytgat GN . Endosonographic imaging of pancreatic pseudocysts before endoscopic transmural drainage. Gastrointest Endosc. 1997;46(5):412 416. (10.1016/s0016-5107(97)70033-6) 9402114

[252] Kahaleh M Shami VM Conaway MR , et al. Endoscopic ultrasound drainage of pancreatic pseudocyst: a prospective comparison with conventional endoscopic drainage. Endoscopy. 2006;38(4):355 359. (10.1055/s-2006-925249) 16680634

[253] Varadarajulu S Christein JD Tamhane A Drelichman ER Wilcox CM . Prospective randomized trial comparing EUS and EGD for transmural drainage of pancreatic pseudocysts (with videos). Gastrointest Endosc. 2008;68(6):1102 1111. (10.1016/j.gie.2008.04.028) 18640677

[254] Park DH Lee SS Moon SH , et al. Endoscopic ultrasound-guided versus conventional transmural drainage for pancreatic pseudocysts: a prospective randomized trial. Endoscopy. 2009;41(10):842 848. (10.1055/s-0029-1215133) 19798610

[255] Barthet M Lamblin G Gasmi M Vitton V Desjeux A Grimaud JC . Clinical usefulness of a treatment algorithm for pancreatic pseudocysts. Gastrointest Endosc. 2008;67(2):245 252. (10.1016/j.gie.2007.06.014) 18226686

[256] Johnson MD Walsh RM Henderson JM , et al. Surgical versus nonsurgical management of pancreatic pseudocysts. J Clin Gastroenterol. 2009;43(6):586 590. (10.1097/MCG.0b013e31817440be) 19077728

[257] Jagielski M Smoczyński M Szeliga J Adrych K Jackowski M . Various endoscopic techniques for treatment of consequences of acute necrotizing pancreatitis: practical updates for the endoscopist. J Clin Med. 2020;9(1):117. (10.3390/jcm9010117) 31906294 PMC7019492

[258] Jagielski M Jackowski M . The role of endoscopic transpapillary stenting of the main pancreatic duct during the endoscopic treatment of pancreatic fluid collections. J Clin Med. 2021;10(4):761. (10.3390/jcm10040761) 33672814 PMC7918499

[259] Baron TH . Endoscopic drainage of pancreatic fluid collections and pancreatic necrosis. Gastrointest Endosc Clin N Am. 2003;13(4):743 764. (10.1016/s1052-5157(03)00100-4) 14986796

[260] Samuelson AL Shah RJ . Endoscopic management of pancreatic pseudocysts. Gastroenterol Clin North Am. 2012;41(1):47 62. (10.1016/j.gtc.2011.12.007) 22341249

[261] Ge PS Weizmann M Watson RR . Pancreatic pseudocysts: advances in endoscopic management. Gastroenterol Clin North Am. 2016;45(1):9 27. (10.1016/j.gtc.2015.10.003) 26895678

[262] Telford JJ Farrell JJ Saltzman JR , et al. Pancreatic stent placement for duct disruption. Gastrointest Endosc. 2002;56(1):18 24. (10.1067/mge.2002.125107) 12085030

[263] Trevino JM Tamhane A Varadarajulu S . Successful stenting in ductal disruption favorably impacts treatment outcomes in patients undergoing transmural drainage of peripancreatic fluid collections. J Gastroenterol Hepatol. 2010;25(3):526 531. (10.1111/j.1440-1746.2009.06109.x) 20074158

[264] Shrode CW Macdonough P Gaidhane M , et al. Multimodality endoscopic treatment of pancreatic duct disruption with stenting and pseudocyst drainage: how efficacious is it? Dig Liver Dis. 2013;45(2):129 133. (10.1016/j.dld.2012.08.026) 23036185

[265] Varadarajulu S Noone TC Tutuian R Hawes RH Cotton PB . Predictors of outcome in pancreatic duct disruption managed by endoscopic transpapillary stent placement. Gastrointest Endosc. 2005;61(4):568 575. (10.1016/s0016-5107(04)02832-9) 15812410

[266] Yang D Amin S Gonzalez S , et al. Transpapillary drainage has no added benefit on treatment outcomes in patients undergoing EUS-guided transmural drainage of pancreatic pseudocysts: a large multicenter study. Gastrointest Endosc. 2016;83(4):720 729. (10.1016/j.gie.2015.10.040) 26548849

[267] Varadarajulu S Bang JY Phadnis MA Christein JD Wilcox CM . Endoscopic transmural drainage of peripancreatic fluid collections: outcomes and predictors of treatment success in 211 consecutive patients. J Gastrointest Surg. 2011;15(11):2080 2088. (10.1007/s11605-011-1621-8) 21786063

[268] Amin S Yang DJ Lucas AL Gonzalez S DiMaio CJ . There is no advantage to transpapillary pancreatic duct stenting for the transmural endoscopic drainage of pancreatic fluid collections: a meta-analysis. Clin Endosc. 2017;50(4):388 394. (10.5946/ce.2016.091) 28415167 PMC5565042

[269] van Baal MC Bollen TL Bakker OJ , et al. The role of routine fine-needle aspiration in the diagnosis of infected necrotizing pancreatitis. Surgery. 2014;155(3):442 448. (10.1016/j.surg.2013.10.001) 24287142

[b269-tjg-35-S1-s1] Madenci AL Michailidou M Chiou G Thabet A Fernández-del Castillo C Fagenholz PJ . A contemporary series of patients undergoing open debridement for necrotizing pancreatitis. Am J Surg. 2014;208(3):324 331. (10.1016/j.amjsurg.2013.11.004) 24767969

[271] Islim F Salik AE Bayramoglu S Guven K Alis H Turhan AN . Non-invasive detection of infection in acute pancreatic and acute necrotic collections with diffusion-weighted magnetic resonance imaging: preliminary findings. Abdom Imaging. 2014;39(3):472 481. (10.1007/s00261-014-0076-2) 24441591

[272] Borens B Arvanitakis M Absil J , et al. Added value of diffusion-weighted magnetic resonance imaging for the detection of pancreatic fluid collection infection. Eur Radiol. 2017;27(3):1064 1073. (10.1007/s00330-016-4462-8) 27300193

[273] Yang CJ Chen J Phillips ARJ Windsor JA Petrov MS . Predictors of severe and critical acute pancreatitis: a systematic review. Dig Liver Dis. 2014;46(5):446 451. (10.1016/j.dld.2014.01.158) 24646880

[274] Rau BM Kemppainen EA Gumbs AA , et al. Early assessment of pancreatic infections and overall prognosis in severe acute pancreatitis by procalcitonin (PCT): a prospective international multicenter study. Ann Surg. 2007;245(5):745 754. (10.1097/01.sla.0000252443.22360.46) 17457167 PMC1877072

[275] Besselink MGH Verwer TJ Schoenmaeckers EJP , et al. Timing of surgical intervention in necrotizing pancreatitis. Arch Surg. 2007;142(12):1194 1201. (10.1001/archsurg.142.12.1194) 18086987

[b275-tjg-35-S1-s1] Dong X Gao SL Xie QP Xu L Xu YL Wu YL . In situ high-volume modified continuous closed and/or open lavage for infected necrotizing pancreatitis. Pancreas. 2008;36(1):44 49. (10.1097/mpa.0b013e31812e9688) 18192880

[277] Rau B Bothe A Beger HG . Surgical treatment of necrotizing pancreatitis by necrosectomy and closed lavage: changing patient characteristics and outcome in a 19-year, single-center series. Surgery. 2005;138(1):28 39. (10.1016/j.surg.2005.03.010) 16003313

[278] Rodriguez JR Razo AO Targarona J , et al. Debridement and closed packing for sterile or infected necrotizing pancreatitis: insights into indications and outcomes in 167 patients. Ann Surg. 2008;247(2):294 299. (10.1097/SLA.0b013e31815b6976) 18216536 PMC3806106

[279] Boxhoorn L van Dijk SM van Grinsven J , et al. Immediate versus postponed intervention for infected necrotizing pancreatitis. N Engl J Med. 2021;385(15):1372 1381. (10.1056/NEJMoa2100826) 34614330

[280] Zhang H Gao L Mao WJ , et al. Early versus delayed intervention in necrotizing acute pancreatitis complicated by persistent organ failure. Hepatobiliary Pancreat Dis Int. 2022;21(1):63 68. (10.1016/j.hbpd.2020.12.019) 33478932

[281] Lu J Cao F Zheng Z , et al. How to identify the indications for early intervention in acute necrotizing pancreatitis patients: a long-term follow-up study. Front Surg. 2022;9:842016. (10.3389/fsurg.2022.842016) 35465437 PMC9019048

[282] van Grinsven J van Santvoort HC Boermeester MA , et al. Timing of catheter drainage in infected necrotizing pancreatitis. Nat Rev Gastroenterol Hepatol. 2016;13(5):306 312. (10.1038/nrgastro.2016.23) 26956064

[283] Gao L Zhang H Li G , et al. The clinical outcome from early versus delayed minimally invasive intervention for infected pancreatic necrosis: a systematic review and meta-analysis. J Gastroenterol. 2022;57(6):397 406. (10.1007/s00535-022-01876-6) 35488104

[284] Albers D Meining A Hann A Ayoub YK Schumacher B . Direct endoscopic necrosectomy in infected pancreatic necrosis using lumen-apposing metal stents: early intervention does not compromise outcome. Endosc Int Open. 2021;9(3):E490 E495. (10.1055/a-1341-0654) 33655055 PMC7899788

[b284-tjg-35-S1-s1] Ke L Dong X Chen T , et al. Early on-demand drainage or standard management for acute pancreatitis patients with acute necrotic collections and persistent organ failure: a pilot randomized controlled trial. J Hepatobil Pancreat Sci. 2021;28(4):387 396. (10.1002/jhbp.915) 33595879

[286] Dost W Qasemi F Ali W , et al. Immediate catheter drainage versus delayed drainage in the management of infected necrotizing pancreatitis. Cureus. 2022;14(7):e26485. (10.7759/cureus.26485) 35919210 PMC9339159

[287] Guo Q Li A Xia Q , et al. Timing of intervention in necrotizing pancreatitis. J Gastrointest Surg. 2014;18(10):1770 1776. (10.1007/s11605-014-2606-1) 25091844

[288] Moran RA Halloran C Guo Q , et al. Early infection is an independent risk factor for increased mortality in patients with culture-confirmed infected pancreatic necrosis. Pancreatology. 2022;22(1):67 73. (10.1016/j.pan.2021.11.003) 34774414

[289] Rana SS Sharma RK Gupta P Gupta R . Natural course of asymptomatic walled off pancreatic necrosis. Dig Liver Dis. 2019;51(5):730 734. (10.1016/j.dld.2018.10.010) 30467075

[b289-tjg-35-S1-s1] Jagielski M Smoczyński M Studniarek M Adrych K . Spontaneous regression of asymptomatic walled-off pancreatic necrosis. Arch Med Sci. 2019;15(5):1278 1287. (10.5114/aoms.2018.75606) 31572474 PMC6764315

[b290-tjg-35-S1-s1] Wroński M Cebulski W Pawłowski W Krasnodębski IW Słodkowski M . Walled-off necrosis: safety of watchful waiting. Dig Dis Sci. 2015;60(4):1081 1086. (10.1007/s10620-014-3395-9) 25326117 PMC4408372

[b291-tjg-35-S1-s1] Sarathi Patra PP Das K Bhattacharyya A , et al. Natural resolution or intervention for fluid collections in acute severe pancreatitis. Br J Surg. 2014;101(13):1721 1728. (10.1002/bjs.9666) 25329330

[293] Rana SS Sharma R Dhalaria L Kang M Gupta R . A case series of late gastrointestinal fistulization in 16 patients with walled-off necrosis. Dig Dis Sci. 2022;67(2):661 666. (10.1007/s10620-021-06900-y) 33651255

[294] van Santvoort HC Besselink MG Bakker OJ , et al. A step-up approach or open necrosectomy for necrotizing pancreatitis. N Engl J Med. 2010;362(16):1491 1502. (10.1056/NEJMoa0908821) 20410514

[295] van Brunschot S van Grinsven J van Santvoort HC , et al. Endoscopic or surgical step-up approach for infected necrotising pancreatitis: a multicentre randomised trial. Lancet. 2018;391(10115):51 58. (10.1016/S0140-6736(17)32404-2) 29108721

[296] Bang JY Arnoletti JP Holt BA , et al. An endoscopic transluminal approach, compared with minimally invasive surgery, reduces complications and costs for patients with necrotizing pancreatitis. Gastroenterology. 2019;156(4):1027 1040.e3. (10.1053/j.gastro.2018.11.031) 30452918

[297] Szakó L Mátrai P Hegyi P , et al. Endoscopic and surgical drainage for pancreatic fluid collections are better than percutaneous drainage: meta-analysis. Pancreatology. 2020;20(1):132 141. (10.1016/j.pan.2019.10.006) 31706819

[298] van Baal MC van Santvoort HC Bollen TL , et al. Systematic review of percutaneous catheter drainage as primary treatment for necrotizing pancreatitis. Br J Surg. 2011;98(1):18 27. (10.1002/bjs.7304) 21136562

[299] Tann M Maglinte D Howard TJ , et al. Disconnected pancreatic duct syndrome: imaging findings and therapeutic implications in 26 surgically corrected patients. J Comput Assist Tomogr. 2003;27(4):577 582. (10.1097/00004728-200307000-00023) 12886147

[b299-tjg-35-S1-s1] Fischer TD Gutman DS Hughes SJ Trevino JG Behrns KE . Disconnected pancreatic duct syndrome: disease classification and management strategies. J Am Coll Surg. 2014;219(4):704 712. (10.1016/j.jamcollsurg.2014.03.055) 25065360

[301] Sandrasegaran K Tann M Jennings SG , et al. Disconnection of the pancreatic duct: an important but overlooked complication of severe acute pancreatitis. RadioGraphics. 2007;27(5):1389 1400. (10.1148/rg.275065163) 17848698

[302] Gupta K Freeman ML . Disconnected pancreatic duct with pancreas necrosis, treated with transgastric debridement and pancreatic duct stent. Clin Gastroenterol Hepatol. 2010;8(5):e51. (10.1016/j.cgh.2009.11.019) 20005979

[303] Maatman TK Roch AM Lewellen KA , et al. Disconnected pancreatic duct syndrome: spectrum of operative management. J Surg Res. 2020;247:297 303. (10.1016/j.jss.2019.09.068) 31685250

[304] Verma S Rana SS . Disconnected pancreatic duct syndrome: updated review on clinical implications and management. Pancreatology. 2020;20(6):1035 1044. (10.1016/j.pan.2020.07.402) 32800651

[305] Nealon WH Bhutani M Riall TS Raju G Ozkan O Neilan R . A unifying concept: pancreatic ductal anatomy both predicts and determines the major complications resulting from pancreatitis. J Am Coll Surg. 2009;208(5):790 799; discussion 799 801. (10.1016/j.jamcollsurg.2008.12.027) 19476839

[306] Hamada T Iwashita T Saito T , et al. Disconnected pancreatic duct syndrome and outcomes of endoscopic ultrasound-guided treatment of pancreatic fluid collections: systematic review and meta-analysis. Dig Endosc. 2022;34(4):676 686. (10.1111/den.14142) 34544204

[307] Bang JY Navaneethan U Hasan MK Sutton B Hawes R Varadarajulu S . Non-superiority of lumen-apposing metal stents over plastic stents for drainage of walled-off necrosis in a randomised trial. Gut. 2019;68(7):1200 1209. (10.1136/gutjnl-2017-315335) 29858393 PMC6582745

[308] Rana SS . An overview of walled-off pancreatic necrosis for clinicians. Expert Rev Gastroenterol Hepatol. 2019;13(4):331 343. (10.1080/17474124.2019.1574568) 30791769

[309] Chong E Ratnayake CB Saikia S , et al. Endoscopic transmural drainage is associated with improved outcomes in disconnected pancreatic duct syndrome: a systematic review and meta-analysis. BMC Gastroenterol. 2021;21(1):87. (10.1186/s12876-021-01663-2) 33632128 PMC7905849

[310] van Dijk SM Timmerhuis HC Verdonk RC , et al. Treatment of disrupted and disconnected pancreatic duct in necrotizing pancreatitis: a systematic review and meta-analysis. Pancreatology. 2019;19(7):905 915. (10.1016/j.pan.2019.08.006) 31473083

[311] Pelaez-Luna M Vege SS Petersen BT , et al. Disconnected pancreatic duct syndrome in severe acute pancreatitis: clinical and imaging characteristics and outcomes in a cohort of 31 cases. Gastrointest Endosc. 2008;68(1):91 97. (10.1016/j.gie.2007.11.041) 18378234

[b311-tjg-35-S1-s1] Arvanitakis M Delhaye M Bali MA , et al. Pancreatic-fluid collections: a randomized controlled trial regarding stent removal after endoscopic transmural drainage. Gastrointest Endosc. 2007;65(4):609 619. (10.1016/j.gie.2006.06.083) 17324413

[b312-tjg-35-S1-s1] Bang JY Wilcox CM Navaneethan U , et al. Impact of disconnected pancreatic duct syndrome on the endoscopic management of pancreatic fluid collections. Ann Surg. 2018;267(3):561 568. (10.1097/SLA.0000000000002082) 27849658

[b313-tjg-35-S1-s1] Rana SS Shah J Sharma RK Gupta R . Clinical and morphological consequences of permanent indwelling transmural plastic stents in disconnected pancreatic duct syndrome. Endosc Ultrasound. 2020;9(2):130 137. (10.4103/eus.eus_8_20) 32295971 PMC7279086

[315] Téllez-Aviña FI Casasola-Sánchez LE Ramírez-Luna MÁ , et al. Permanent indwelling transmural stents for endoscopic treatment of patients with disconnected pancreatic duct syndrome: long-term results. J Clin Gastroenterol. 2018;52(1):85 90. (10.1097/MCG.0000000000000754) 27824641

[316] Drake LM Anis M Lawrence C . Accuracy of magnetic resonance cholangiopancreatography in identifying pancreatic duct disruption. J Clin Gastroenterol. 2012;46(8):696 699. (10.1097/MCG.0b013e31825003b3) 22565603

[317] Anis FS Adiamah A Lobo DN Sanyal S . Incidence and treatment of splanchnic vein thrombosis in patients with acute pancreatitis: a systematic review and meta-analysis. J Gastroenterol Hepatol. 2022;37(3):446 454. (10.1111/jgh.15711) 34657310

[318] Xu W Qi X Chen J Su C Guo X . Prevalence of splanchnic vein thrombosis in pancreatitis: a systematic review and meta-analysis of observational studies. Gastroenterol Res Pract. 2015;2015:245460. (10.1155/2015/245460) 26451142 PMC4584234

[319] Zhou J Zhang H Mao W , et al. Efficacy and safety of early systemic anticoagulation for preventing splanchnic thrombosis in acute necrotizing pancreatitis. Pancreas. 2020;49(9):1220 1224. (10.1097/MPA.0000000000001661) 32898006

[320] Sissingh NJ Groen JV Koole D , et al. Therapeutic anticoagulation for splanchnic vein thrombosis in acute pancreatitis: a systematic review and meta-analysis. Pancreatology. 2022;22(2):235 243. (10.1016/j.pan.2021.12.008) 35012902

[321] Nawacki Ł Matykiewicz J Stochmal E Głuszek S . Splanchnic vein thrombosis in acute pancreatitis and its consequences. Clin Appl Thromb Hemost. 2021;27:10760296211010260. (10.1177/10760296211010260) 33887991 PMC8072901

[322] Fei Y Hu J Li WQ Wang W Zong GQ . Artificial neural networks predict the incidence of portosplenomesenteric venous thrombosis in patients with acute pancreatitis. J Thromb Haemost. 2017;15(3):439 445. (10.1111/jth.13588) 27960048

[323] Pancreas Study Group, Chinese Society of Gastroenterology, Chinese Medical Association, Chinese Society of Gastroenterology, Chinese Medical Association. Practice guidance for diagnosis and treatment of pancreatitis-related splanchnic vein thrombosis (Shenyang, 2020). J Dig Dis. 2021;22(1):2 8. (10.1111/1751-2980.12962) 33215862

[324] Easler J Muddana V Furlan A , et al. Portosplenomesenteric venous thrombosis in patients with acute pancreatitis is associated with pancreatic necrosis and usually has a benign course. Clin Gastroenterol Hepatol. 2014;12(5):854 862. (10.1016/j.cgh.2013.09.068) 24161350

[325] Norton W Lazaraviciute G Ramsay G Kreis I Ahmed I Bekheit M . Current practice of anticoagulant in the treatment of splanchnic vein thrombosis secondary to acute pancreatitis. Hepatobiliary Pancreat Dis Int. 2020;19(2):116 121. (10.1016/j.hbpd.2019.12.007) 31954635

[326] Primignani M . Pancreatitis-related splanchnic vein thrombosis: what role, what warnings for anticoagulation therapy? J Dig Dis. 2021;22(3):122 125. (10.1111/1751-2980.12974) 33591597

[327] Tozlu M Kayar Y İnce AT Baysal B Şentürk H . Low molecular weight heparin treatment of acute moderate and severe pancreatitis: a randomized, controlled,open-label study. Turk J Gastroenterol. 2019;30(1):81 87. (10.5152/tjg.2018.18583) 30289392 PMC6389310

[328] Qiu Q Li GJ Tang L , et al. The efficacy of low molecular weight heparin in severe acute pancreatitis: a systematic review and meta-analysis of randomized controlled trials. J Dig Dis. 2019;20(10):512 522. (10.1111/1751-2980.12815) 31432611

[329] Khurana V Ganguly I . Recurrent acute pancreatitis. JOP. 2014;15(5):413 426. (10.6092/1590-8577/2417) 25262707

[b329-tjg-35-S1-s1] Guda NM Muddana V Whitcomb DC , et al. Recurrent acute pancreatitis: international state-of-the-science conference with recommendations. Pancreas. 2018;47(6):653 666. (10.1097/MPA.0000000000001053) 29894415

[b330-tjg-35-S1-s1] Guda NM Trikudanathan G Freeman ML . Idiopathic recurrent acute pancreatitis. Lancet Gastroenterol Hepatol. 2018;3(10):720 728. (10.1016/S2468-1253(18)30211-5) 30215363

[b331-tjg-35-S1-s1] Kedia S Dhingra R Garg PK . Recurrent acute pancreatitis: an approach to diagnosis and management. Trop Gastroenterol. 2013;34(3):123 135. (10.7869/tg.116) 24851521

[333] Jagannath S Garg PK . Recurrent acute pancreatitis: current concepts in the diagnosis and management. Curr Treat Options Gastroenterol. 2018;16(4):449 465. (10.1007/s11938-018-0196-9) 30232693

[b333-tjg-35-S1-s1] Ahmed Ali U Issa Y Hagenaars JC , et al. Risk of recurrent pancreatitis and progression to chronic pancreatitis after a first episode of acute pancreatitis. Clin Gastroenterol Hepatol. 2016;14(5):738 746. (10.1016/j.cgh.2015.12.040) 26772149

[335] Yadav D O’Connell M Papachristou GI . Natural history following the first attack of acute pancreatitis. Am J Gastroenterol. 2012;107(7):1096 1103. (10.1038/ajg.2012.126) 22613906

[336] Umans DS Hallensleben ND Verdonk RC , et al. Recurrence of idiopathic acute pancreatitis after cholecystectomy: systematic review and meta-analysis. Br J Surg. 2020;107(3):191 199. (10.1002/bjs.11429) 31875953 PMC7003758

[337] Yu B Li J Li N , et al. Progression to recurrent acute pancreatitis after a first attack of acute pancreatitis in adults. Pancreatology. 2020;20(7):1340 1346. (10.1016/j.pan.2020.09.006) 32972837

[338] Magnusdottir BA Baldursdottir MB Kalaitzakis E Björnsson ES . Risk factors for chronic and recurrent pancreatitis after first attack of acute pancreatitis. Scand J Gastroenterol. 2019;54(1):87 94. (10.1080/00365521.2018.1550670) 30700163

[339] Lankisch PG Breuer N Bruns A Weber-Dany B Lowenfels AB Maisonneuve P . Natural history of acute pancreatitis: a longterm population-based study. Am J Gastroenterol. 2009;104(11):2797 805; quiz 2806. (10.1038/ajg.2009.405) 19603011

[b339-tjg-35-S1-s1] Cavestro GM Leandro G Di Leo M , et al. A single-centre prospective, cohort study of the natural history of acute pancreatitis. Dig Liver Dis. 2015;47(3):205 210. (10.1016/j.dld.2014.11.003) 25475611

[341] Takeyama Y . Long-term prognosis of acute pancreatitis in Japan. Clin Gastroenterol Hepatol. 2009;7(11)(suppl):S15 S17. (10.1016/j.cgh.2009.08.022) 19896091

[342] Yadav D Hawes RH Brand RE , et al. Alcohol consumption, cigarette smoking and the risk of recurrent acute and chronic pancreatitis. Arch Intern Med. 2009;169(11):1035 1045. (10.1001/archinternmed.2009.125) 19506173 PMC6785300

[343] Setiawan VW Pandol SJ Porcel J , et al. Prospective study of alcohol drinking, smoking, and pancreatitis: the multiethnic cohort. Pancreas. 2016;45(6):819 825. (10.1097/MPA.0000000000000657) 27171516 PMC4905778

[344] Munigala S Conwell DL Gelrud A Agarwal B . Heavy smoking is associated with lower age at first episode of acute pancreatitis and a higher risk of recurrence. Pancreas. 2015;44(6):876 881. (10.1097/MPA.0000000000000364) 25906444

[345] Bertilsson S Swärd P Kalaitzakis E . Factors that affect disease progression after first attack of acute pancreatitis. Clin Gastroenterol Hepatol. 2015;13(9):1662 9.e3. (10.1016/j.cgh.2015.04.012) 25911118

[346] Sun Y Jin J Zhu A , et al. Risk factors for recurrent pancreatitis after first episode of acute pancreatitis. Int J Gen Med. 2022;15:1319 1328. (10.2147/IJGM.S344863) 35173470 PMC8841459

[347] Xiang JX Hu LS Liu P , et al. Impact of cigarette smoking on recurrence of hyperlipidemic acute pancreatitis. World J Gastroenterol. 2017;23(47):8387 8394. (10.3748/wjg.v23.i47.8387) 29307998 PMC5743509

[348] Cho JH Jeong YH Kim KH Kim TN . Risk factors of recurrent pancreatitis after first acute pancreatitis attack: a retrospective cohort study. Scand J Gastroenterol. 2020;55(1):90 94. (10.1080/00365521.2019.1699598) 31822144

[349] Vipperla K Somerville C Furlan A , et al. Clinical profile and natural course in a large cohort of patients with hypertriglyceridemia and pancreatitis. J Clin Gastroenterol. 2017;51(1):77 85. (10.1097/MCG.0000000000000579) 27322530

[350] Christian JB Arondekar B Buysman EK Jacobson TA Snipes RG Horwitz RI . Determining triglyceride reductions needed for clinical impact in severe hypertriglyceridemia. Am J Med. 2014;127(1):36 44.e1. (10.1016/j.amjmed.2013.09.018) 24384100

[351] Zafrir B Saliba W Jubran A Hijazi R Shapira C . Severe hypertriglyceridemia-related pancreatitis: characteristics and predictors of recurrence. Pancreas. 2019;48(2):182 186. (10.1097/MPA.0000000000001235) 30629026

[352] Garg SK Campbell JP Anugwom C , et al. Incidence and predictors of readmissions in acute pancreatitis: a nationwide analysis. Pancreas. 2018;47(1):46 54. (10.1097/MPA.0000000000000952) 29215534

[353] Mustafa A Begaj I Deakin M , et al. Long-term effectiveness of cholecystectomy and endoscopic sphincterotomy in the management of gallstone pancreatitis. Surg Endosc. 2014;28(1):127 133. (10.1007/s00464-013-3138-6) 23982647

[354] Kamal A Akhuemonkhan E Akshintala VS Singh VK Kalloo AN Hutfless SM . Effectiveness of guideline-recommended cholecystectomy to prevent recurrent pancreatitis. Am J Gastroenterol. 2017;112(3):503 510. (10.1038/ajg.2016.583) 28071655

[355] Stigliano S Belisario F Piciucchi M Signoretti M Delle Fave G Capurso G . Recurrent biliary acute pancreatitis is frequent in a real-world setting. Dig Liver Dis. 2018;50(3):277 282. (10.1016/j.dld.2017.12.011) 29311029

[356] Pelli H Lappalainen-Lehto R Piironen A Sand J Nordback I . Risk factors for recurrent acute alcohol-associated pancreatitis: a prospective analysis. Scand J Gastroenterol. 2008;43(5):614 621. (10.1080/00365520701843027) 18415757

[357] Gurakar M Jalaly NY Faghih M , et al. Impact of genetic testing and smoking on the distribution of risk factors in patients with recurrent acute and chronic pancreatitis. Scand J Gastroenterol. 2022;57(1):91 98. (10.1080/00365521.2021.1984573) 34663162 PMC9278560

[358] Abu-El-Haija M Valencia CA Hornung L , et al. Genetic variants in acute, acute recurrent and chronic pancreatitis affect the progression of disease in children. Pancreatology. 2019;19(4):535 540. (10.1016/j.pan.2019.05.001) 31088717 PMC6589390

[359] Masamune A Ariga H Kume K , et al. Genetic background is different between sentinel and recurrent acute pancreatitis. J Gastroenterol Hepatol. 2011;26(6):974 978. (10.1111/j.1440-1746.2011.06691.x) 21303407

[360] Aoun E Muddana V Papachristou GI Whitcomb DC . SPINK1 N34S is strongly associated with recurrent acute pancreatitis but is not a risk factor for the first or sentinel acute pancreatitis event. Am J Gastroenterol. 2010;105(2):446 451. (10.1038/ajg.2009.630) 19888199

[361] Seppänen H Puolakkainen P . Classification, severity assessment, and prevention of recurrences in acute pancreatitis. Scand J Surg. 2020;109(1):53 58. (10.1177/1457496920910007) 32192420

[362] Testoni PA . Acute recurrent pancreatitis: etiopathogenesis, diagnosis and treatment. World J Gastroenterol. 2014;20(45):16891 16901. (10.3748/wjg.v20.i45.16891) 25493002 PMC4258558

[363] Saraswat VA Sharma BC Agarwal DK Kumar R Negi TS Tandon RK . Biliary microlithiasis in patients with idiopathic acute pancreatitis and unexplained biliary pain: response to therapy. J Gastroenterol Hepatol. 2004;19(10):1206 1211. (10.1111/j.1440-1746.2004.03462.x) 15377301

[364] Venneman NG vanBerge-Henegouwen GP van Erpecum KJ . Pharmacological manipulation of biliary water and lipids: potential consequences for prevention of acute biliary pancreatitis. Curr Drug Targets Immune Endocr Metabol Disord. 2005;5(2):193 198. (10.2174/1568008054064814) 16089352

[365] Matsubayashi H Ishiwatari H Imai K , et al. Steroid therapy and steroid response in autoimmune pancreatitis. Int J Mol Sci. 2019;21(1):257. (10.3390/ijms21010257) 31905944 PMC6981453

[366] Huang W de la Iglesia-García D Baston-Rey I , et al. Exocrine pancreatic insufficiency following acute pancreatitis: systematic review and meta-analysis. Dig Dis Sci. 2019;64(7):1985 2005. (10.1007/s10620-019-05568-9) 31161524 PMC6584228

[b366-tjg-35-S1-s1] Abu-El-Haija M Uc A Werlin SL , et al. Nutritional considerations in pediatric pancreatitis: a position paper from the NASPGHAN pancreas committee and ESPGHAN cystic fibrosis/pancreas working group. J Pediatr Gastroenterol Nutr. 2018;67(1):131 143. (10.1097/MPG.0000000000002023) 29927872 PMC6020697

[368] Moggia E Koti R Belgaumkar AP , et al. Pharmacological interventions for acute pancreatitis. Cochrane Database Syst Rev. 2017;4(4):CD011384. (10.1002/14651858.CD011384.pub2) 28431202 PMC6478067

[369] Morris-Stiff GJ Bowrey DJ Oleesky D Davies M Clark GW Puntis MC . The antioxidant profiles of patients with recurrent acute and chronic pancreatitis. Am J Gastroenterol. 1999;94(8):2135 2140. (10.1111/j.1572-0241.1999.01311.x) 10445540

[370] Gooshe M Abdolghaffari AH Nikfar S Mahdaviani P Abdollahi M . Antioxidant therapy in acute, chronic and post-endoscopic retrograde cholangiopancreatography pancreatitis: an updated systematic review and meta-analysis. World J Gastroenterol. 2015;21(30):9189 9208. (10.3748/wjg.v21.i30.9189) 26290647 PMC4533052

[371] Uden S Bilton D Nathan L Hunt LP Main C Braganza JM . Antioxidant therapy for recurrent pancreatitis: placebo-controlled trial. Aliment Pharmacol Ther. 1990;4(4):357 371. (10.1111/j.1365-2036.1990.tb00482.x) 2103755

[372] Rada G Neumann I Roa M Rojas L . Antioxidants for acute pancreatitis. Cochrane Database Syst Rev. 2011;3:CD009049. (10.1111/j.1365-2036.1990.tb00482.x)

[373] da Costa DW Bouwense SA Schepers NJ , et al. Same-admission versus interval cholecystectomy for mild gallstone pancreatitis (PONCHO): a multicentre randomised controlled trial. Lancet. 2015;386(10000):1261 1268. (10.1016/S0140-6736(15)00274-3) 26460661

[374] Overby DW Apelgren KN Richardson W Fanelli R , Society of American Gastrointestinal and Endoscopic Surgeons. SAGES guidelines for the clinical application of laparoscopic biliary tract surgery. Surg Endosc. 2010;24(10):2368 2386. (10.1007/s00464-010-1268-7) 20706739

[375] Qayed E Shah R Haddad YK . Endoscopic retrograde cholangiopancreatography decreases All-Cause and pancreatitis readmissions in patients with acute gallstone pancreatitis who do not undergo cholecystectomy: a nationwide 5-year analysis. Pancreas. 2018;47(4):425 435. (10.1097/MPA.0000000000001033) 29517638

[376] Hwang SS Li BH Haigh PI . Gallstone pancreatitis without cholecystectomy. JAMA Surg. 2013;148(9):867 872. (10.1001/jamasurg.2013.3033) 23884515

[377] Hernandez V Pascual I Almela P , et al. Recurrence of acute gallstone pancreatitis and relationship with cholecystectomy or endoscopic sphincterotomy. Am J Gastroenterol. 2004;99(12):2417 2423. (10.1111/j.1572-0241.2004.40896.x) 15571590

[378] García de la Filia Molina I García García de Paredes A Martínez Ortega A , et al. Biliary sphincterotomy reduces the risk of acute gallstone pancreatitis recurrence in non-candidates for cholecystectomy. Dig Liver Dis. 2019;51(11):1567 1573. (10.1016/j.dld.2019.05.007) 31151894

[379] Vázquez-Lglesias JL González-Conde B López-Rosés L , et al. Endoscopic sphincterotomy for prevention of the recurrence of acute biliary pancreatitis in patients with gallbladder in situ: long-term follow-up of 88 patients. Surg Endosc. 2004;18(10):1442 1446. (10.1007/s00464-003-9185-7) 15791366

[380] Sharma VK Howden CW . Metaanalysis of randomized controlled trials of endoscopic retrograde cholangiography and endoscopic sphincterotomy for the treatment of acute biliary pancreatitis. Am J Gastroenterol. 1999;94(11):3211 3214. (10.1111/j.1572-0241.1999.01520.x) 10566716

[381] Liao Z Gao R Wang W , et al. A systematic review on endoscopic detection rate, endotherapy, and surgery for pancreas divisum. Endoscopy. 2009;41(5):439 444. (10.1055/s-0029-1214505) 19337962

[382] Morgan DE Logan K Baron TH Koehler RE Smith JK . Pancreas divisum: implications for diagnostic and therapeutic pancreatography. AJR Am J Roentgenol. 1999;173(1):193 198. (10.2214/ajr.173.1.10397125) 10397125

[383] Bernard JP Sahel J Giovannini M Sarles H . Pancreas divisum is a probable cause of acute pancreatitis: a report of 137 cases. Pancreas. 1990;5(3):248 254. (10.1097/00006676-199005000-00002) 2343039

[384] Kanth R Samji NS Inaganti A , et al. Endotherapy in symptomatic pancreas divisum: a systematic review. Pancreatology. 2014;14(4):244 250. (10.1016/j.pan.2014.05.796) 25062871

[385] Guo A Poneros JM . The role of endotherapy in recurrent acute pancreatitis. Gastrointest Endosc Clin N Am. 2018;28(4):455 476. (10.1016/j.giec.2018.05.001) 30241638

[386] Roberts JR Romagnuolo J . Endoscopic therapy for acute recurrent pancreatitis. Gastrointest Endosc Clin N Am. 2013;23(4):803 819. (10.1016/j.giec.2013.06.006) 24079791

[387] Tringali A Voiosu T Schepis T , et al. Pancreas divisum and recurrent pancreatitis: long-term results of minor papilla sphincterotomy. Scand J Gastroenterol. 2019;54(3):359 364. (10.1080/00365521.2019.1584640) 30880501

[388] Fogel EL Toth TG Lehman GA DiMagno MJ DiMagno EP . Does endoscopic therapy favorably affect the outcome of patients who have recurrent acute pancreatitis and pancreas divisum? Pancreas. 2007;34(1):21 45. (10.1097/mpa.0b013e31802ce068) 17198181

[389] Lans JI Geenen JE Johanson JF Hogan WJ . Endoscopic therapy in patients with pancreas divisum and acute pancreatitis: a prospective, randomized, controlled clinical trial. Gastrointest Endosc. 1992;38(4):430 434. (10.1016/s0016-5107(92)70471-4) 1511816

[390] Michailidis L Aslam B Grigorian A Mardini H . The efficacy of endoscopic therapy for pancreas divisum: a meta-analysis. Ann Gastroenterol. 2017;30(5):550 558. (10.20524/aog.2017.0159) 28845111 PMC5566776

[391] Buxbaum J . The role of endoscopic retrograde cholangiopancreatography in patients with pancreatic disease. Gastroenterol Clin North Am. 2012;41(1):23 45. (10.1016/j.gtc.2011.12.010) 22341248

[392] Romagnuolo J Durkalski V Fogel EL , et al. Mo1428 Prospective study of imaging predictive value and agreement in diagnosing pancreas divisum: the FRAMES (Frequency of Recurrent Acute Pancreatitis After Minor Papilla Endoscopic sphincterotomy) study [abstract]. Gastrointest Endosc. 2013;77(5). (10.1016/j.gie.2013.03.176)

[393] Mariani A Di Leo M Petrone MC , et al. Outcome of endotherapy for pancreas divisum in patients with acute recurrent pancreatitis. World J Gastroenterol. 2014;20(46):17468 17475. (10.3748/wjg.v20.i46.17468) 25516660 PMC4265607

[394] Zakko L Gardner TB . Endoscopic management of recurrent acute pancreatitis. Clin Gastroenterol Hepatol. 2019;17(11):2167 2170. (10.1016/j.cgh.2019.04.069) 31077835

[b394-tjg-35-S1-s1] Elta GH . Sphincter of Oddi dysfunction and bile duct microlithiasis in acute idiopathic pancreatitis. World J Gastroenterol. 2008;14(7):1023 1026. (10.3748/wjg.14.1023) 18286682 PMC2689403

[396] Coyle WJ Pineau BC Tarnasky PR , et al. Evaluation of unexplained acute and acute recurrent pancreatitis using endoscopic retrograde cholangiopancreatography, sphincter of Oddi manometry and endoscopic ultrasound. Endoscopy. 2002;34(8):617 623. (10.1055/s-2002-33245) 12173081

[397] Fazel A Geenen JE MoezArdalan K Catalano MF . Intrapancreatic ductal pressure in sphincter of Oddi dysfunction. Pancreas. 2005;30(4):359 362. (10.1097/01.mpa.0000160278.11086.89) 15841048

[398] Yaghoobi M Romagnuolo J . Sphincter of Oddi dysfunction: updates from the recent literature. Curr Gastroenterol Rep. 2015;17(8):31. (10.1007/s11894-015-0455-z) 26143628

[399] Wilcox CM . Sphincter of Oddi dysfunction Type III: new studies suggest new approaches are needed. World J Gastroenterol. 2015;21(19):5755 5761. (10.3748/wjg.v21.i19.5755) 26019439 PMC4438009

[400] Kaw M Brodmerkel GJ Jr . ERCP, biliary crystal analysis, and sphincter of Oddi manometry in idiopathic recurrent pancreatitis. Gastrointest Endosc. 2002;55(2):157 162. (10.1067/mge.2002.118944) 11818915

[401] Testoni PA Caporuscio S Bagnolo F Lella F . Idiopathic recurrent pancreatitis: long-term results after ERCP, endoscopic sphincterotomy, or ursodeoxycholic acid treatment. Am J Gastroenterol. 2000;95(7):1702 1707. (10.1111/j.1572-0241.2000.02292.x) 10925971

[402] Wehrmann T . Long-term results (≥ 10 years) of endoscopic therapy for sphincter of Oddi dysfunction in patients with acute recurrent pancreatitis. Endoscopy. 2011;43(3):202 207. (10.1055/s-0030-1255922) 21108172

[403] Hall TC Dennison AR Garcea G . The diagnosis and management of sphincter of Oddi dysfunction: a systematic review. Langenbecks Arch Surg. 2012;397(6):889 898. (10.1007/s00423-012-0971-3) 22688754

[404] Coté GA Imperiale TF Schmidt SE , et al. Similar efficacies of biliary, with or without pancreatic, sphincterotomy in treatment of idiopathic recurrent acute pancreatitis. Gastroenterology. 2012;143(6):1502 1509.e1. (10.1053/j.gastro.2012.09.006) 22982183

[405] Varadarajulu S Wilcox CM . Randomized trial comparing needle-knife and pull-sphincterotome techniques for pancreatic sphincterotomy in high-risk patients. Gastrointest Endosc. 2006;64(5):716 722. (10.1016/j.gie.2006.02.058) 17055863

[406] Das R Clarke B Tang G , et al. Endoscopic sphincterotomy (ES) may not alter the natural history of idiopathic recurrent acute pancreatitis (IRAP). Pancreatology. 2016;16(5):770 777. (10.1016/j.pan.2016.07.009) 27450967

[407] Somani P Navaneethan U . Role of ERCP in patients with idiopathic recurrent acute pancreatitis. Curr Treat Options Gastroenterol. 2016;14(3):327 339. (10.1007/s11938-016-0096-9) 27371265

[408] Jacob L Geenen JE Catalano MF Geenen DJ . Prevention of pancreatitis in patients with idiopathic recurrent pancreatitis: a prospective nonblinded randomized study using endoscopic stents. Endoscopy. 2001;33(7):559 562. (10.1055/s-2001-15314) 11473324

[409] Hollemans RA Hallensleben NDL Mager DJ , et al. Pancreatic exocrine insufficiency following acute pancreatitis: systematic review and study level meta-analysis. Pancreatology. 2018;18(3):253 262. (10.1016/j.pan.2018.02.009) 29482892

[410] Czakó L Takács T Hegyi P , et al. Quality of life assessment after pancreatic enzyme replacement therapy in chronic pancreatitis. Can J Gastroenterol. 2003;17(10):597 603. (10.1155/2003/515848) 14571298

[411] Kahl S Schütte K Glasbrenner B , et al. The effect of oral pancreatic enzyme supplementation on the course and outcome of acute pancreatitis: a randomized, double-blind parallel-group study. JOP. 2014;15(2):165 174. (10.6092/1590-8577/797) 24618443

[b411-tjg-35-S1-s1] Soytürk M Bengi G Oğuz D , et al. Turkish Gastroenterology Association, Pancreas Study Group, chronic pancreatitis committee consensus report. Turk J Gastroenterol. 2020;31(suppl 1):S1 S41. (10.5152/tjg.2020.220920) 33210608 PMC7752168

[b412-tjg-35-S1-s1] Phillips ME Hopper AD Leeds JS , et al. Consensus for the management of pancreatic exocrine insufficiency: UK practical guidelines. BMJ Open Gastroenterol. 2021;8(1):e000643. (10.1136/bmjgast-2021-000643) PMC8212181 34140324

[414] Löhr JM Oliver MR Frulloni L . Synopsis of recent guidelines on pancreatic exocrine insufficiency. U Eur Gastroenterol J. 2013;1(2):79 83. (10.1177/2050640613476500) PMC4040730 24917944

[415] Ru N Zou WB Wu H , et al. Chinese guidelines for the diagnosis and treatment of pancreatic exocrine insufficiency (2018 edition). J Dig Dis. 2018 ed. 2019;20(11):567 571. (10.1111/1751-2980.12753) 31006979

[416] Nikfarjam M Wilson JS Smith RC , Australasian Pancreatic Club Pancreatic Enzyme Replacement Therapy Guidelines Working Group. Diagnosis and management of pancreatic exocrine insufficiency. Med J Aust. 2017;207(4):161 165. (10.5694/mja16.00851) 28814218

[b416-tjg-35-S1-s1] Pezzilli R Andriulli A Bassi C , et al. Exocrine pancreatic insufficiency in adults: a shared position statement of the Italian Association for the Study of the Pancreas. World J Gastroenterol. 2013;19(44):7930 7946. (10.3748/wjg.v19.i44.7930) 24307787 PMC3848141

[418] Rana SS Sharma RK Gupta R . Endoscopic management of pancreatic ascites due to duct disruption following acute necrotizing pancreatitis. JGH Open. 2019;3(2):111 116. (10.1002/jgh3.12113) 31061885 PMC6487829

[419] Komara NL Paragomi P Greer PJ , et al. Severe acute pancreatitis: capillary permeability model linking systemic inflammation to multiorgan failure. Am J Physiol Gastrointest Liver Physiol. 2020;319(5):G573 G583. (10.1152/ajpgi.00285.2020) 32877220 PMC8087347

[420] Bush N Rana SS . Ascites in acute pancreatitis: clinical implications and management. Dig Dis Sci. 2022;67(6):1987 1993. (10.1007/s10620-021-07063-6) 34036465

[421] Rana SS Bhasin DK Rao C Sharma R Gupta R . Consequences of long term indwelling transmural stents in patients with walled off pancreatic necrosis & disconnected pancreatic duct syndrome. Pancreatology. 2013;13(5):486 490. (10.1016/j.pan.2013.07.284) 24075512

[422] Kayode-Ajala F Jesani S Ejikeme C Asrar H Naqi M . A rare case of pancreatic ascites secondary to chronic pancreatitis. J Investig Med High Impact Case Rep. 2022;10:23247096221103379. (10.1177/23247096221103379) PMC9201292 35699233

[423] Varadarajulu S Rana SS Bhasin DK . Endoscopic therapy for pancreatic duct leaks and disruptions. Gastrointest Endosc Clin N Am. 2013;23(4):863 892. (10.1016/j.giec.2013.06.008) 24079795

[b423-tjg-35-S1-s1] Kanneganti K Srikakarlapudi S Acharya B Sindhaghatta V Chilimuri S . Successful management of pancreatic ascites with both conservative management and pancreatic duct stenting. Gastroenterology Res. 2009;2(4):245 247. (10.4021/gr2009.08.1306) 27942284 PMC5139751

[b424-tjg-35-S1-s1] Bracher GA Manocha AP DeBanto JR , et al. Endoscopic pancreatic duct stenting to treat pancreatic ascites. Gastrointest Endosc. 1999;49(6):710 715. (10.1016/s0016-5107(99)70287-7) 10343214

[b425-tjg-35-S1-s1] Bhasin DK Rana SS Siyad I , et al. Endoscopic transpapillary nasopancreatic drainage alone to treat pancreatic ascites and pleural effusion. J Gastroenterol Hepatol. 2006;21(6):1059 1064. (10.1111/j.1440-1746.2005.04049.x) 16724995

[b426-tjg-35-S1-s1] Pai CG Suvarna D Bhat G . Endoscopic treatment as first-line therapy for pancreatic ascites and pleural effusion. J Gastroenterol Hepatol. 2009;24(7):1198 1202. (10.1111/j.1440-1746.2009.05796.x) 19486258

[428] Lakhtakia S Reddy DN . Pancreatic leaks: endo-therapy first? J Gastroenterol Hepatol. 2009;24(7):1158 1160. (10.1111/j.1440-1746.2009.05926.x) 19682188

[429] Liu WH Ren LN Chen T ,et al. Abdominal paracentesis drainage ahead of percutaneous catheter drainage benefits patients attacked by acute pancreatitis with fluid collections: a retrospective clinical cohort study. Crit Care Med. 2015;43(1):109 119. (10.1097/CCM.0000000000000606) 25251762

[430] Liu L Yan H Liu W , et al. Abdominal paracentesis drainage does not increase infection in severe acute pancreatitis: a prospective study. J Clin Gastroenterol. 2015;49(9):757 763. (10.1097/MCG.0000000000000358) 26053169

[431] Rushforth JA Beck JM McMahon M Puntis JW . Resolution of pancreatic ascites with octreotide. Arch Dis Child. 1993;68(1):135 136. (10.1136/adc.68.1.135) 8434999 PMC1029202

[432] Gapp J Hoilat GJ Chandra S . Pancreatic ascites. StatPearls [Internet]; Updated 2022 August 14. In: Treasure Island (FL): StatPearls Publishing; 2024. Available at: https://www.ncbi.nlm.nih.gov/books/NBK507851/. 29939628

[b432-tjg-35-S1-s1] Pang TCY Maher R Gananadha S Hugh TJ Samra JS . Peripancreatic pseudoaneurysms: a management-based classification system. Surg Endosc. 2014;28(7):2027 2038. (10.1007/s00464-014-3434-9) 24519028 PMC4065337

[434] Grassedonio E Toia P La Grutta L , et al. Role of computed tomography and magnetic resonance imaging in local complications of acute pancreatitis. Gland Surg. 2019;8(2):123 132. (10.21037/gs.2018.12.07) 31183322 PMC6534767

[435] Kalas MA Leon M Chavez LO Canalizo E Surani S . Vascular complications of pancreatitis. World J Clin Cases. 2022;10(22):7665 7673. (10.12998/wjcc.v10.i22.7665) 36158481 PMC9372863

[436] Tarar ZI Khan HA Inayat F , et al. Hemosuccus Pancreaticus: a comprehensive review of presentation patterns, diagnostic approaches, therapeutic strategies, and clinical outcomes. J Investig Med High Impact Case Rep. 2022;10:23247096211070388. (10.1177/23247096211070388) PMC8796068 35045737

[437] Sagar S Soundarajan R Gupta P , et al. Efficacy of endovascular embolization of arterial pseudoaneurysms in pancreatitis: a systematic review and meta-analysis. Pancreatology. 2021;21(1):46 58. (10.1016/j.pan.2020.11.017) 33303372

[438] Bergert H Hinterseher I Kersting S Leonhardt J Bloomenthal A Saeger HD . Management and outcome of hemorrhage due to arterial pseudoaneurysms in pancreatitis. Surgery. 2005;137(3):323 328. (10.1016/j.surg.2004.10.009) 15746787

[439] Das SLM Singh PP Phillips ARJ Murphy R Windsor JA Petrov MS . Newly diagnosed diabetes mellitus after acute pancreatitis: a systematic review and meta-analysis. Gut. 2014;63(5):818 831. (10.1136/gutjnl-2013-305062) 23929695

[440] Das SLM Kennedy JIC Murphy R Phillips ARJ Windsor JA Petrov MS . Relationship between the exocrine and endocrine pancreas after acute pancreatitis. World J Gastroenterol. 2014;20(45):17196 17205. (10.3748/wjg.v20.i45.17196) 25493036 PMC4258592

[441] Aune D Mahamat-Saleh Y Norat T Riboli E . Diabetes mellitus and the risk of pancreatitis: a systematic review and meta-analysis of cohort studies. Pancreatology. 2020;20(4):602 607. (10.1016/j.pan.2020.03.019) 32409279

[442] Olesen SS Toledo FGS Hart PA . The spectrum of diabetes in acute and chronic pancreatitis. Curr Opin Gastroenterol. 2022;38(5):509 515. (10.1097/MOG.0000000000000864) 35881972 PMC9379856

[443] Petrov MS . DIAGNOSIS OF ENDOCRINE DISEASE: post-pancreatitis diabetes mellitus: prime time for secondary disease. Eur J Endocrinol. 2021;184(4):R137 R149. (10.1530/EJE-20-0468) 33460393

[444] Patra PS Das K . Longer-term outcome of acute pancreatitis: 5 years follow-up. JGH Open. 2021;5(12):1323 1327. (10.1002/jgh3.12679) 34950774 PMC8674540

[445] Nøjgaard C Becker U Matzen P Andersen JR Holst C Bendtsen F . Progression from acute to chronic pancreatitis: prognostic factors, mortality, and natural course. Pancreas. 2011;40(8):1195 1200. (10.1097/MPA.0b013e318221f569) 21926938

[446] Mueck KM Wei S Pedroza C , et al. Gallstone pancreatitis: admission versus normal cholecystectomy-a randomized trial (gallstone PANC trial). Ann Surg. 2019;270(3):519 527. (10.1097/SLA.0000000000003424) 31415304 PMC6949044

[b446-tjg-35-S1-s1] Wang L Yu HF Guo T Xie P Zhang ZW Yu YH . Early versus delayed laparoscopic cholecystectomy for acute cholecystitis with mild pancreatitis. Curr Med Sci. 2020;40(5):937 942. (10.1007/s11596-020-2275-0) 33123907

[b447-tjg-35-S1-s1] Berger S Taborda Vidarte CA Woolard S Morse B Chawla S . Same-admission cholecystectomy compared with delayed cholecystectomy in acute gallstone pancreatitis: outcomes and predictors in a safety net hospital cohort. South Med J. 2020;113(2):87 92. (10.14423/SMJ.0000000000001067) 32016439

[b448-tjg-35-S1-s1] Krishna SG Kruger AJ Patel N Hinton A Yadav D Conwell DL . Cholecystectomy during index admission for acute biliary pancreatitis lowers 30-day readmission rates. Pancreas. 2018;47(8):996 1002. (10.1097/MPA.0000000000001111) 30028444 PMC6203327

[450] Prabhu RY Irpatgire R Naranje B Kantharia CV Bapat RD Supe AN . Influence of timing on performance of laparoscopic cholecystectomy for acute biliary pancreatitis. Trop Gastroenterol. 2009;30(2):113 115. 19761000

[b450-tjg-35-S1-s1] Rosing DK de Virgilio C Yaghoubian A , et al. Early cholecystectomy for mild to moderate gallstone pancreatitis shortens hospital stay. J Am Coll Surg. 2007;205(6):762 766. (10.1016/j.jamcollsurg.2007.06.291) 18035259

[b451-tjg-35-S1-s1] Dubina ED de Virgilio C Simms ER Kim DY Moazzez A . Association of early vs delayed cholecystectomy for mild gallstone pancreatitis with perioperative outcomes. JAMA Surg. 2018 1;153(11):1057 1059. (10.1001/jamasurg.2018.2614) 30140844 PMC6583064

[b452-tjg-35-S1-s1] Degrate L Bernasconi DP Meroni P , et al. Mild acute biliary pancreatitis: the timing of cholecystectomy should not exceed index admission. Minerva Chir. 2017;72(5):383 390. (10.23736/S0026-4733.17.07356-4) 28425682

[b453-tjg-35-S1-s1] Aksoy F Demiral G Ekinci Ö . Can the timing of laparoscopic cholecystectomy after biliary pancreatitis change the conversion rate to open surgery? Asian J Surg. 2018;41(4):307 312. (10.1016/j.asjsur.2017.02.001) 28284749

[b454-tjg-35-S1-s1] Navarro-Sanchez A Ashrafian H Laliotis A Qurashi K Martinez-Isla A . Single-stage laparoscopic management of acute gallstone pancreatitis: outcomes at different timings. Hepatobiliary Pancreat Dis Int. 2016;15(3):297 301. (10.1016/s1499-3872(16)60065-6) 27298106

[b455-tjg-35-S1-s1] Mador BD Panton ONM Hameed SM . Early versus delayed cholecystectomy following endoscopic sphincterotomy for mild biliary pancreatitis. Surg Endosc. 2014;28(12):3337 3342. (10.1007/s00464-014-3621-8) 24962855

[b456-tjg-35-S1-s1] Li A Qin HJ Ke LW Chen G Lu HM Zhang ZD . Early or delayed cholecystectomy (LC) for acute gallstone pancreatitis? An experience and review. Hepatogastroenterology. 2012;59(119):2327 2329. (10.5754/hge12319) 22626856

[458] Nebiker CA Frey DM Hamel CT Oertli D Kettelhack C . Early versus delayed cholecystectomy in patients with biliary acute pancreatitis. Surgery. 2009;145(3):260 264. (10.1016/j.surg.2008.10.012) 19231577

[459] Griniatsos J Karvounis E Isla A . Early versus delayed single-stage laparoscopic eradication for both gallstones and common bile duct stones in mild acute biliary pancreatitis. Am Surg. 2005;71(8):682 686. (10.1177/000313480507100812) 16217952

[460] Aboulian A Chan T Yaghoubian A , et al. Early cholecystectomy safely decreases hospital stay in patients with mild gallstone pancreatitis: a randomized prospective study. Ann Surg. 2010;251(4):615 619. (10.1097/SLA.0b013e3181c38f1f) 20101174

[461] da Costa DW Dijksman LM Bouwense SA , et al. Cost-effectiveness of same-admission versus interval cholecystectomy after mild gallstone pancreatitis in the PONCHO trial. Br J Surg. 2016;103(12):1695 1703. (10.1002/bjs.10222) 27517163

[462] Jee SL Jarmin R Lim KF Raman K . Outcomes of early versus delayed cholecystectomy in patients with mild to moderate acute biliary pancreatitis: a randomized prospective study. Asian J Surg. 2018;41(1):47 54. (10.1016/j.asjsur.2016.07.010) 27530927

[463] Noel R Arnelo U Lundell L , et al. Index versus delayed cholecystectomy in mild gallstone pancreatitis: results of a randomized controlled trial. HPB (Oxford). 2018;20(10):932 938. (10.1016/j.hpb.2018.03.016) 29703649

[464] Riquelme F Marinkovic B Salazar M , et al. Early laparoscopic cholecystectomy reduces hospital stay in mild gallstone pancreatitis. A randomized controlled trial. HPB (Oxford). 2020;22(1):26 33. (10.1016/j.hpb.2019.05.013) 31235428

[465] Isbell KD Wei S Dodwad SM , et al. Impact of early cholecystectomy on the cost of treating mild gallstone pancreatitis: gallstone PANC trial. J Am Coll Surg. 2021;233(4):517 525.e1. (10.1016/j.jamcollsurg.2021.06.023) 34325019 PMC8856738

[466] Gurusamy KS Nagendran M Davidson BR . Early versus delayed laparoscopic cholecystectomy for acute gallstone pancreatitis. Cochrane Database Syst Rev. 2013;2013(9):CD010326. (10.1002/14651858.CD010326.pub2) 23996398 PMC11452085

[467] Nealon WH Bawduniak J Walser EM . Appropriate timing of cholecystectomy in patients who present with moderate to severe gallstone-associated acute pancreatitis with peripancreatic fluid collections. Ann Surg. 2004;239(6):741 749; discussion 749 751. (10.1097/01.sla.0000128688.97556.94) 15166953 PMC1356283

[468] Panek J Karcz D Rembiasz K Budzyński A Zasada J . Early endoscopic sphincterotomy and early laparoscopic cholecystectomy in the treatment of severe acute biliary pancreatitis--a preliminary report. Adv Med Sci. 2006;51:103 104. 17357286

[469] Sanjay P Yeeting S Whigham C Judson H Polignano FM Tait IS . Endoscopic sphincterotomy and interval cholecystectomy are reasonable alternatives to index cholecystectomy in severe acute gallstone pancreatitis (GSP). Surg Endosc. 2008;22(8):1832 1837. (10.1007/s00464-007-9710-1) 18071797

[470] Working Group IAP/APA Acute Pancreatitis Guidelines. IAP/APA evidence-based guidelines for the management of acute pancreatitis. Pancreatology. 2013;13(4)(suppl 2):e1 e15. (10.1016/j.pan.2013.07.063) 24054878

[471] Pezzilli R Zerbi A Di Carlo V Bassi C Delle Fave GF , Working Group of the Italian Association for the Study of the Pancreas on Acute Pancreatitis. Practical guidelines for acute pancreatitis. Pancreatology. 2010;10(5):523 535. (10.1159/000314602) 20975316

[472] Hughes DL Morris-Stiff G . Determining the optimal time interval for cholecystectomy in moderate to severe gallstone pancreatitis: a systematic review of published evidence. Int J Surg. 2020;84:171 179. (10.1016/j.ijsu.2020.11.016) 33227531

[473] Hallensleben ND Timmerhuis HC Hollemans RA , et al. Optimal timing of cholecystectomy after necrotising biliary pancreatitis. Gut. 2022;71(5):974 982. (10.1136/gutjnl-2021-324239) 34272261

[474] Levy MJ Geenen JE . Idiopathic acute recurrent pancreatitis. Am J Gastroenterol. 2001;96(9):2540 2555. (10.1111/j.1572-0241.2001.04098.x) 11569674

[475] Lee SP Nicholls JF Park HZ . Biliary sludge as a cause of acute pancreatitis. N Engl J Med. 1992;326(9):589 593. (10.1056/NEJM199202273260902) 1734248

[476] Ros E Navarro S Bru C Garcia-Pugés A Valderrama R . Occult microlithiasis in ‘idiopathic’ acute pancreatitis: prevention of relapses by cholecystectomy or ursodeoxycholic acid therapy. Gastroenterology. 1991;101(6):1701 1709. (10.1016/0016-5085(91)90410-m) 1955135

[477] Garg PK Tandon RK Madan K . Is biliary microlithiasis a significant cause of idiopathic recurrent acute pancreatitis? A long-term follow-up study. Clin Gastroenterol Hepatol. 2007;5(1):75 79. (10.1016/j.cgh.2006.06.023) 16931169

[478] Lei J Xu F Cao H Zhou Z He S . Cholecystectomy reduces the severity of subsequent idiopathic acute pancreatitis. Saudi J Gastroenterol. 2022;28(6):417 425. (10.4103/sjg.sjg_441_21) 36153928 PMC9843515

[479] Stevens CL Abbas SM Watters DAK . How does cholecystectomy influence recurrence of idiopathic acute pancreatitis? J Gastrointest Surg. 2016;20(12):1997 2001. (10.1007/s11605-016-3269-x) 27663692 PMC5104786

[480] Räty S Pulkkinen J Nordback I , et al. Can Laparoscopic cholecystectomy prevent recurrent idiopathic acute pancreatitis?: a prospective randomized multicenter trial. Ann Surg. 2015;262(5):736 741. (10.1097/SLA.0000000000001469) 26583660

[b480-tjg-35-S1-s1] Walkowska J Zielinska N Tubbs RS Podgórski M Dłubek-Ruxer J Olewnik Ł . Diagnosis and treatment of acute pancreatitis. Diagnostics (Basel). 2022;12(8):1974. (10.1097/SLA.0000000000001469) 36010324 PMC9406704

[482] Purschke B Bolm L Meyer MN Sato H . Interventional strategies in infected necrotizing pancreatitis: indications, timing, and outcomes. World J Gastroenterol. 2022;28(27):3383 3397. (10.3748/wjg.v28.i27.3383) 36158258 PMC9346450

[483] Heckler M Hackert T Hu K Halloran CM Büchler MW Neoptolemos JP . Severe acute pancreatitis: surgical indications and treatment. Langenbecks Arch Surg. 2021;406(3):521 535. (10.1007/s00423-020-01944-6) 32910276 PMC8106572

[484] Götzinger P Wamser P Exner R , et al. Surgical treatment of severe acute pancreatitis: timing of operation is crucial for survival. Surg Infect (Larchmt). 2003;4(2):205 211. (10.1089/109629603766957004) 12906721

[485] Lan L Luo J Yang X , et al. Association between timing of surgical intervention and mortality in 15,813 acute pancreatitis. Comput Math Methods Med. 2020;2020:1012796. (10.1089/109629603766957004) 32508973 PMC7246404

[486] Cheung MT Li WH Kwok PCH Hong JKF . Surgical management of pancreatic necrosis: towards lesser and later. J Hepatobil Pancreat Sci. 2010;17(3):338 344. (10.1007/s00534-009-0251-y) 20464564

[487] Harris HW Barcia A Schell MT Thoeni RF Schecter WP . Necrotizing pancreatitis: a surgical approach independent of documented infection. HPB (Oxford). 2004;6(3):161 168. (10.1080/13651820410033634) 18333070 PMC2020671

[488] Shenvi S Gupta R Kang M , et al. Timing of surgical intervention in patients of infected necrotizing pancreatitis not responding to percutaneous catheter drainage. Pancreatology. 2016;16(5):778 787. (10.1016/j.pan.2016.08.006) 27592206

[489] Götzinger P Sautner T Kriwanek S , et al. Surgical treatment for severe acute pancreatitis: extent and surgical control of necrosis determine outcome. World J Surg. 2002;26(4):474 478. (10.1007/s00268-001-0252-8) 11910483

[490] Gomatos IP Halloran CM Ghaneh P , et al. Outcomes from minimal access retroperitoneal and open pancreatic necrosectomy in 394 patients with necrotizing pancreatitis. Ann Surg. 2016;263(5):992 1001. (10.1097/SLA.0000000000001407) 26501713

[491] van Brunschot S Hollemans RA Bakker OJ , et al. Minimally invasive and endoscopic versus open necrosectomy for necrotising pancreatitis: a pooled analysis of individual data for 1980 patients. Gut. 2018;67(4):697 706. (10.1136/gutjnl-2016-313341) 28774886

[492] Besselink MGH van Santvoort HC Nieuwenhuijs VB , et al. Minimally invasive ‘step-up approach’ versus maximal necrosectomy in patients with acute necrotising pancreatitis (PANTER trial): design and rationale of a randomised controlled multicenter trial [ISRCTN13975868]. BMC Surg. 2006;6:6. (10.1186/1471-2482-6-6) 16606471 PMC1508161

