# Supplementary material for: Turkish Society of Gastroenterology: Pancreas Working Group, Acute Pancreatitis Committee Consensus Report
Source: Turk J Gastroenterol. 2024 Nov 1;35(Suppl 1):S1–S44. doi: 10.5152/tjg.2024.24392 (PMC11670795; doi:10.5152/tjg.2024.24392)
Supplement: Supplementary Material [file supplementary_material.pdf]

Supplementary Table 1. Level of Evidence Classification

Level of Evidence	Criteria
1a	Systematic reviews (meta-analyses) containing at least some trials of level 1b evidence, in which the results of separate, independently controlled trials are consistent
1b	Randomized controlled trial of good quality and of adequate sample size (power calculations)
2a	Randomized trials of reasonable quality and/or inadequate sample size
2b	Nonrandomized trials, comparative research (parallel cohort)
2c	Nonrandomized trials, comparative research (historical cohort, literature controls)
3	Nonrandomized, non-comparative trials, descriptive research
4	Expert opinions, including the opinion of work group members

Supplementary Table 2. Revised Atlanta Criteria for Severity of Acute Pancreatitis

**Severity Grades**	**Criteria**
Mild	• No organ failure<br>• No local or systemic complications
Moderate	• Transient organ failure (resolves within 48 hours) and/or<br>• Local or systemic complications without persistent organ failure
Severe	• Persistent organ failure (duration of >48 hours)<br>• Single organ failure<br>• Multiple organ failure

Local complications: Pancreatic/peripancreatic necrosis (sterile or infected), peripancreatic fluid collections, pseudocyst, walled-off necrosis (sterile or infected).

Supplementary Table 3. BISAP Scoring System

Blood urea nitrogen (BUN)	BUN >25 mg/dL (8.9 mmol/L) (1 point)
Impaired mental status	Abnormal mental status with a Glasgow coma score <15 (1 point)
SIRS	Evidence of SIRS (1 point)
Age	Age >60 years old (1 point)
Pleural effusion	Imaging study reveals pleural effusion (1 point)

0-2 points: Lower mortality (<2%)
3-5 points: Higher mortality (>15%)

SIRS (Systemic Inflammatory Response Syndrome) is diagnosed by the presence of any 2 of the following criteria:
1) Temperature (<36°C or >38°C),
2) Pulse >90/min,
3) Respiratory rate >20 or $PaCO_2$ <32mmHg, and
4) WBC > 12 000/mm^3 or <4000/mm^3 or >10% bands.
BUN, blood urea nitrogen; SIRS, systemic inflammatory response syndrome.

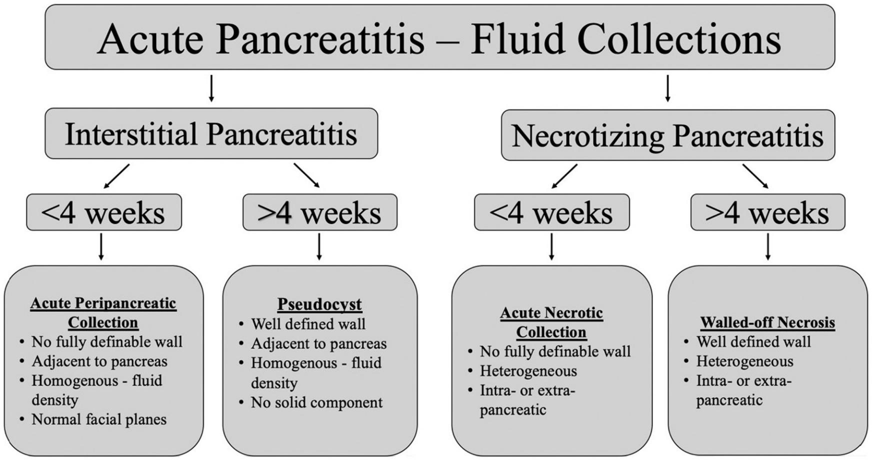

Supplementary Figure 1. Atlanta Classification of Pancreatic Fluid Collections.
